# Synthesis of Mesylated and Tosylated α-Hydroxy-Benzylphosphonates; Their Reactivity and Cytostatic Activity

**DOI:** 10.1021/acsomega.4c04382

**Published:** 2024-07-02

**Authors:** Zsuzsanna Szalai, Márton Debrei, Péter Ábrányi-Balogh, Szilvia Bősze, Rita Oláhné Szabó, Konstantin Karaghiosoff, László Drahos, György Keglevich

**Affiliations:** † Department of Organic Chemistry and Technology, Faculty of Chemical Technology and Biotechnology, Budapest University of Technology and Economics, Műegyetem rkp. 3, 1111 Budapest, Hungary; ‡ Medicinal Chemistry Research Group, HUN-REN Research Centre for Natural Sciences, 1117 Budapest, Hungary; § National Drug Research and Development Laboratory, HUN-REN Research Centre for Natural Sciences, 1117 Budapest, Hungary; ∥ Hungarian Research Network (HUN-REN), HUN-REN-ELTE Research Group of Peptide Chemistry, Eötvös Loránd University, 1117 Budapest, Hungary; ⊥ Department of Genetics, Cell- and Immunobiology, Semmelweis University, Nagyvárad tér 4, 1089 Budapest, Hungary; # Department Chemie, Ludwig-Maximilians-Universität München, Butenandtstr. 5-13, D-81377 München, Germany; ∇ MS Proteomics Research Group, Research Centre for Natural Sciences, 1117 Budapest, Hungary

## Abstract

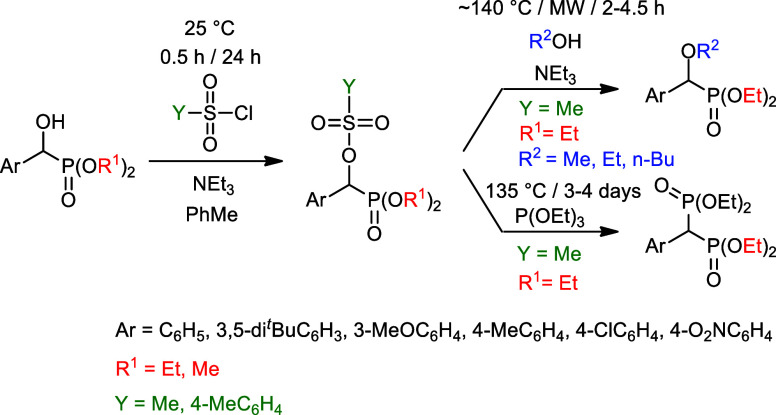

α-Hydroxyphosphonates and their acylated and phosphorylated derivatives may be of significant biological activity including cytotoxic effects. To extend the pool of the potentially bioactive species, new methane- and arenesulfonyloxyphosphonates were synthesized by the sulfonylation of differently substituted α-hydroxy-benzylphosphonates using methanesulfonyl chloride or *p*-toluenesulfonyl chloride at 25 °C in the presence of triethylamine in toluene. The new sulfonyl derivatives were obtained in 54–80% yields. In the case of the 4- or 2-methoxy substituent in the aromatic ring, surprisingly the corresponding α-chlorophosphonates were the exclusive products, whose formation was explained assuming a quinoid intermediate and supported by theoretical calculations. With a 3-methoxyphenyl substituent, the expected mesylation of the hydroxy group took place. Attempted alcoholyses of the diethyl α-methanesulfonyloxy-benzylphosphonates with different substituents in the benzyl ring at ∼140 °C in the presence of triethylamine under microwave irradiation left the P-function intact under the conditions applied, instead, the mesyloxy group was substituted by an alkoxy unit in a selective new reaction. The α-alkoxy-benzylphosphonates were isolated in 60–77% yields. While α-chloro- or α-bromo-benzylphosphonates proved to be rather inefficient in the Michaelis–Arbuzov reaction with triethyl phosphite, according to a new possibility, the α-methansulfonyloxy-benzylphosphonates underwent an efficient Arbuzov fission using the phosphite in excess at 135 °C. The arylmethylenebisphosphonates were obtained in yields of 76–81%. Bioactivity studies with the members of the phosphonate library revealed pronounced in vitro cytostatic effect of the α-hydroxy- and α-mesyloxy-3,5-di-*tert*-butylbenzylphosphonates on human breast carcinoma cell culture with IC_50_ values of 16.4 and 28.0 μM, respectively. The mesyloxy species was also cytostatic on melanoma cells (IC_50_ = 34.9).

## 1 Introduction

The field of α-hydroxyphosphonates represents an evergreen topic, as their chemistry hides a lot of possibilities regarding synthesis and reactions. The principal method for the synthesis of α-hydroxyphosphonates is the Pudovik-reaction, according to which a dialkyl phosphite is added to the carbonyl group of aldehydes or ketones.^1^ It is also possible that the oxo compound is reacted with a trialkyl phosphite to afford eventually the corresponding hydroxyphosphonate.^2^ This addition may be catalyzed by bases or acids.^3−7^ Solvent-free methods on the surface of solid catalysts were also described,^8−10^ however, in most of the cases, a great amount of solvent had to be used during the workup. An indeed green protocol was when the hydroxyphosphonate crystallized out from acetone solution on the addition of pentane.^11^ The reversibility of the Pudovik reaction was also observed in certain cases.^12^ The α-hydroxyphosphonates may be involved in a series of reactions. A simple, but relatively less studied modification is alkylation.^1^ A much better-studied reaction is their acylation.^13−29^ This change in the functionality was especially useful in making available biologically active species.^13−16^ The phosphorylation of hydroxyphosphonates was elaborated by us.^30,31^ At first sight, it may be seen as surprising that the α-hydroxy-benzylphosphonates underwent substitution with primary amines on microwave (MW) irradiation.^32,33^ The reaction was promoted by an adjacent group effect. Additional transformations of α-hydroxyphosphonates include rearrangement,^34,35^ dealkylation,^1^ and oxidation and reduction.^1^ It was a further development to obtain hydroxyphosphonates in high enantiomeric excess following sequential asymmetric reactions.^36^

The α-hydroxyphosphonates and their derivatives are also of importance due to their many-sided biological activity. The antibacterial and antiviral activity of α-hydroxyphosphonates is in connection with their enzyme–inhibitory properties.^37−41^ A part of the acylated hydroxyphosphonates obtained on reaction with carboxylic acid chlorides or anhydrides included aryloxy-butyryloxy- or- valeroxy^13^ and heterocyclic derivatives^14,15^ that were described as herbicidal agents. Dialkyl α-hydroxy-benzylphosphonates and their acylated derivatives showed cytotoxic effects on certain cell lines.^16^ Phosphorylated species were active against the sarcoma cell line.^30^

It was a challenge for us to synthesize sulfonylated hydroxyphosphonates as newer derivatives and to explore their reactivity and cytotoxic activity.

## 2 Results and Discussion

### 2.1 Synthesis of Sulfonylated α-Hydroxy-benzylphosphonates

In the series of studying the reactivity of α-hydroxy-benzylphosphonates, the next task was to synthesize and investigate the properties of their sulfonylated derivatives. The starting α-hydroxyphosphonates (**2a**,**d–g** and **3a,c**–**h**) were prepared by the method described by us earlier.^11^ The two 3,5-di-*tert-*butylphenyl species (**2b** and **3b**) synthesized by our earlier procedure^11^ were new compounds and were fully characterized. The dimethyl and diethyl α-hydroxy-benzylphosphonates **(2a**,**b**,**d–f** and **3a**–**f**, respectively) were reacted with 1.5 equiv of methanesulfonyl chloride at 25 °C in the presence of 1.5 equiv of triethylamine in toluene for 0.5 h. The reaction involves a nucleophilic substitution on the SO_2_ moiety of the sulfonyl chloride by the hydroxyphosphonates (**2** and **3**). The workup included evaporation of the solvent and column chromatography of the residue so obtained. The expected mesyloxy-phosphonates **4a**,**b**,**d–f** and **5a**–**f** were isolated in 54–80% yields (Table 1, entries 1–11), and they were characterized by ^31^P, ^13^C, and ^1^H NMR spectral data, as well as HRMS. α-Hydroxy-benzylphosphonates **3a**,**d**,**e** were also reacted with *p*-toluenesulfonyl chloride under similar conditions to afford products **6a**,**d**,**e**, respectively, in 65–71% yields after the workup (Table 1, entries 12–14). As these products (**6a,d,e**) were described earlier,^42^ they were identified by ^31^P NMR spectral data, as well as HRMS (See Experimental Section).

**Table 1:**
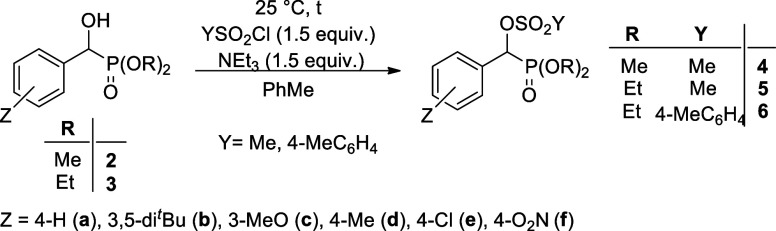
Synthesis of α-Methane- and *p*-Toluenesulfonyloxyphosphonates **4**–**6**

entry	starting material			*Y* of the sulfonyl chloride	*t* (h)	product	
		*Z*	*R*				yield (%)
1	**2a**	4-H	Me	Me	0.5	**4a**	67
2	**2b**	3,5-di^*t*^Bu	Me	Me	0.5	**4b**	60
3	**2d**	4-Me	Me	Me	0.5	**4d**	61
4	**2e**	4-Cl	Me	Me	0.5	**4e**	66
5	**2f**	4-O_2_N	Me	Me	0.5	**4f**	76
6	**3a**	4-H	Et	Me	0.5	**5a**	78
7	**3b**	3,5-di^*t*^Bu	Et	Me	0.5	**5b**	54
8	**3c**	3-MeO	Et	Me	0.5	**5c**	80
9	**3d**	4-Me	Et	Me	0.5	**5d**	76
10	**3e**	4-Cl	Et	Me	0.5	**5e**	79
11	**3f**	4-O_2_N	Et	Me	0.5	**5f**	76
12	**3a**	4-H	Et	4-MeC_6_H_4_	24	**6a**	65
13	**3d**	4-Me	Et	4-MeC_6_H_4_	24	**6d**	71
14	**3e**	4-Cl	Et	4-MeC_6_H_4_	24	**6e**	70

**Figure 1:**
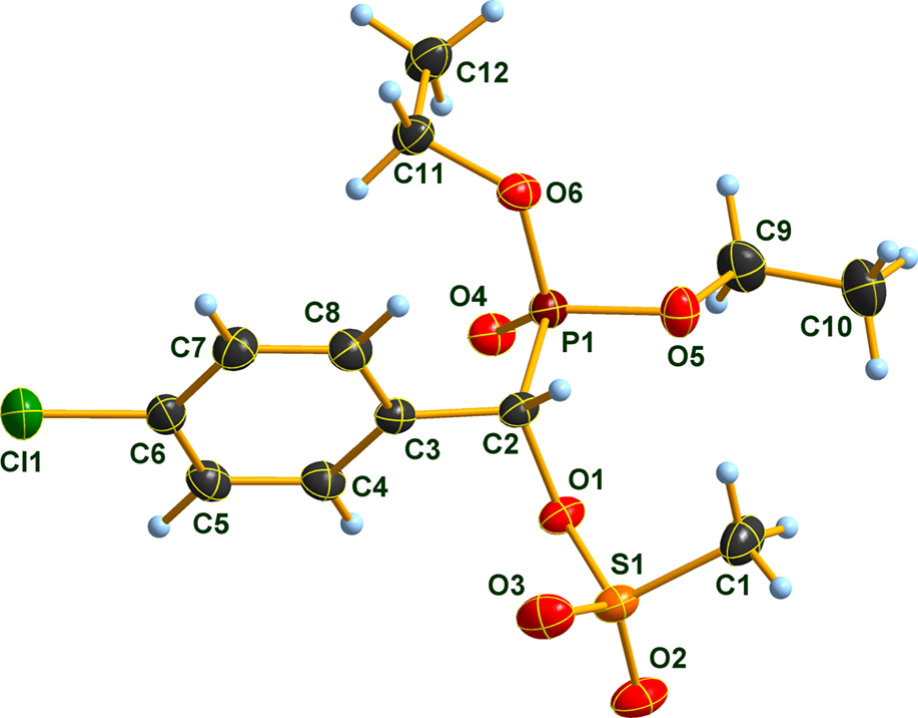
Molecular structure of compound **5e** in the crystal. DIAMOND^45^ representation thermal ellipsoids are drawn at 50% probability level.

Mesyloxy-phosphonate **5e** was also subjected to single-crystal X-ray diffraction studies. The compound crystallizes in the monoclinic space group *P*21/*n* with four formula units in the unit cell. The molecular structure of **5e** is shown in Figure 1, and views of the molecular packing in the crystal are displayed in SI Figures S1–S3. There are, to the best of our knowledge, only three molecules having a *P*-bonded phosphonate (P(O)(OR)_2_) unit and an *O*-bonded sulfonate (RSO_3_) unit, attached to the same tetrahedrally coordinated carbon atom, of which the molecular structures in the solid state have been determined.^43,44,29^ The first two cases^43,44^ refer to special molecules, structurally quite different from **5e**, and only compound **6a**(29) is available for a direct structural comparison. The structures of **6a** and **5e** are very similar in terms of bond lengths, angles, and molecular geometry. In both cases, phosphorus displays a slightly distorted tetrahedral surrounding and the P,C bond length (1.817(2) Å for **5e** and 1.822(3) Å for **6a**) are typical for a P,C single bond. The most interesting feature of both structures is the orientation of the *C*-bonded phenyl ring (torsion angle P–C–C-C 82.2(2)° for **5e** and 82.5(3)° for **6a**) tending to minimize steric repulsion by phosphonate and sulfonate substituents.

**Scheme 1:**
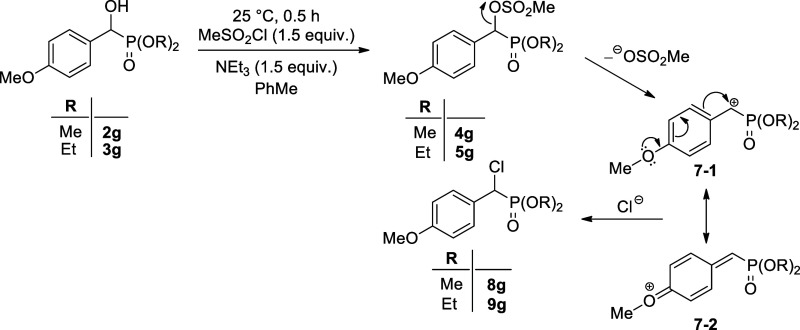
Reaction of Dialkyl α-Hydroxy-4-methoxybenzylphosphonates (**2g** and **3g**) with Methanesulfonyl Chloride

**Scheme 2:**
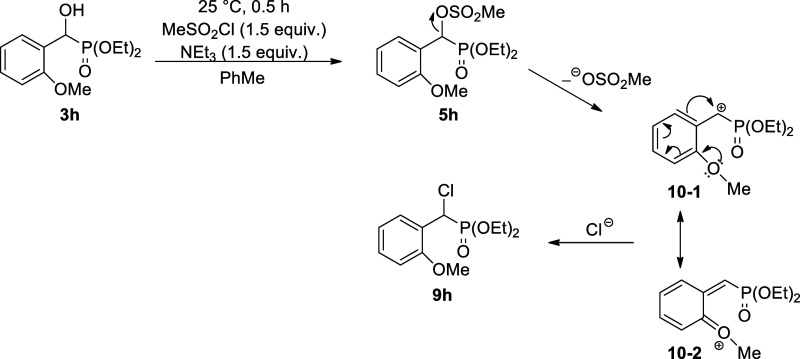
Reaction of Diethyl α-Hydroxy-2-methoxybenzylphosphonate **3h** with Methanesulfonyl Chloride

It was surprising to observe that the interaction of α-hydroxy-α-(4-methoxyphenyl)-methanephosphonates **2g** and **3g** led selectively to the corresponding **α-chloro-benzylphosphonates8g** and **9g**, respectively (Scheme 1). It is assumed that, in these cases, the expected mesyl derivatives (**4g** and **5g**) are only intermediates that give the chlorophosphonates (**8g** and **9g**) in reaction with the chloride anion deriving from the hydrochloric acid liberated. Departure of the MeSO_3_^–^ anion may lead to a cationic intermediate that may exist under two resonant forms (**7**–**1** and **7**–**2**), from among **7**–**2** is of a quinoid structure meaning a stabilization. The reaction of α-hydroxy-α-(2-methoxyphenyl)-methanephosphonate **3h** with methanesulfonyl chloride also gave the corresponding α-chloro-benzylphosphonate (**9h**) (Scheme 2). It can be said that the α-chlorophosphonates (**8g**, **9g**, and **9h**) are formed via an S_N_1 substitution.

**Scheme 3:**
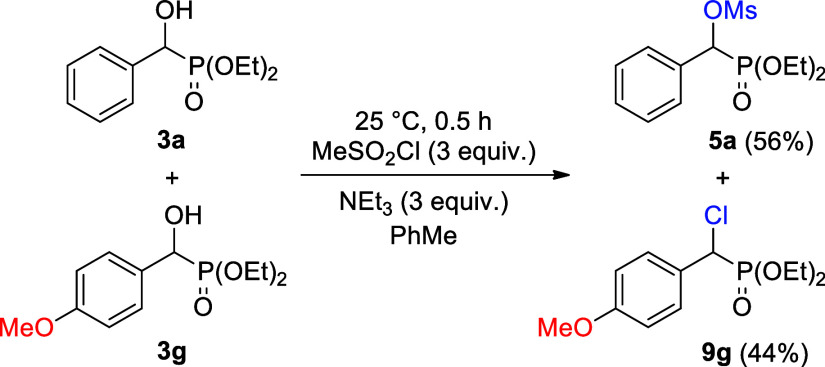
Competitive Reaction of Diethyl α-Hydroxy-benzylphosphonate (**3a**) and 4-Methoxybenzylphosphonate (**3g**) with Methanesulfonyl Chloride

To confirm this unexpected reactivity, an equimolar mixture of α-hydroxy-benzylphosphonate (**3a**) and 4-methoxy derivative (**3g**) was reacted with 3 equiv of the methanesulfonyl chloride at room temperature in toluene under the same conditions. ^31^P NMR spectrum of the crude mixture confirmed the presence of the mesyloxyphosphonate (**5a**) and **chlorophosphonates** (**9g**) in comparative quantities (Scheme 3).

### 2.2 Quantum Chemical Calculations

Notably, in the case of compounds **5a** and **5c**, no substitution of the mesyloxy group to chloro atom was observed, and the mesylated derivatives (**5a** and **5c**) were proved to be stable, contrary to mesylates **5g** and **5h** that were only intermediates and transformed in situ to chlorophosphonate **9g** and **9h**, respectively. These reactions were assumed to take place via an S_N_1 mechanism involving the triethylammonium-assisted cleavage of the C-OMs bond, where the driving force is surely the stability of the quinoidal carbocation **7**–**2** and **10**–**2**, respectively. The energies for the formation of intermediates **7** or **10** formed from diethyl α-mesyloxy-benzylphosphonate (**5a**), diethyl α-mesyloxy-3-methoxybenzylphosphonate (**5c**), diethyl α-mesyloxy-4-methoxybenzylphosphonate (**5g**), and diethyl α-mesyloxy-2-methoxybenzylphosphonate (**5h**) were computed. It was found that the transformation of phosphonates **5g** and **5h** to the corresponding carbocations (**7** and **10**, respectively) is an exothermic procedure, as suggested by the formation of Gibbs free energies of −9.0 and −14.1 kJ mol^–1^, respectively. On the contrary, and in accord with the experimental observations, the Gibbs free energies for the formation of the carbocations from **5a** and **5c** were found to be highly unfavorable, as marked by a Gibbs free energy of 49.1 and 53.2 kJ mol^–1^, respectively. This endothermic process is not supposed to occur at room temperature. The full details of the computations and the data set can be found in the Experimental Section and in the Supporting Information.

## 3 Reactions of the α-Mesyloxy-benzylphosphonates

### 3.1 Unexpected Substitution of **Methanesulfonyloxy-benzylphosphonates**

**Scheme 4:**
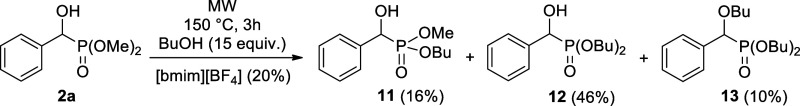
Alcoholysis of Dimethyl α-Hydroxy-benzylphosphonate **2a** with Butyl Alcohol

In the series of transesterification reactions investigated by us,^46,47^ dimethyl α-hydroxy-benzylphosphonate (**2a**) was rather reluctant to be involved in alcoholysis with butyl alcohol. Even under MW conditions at 150 °C using 20% of [bmim][BF_4_] as the catalyst, the conversion remained incomplete (72%), and a complex mixture comprising the butoxy-metoxy ester (**11**, δ_P_ (CDCl_3_) 22.5/22.6, [M + H]^+^ = 259), the dibutoxy ester (**12**, δ_P_ (CDCl_3_) 21.4, [M + H]^+^ = 301), and, surprisingly, the tributoxy product (**13**, δ_P_ (CDCl_3_) 19.1, [M + H]^+^ = 357) coming from O-alkylation of the fully transesterified product (**12**) was obtained (Scheme 4).

In the hope of a more selective α-substitution, we reacted mesyloxyphosphonates (**5a**, **5d**, and **5e**) with 15 equiv of alcohol in the presence of 1 equiv of triethylamine. The interaction of mesyloxyphosphonate **5a** with methyl alcohol at 135 °C for 2 h under MW irradiation led to the substitution of the α-mesyloxy group by a methoxy unit in a selective manner. No replacement of the ethoxy group(s) on the P atom to methoxy unit(s), i.e., no transesterification occurred. The α-methoxyphosphonate (**14a**) was formed in an S_N_2 nucleophilic substitution reaction in quantitative conversion (Table 2, entry 1). Then, this new and entirely selective reaction was extended also to other mesyloxyphosphonates. The 4-methylphenyl- and the 4-chlorophenyl model compounds (**5d** and **5e**, respectively) reacted in a similar way (Table 2, entries 2 and 3). To see the role of MW irradiation, the reaction of diethyl α-mesyloxy-benzylphosphonate (**5a**) with ethyl alcohol was performed first at 135 °C in a bomb. After heating for 2.5 h, the conversion was only 33%, however, a reaction time of 10 h led to an almost complete transformation (Table 2, entries 4 and 5). An exposure of 2.5 h on MW irradiation, led to a 96% conversion (Table 2, entry 6). Using pyridine instead of triethylamine under similar conditions was not advantageous, as a minor byproduct was also formed (Table 2, entry 7). The reaction of diethyl α-mesyloxy-α-(4-methylphenyl-)methylphosphonate (**5d**) took place in a similar way (Table 2, entry 8), however, the conversion of the 4-chlorophenyl derivative (**5e**) was reluctant (Table 2, entry 9), and there was need for 145 °C/3 h to reach an almost quantitative transformation (Table 2, entry 10). Changing for butyl alcohol, an irradiation of 2.5 h, or even 4 h at 135 °C was not enough, there was need for 4 h and 150 °C to reach a complete conversion (Table 2, entries 11–13). To avoid the minor decomposition, an irradiation at 145 °C for 4.5 h was the best option (Table 2, entry 14). The situation was similar for the reaction of the 4-methylphenyl derivative (**5d**) (Table 2, entry 15). The MsO → BuO substitution with the 4-chlorophenyl species (**5e**) was also complete after an irradiation time of 4.5 h at 145 °C (Table 2, entry 16). The α-alkoxy-benzylphosphonates (**14**-**16**) were obtained in 60–77% yields after purification by column chromatography from the best experiments. The α-alkoxyphosphonates (**14**-**16**) were characterized by ^31^P, ^13^C, and ^1^H NMR spectral data, as well as HRMS. Products **16a**,**d**,**e** are new compounds.

**Table 2:**
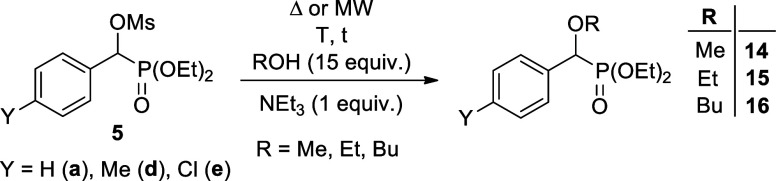
Conversion of Methanesulfonyloxy-benzylphosphonates (**5a**,**d**,**e**) to the Alkoxy Derivatives

entry	starting material	*Y*	*R* of the alcohol	heating source	*T* (°C)	*t* (h)	conversion (%)^a^	product	yield (%)
1	**5a**	H	Me	MW	135	2	100	**14a**	63
2	**5d**	Me	Me	MW	135	2	100	**14d**	77
3	**5e**	Cl	Me	MW	135	3	95	**14e**	60
4	**5a**	H	Et	Δ	135	2.5	33	**15a**	
5	**5a**	H	Et	Δ	135	10	97	**15a**	
6	**5a**	H	Et	MW	135	2.5	96	**15a**	75
7	**5a**	H	Et	MW	135	2.5	96^b^^,^^c^	**15a**	
8	**5d**	Me	Et	MW	135	2.5	100	**15d**	66
9	**5e**	Cl	Et	MW	135	2.5	55	**15e**	
10	**5e**	Cl	Et	MW	145	3	97	**15e**	74
11	**5a**	H	Bu	MW	135	2.5	28	**16a**	
12	**5a**	H	Bu	MW	135	4	70	**16a**	
13	**5a**	H	Bu	MW	150	4	100^d^	**16a**	
14	**5a**	H	Bu	MW	145	4.5	98	**16a**	62
15	**5d**	Me	Bu	MW	145	4.5	100	**16d**	70
16	**5e**	Cl	Bu	MW	145	4.5	100^d^	**16e**	61

a On the basis of relative ^31^P NMR intensities.

b Using pyridine instead of triethylamine.

c Unknown byproduct appeared at δ_P_ 16.8.

d Some decomposition occurred.

### 3.2 Novel Michaelis–Arbuzov Reaction of Methanesulfonyloxy-benzylphosphonates

It was a disappointing experience that the Michaelis–Arbuzov reaction of α-chloro- and even α-bromo-benzylphosphonates (**17a** and **17b**) with triethyl phosphite led to the desired bisphosphonic derivative (**18a**) with a low efficiency. Besides the unreacted triethyl phosphite, diethyl phosphite and diethyl ethylphosphonate were also present in the mixture. Using the **α-chlorophosphonate** (**17a**), the conversion was as low as 6% (Table 3, entry 1). The application of the α-bromophosphonate was more encouraging, however, the debromination of the starting substrate to benzylphosphonate **19** predominated (Table 3, entry 2). There are examples when triethylphosphite acted as a reducing agent.^48^

**Table 3:**
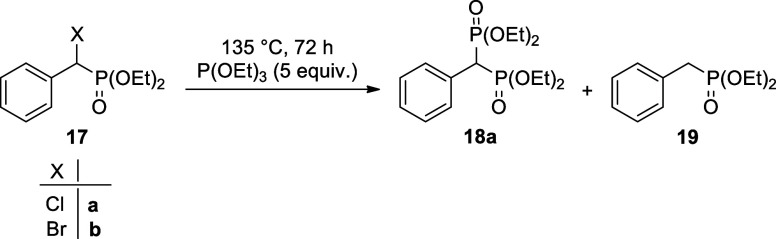
Attempted Michaelis–Arbuzov Reaction of α-Halogeno-benzylphosphonates (**17a** and **17b**) with Triethyl Phosphite

entry	*X*	starting material	conversion (%)^a^	composition of the mixture (%)^a^		
				18a	19	others
1	Cl	**17a**	∼6	5	<1	P(OEt)_3_, **(EtO)_2_P(O)H**, (EtO)_2_P(O)Et
2	Br	**17b**	98	45	53	(EtO)_2_P(O)H, (EtO)_2_P(O)Et

a On the basis ^31^P NMR relative intensities.

To the best of our knowledge, there is no mention in the literature about Michaelis–Arbuzov reactions applying an alkylmethanesulfonate ester instead of the halogeno reagents. Moreover, in one study, the mesyloxy group was replaced by an iodo atom before performing the Michaelis–Arbuzov reaction.^49^ However, a far analogy involving the reaction of thiosulfonates and phosphoramidites was described.^50^ We wished to try out the α-mesyloxy-benzylphosphonates (**5**) in Arbuzov-type fission. It was nice to find that the diethyl α-mesyloxy-benzylphosphonates (**5a**,**d**,**e**) could be involved in an efficient Michaelis–Arbuzov reaction using the triethyl phosphite in an excess at 135 °C, as shown in Table 4. Bisphosphonates **18a**,**d**,**e** were isolated in 76–81% yields after purification by column chromatography. Their structure was supported by ^31^P, ^13^C, and ^1^H NMR, as well as HRMS. Arylmethylenebisphosphonates **18a** and **18e** were described earlier,^51,52^ while compound **18d** is a new species.

**Table 4:**
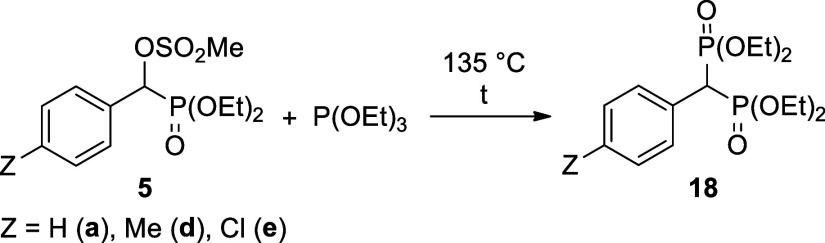
Michaelis–Arbuzov Reaction of α-Mesyloxy-benzylphosphonates (**5a**,**d**,**e**) with Triethyl Phosphite

entry	starting material	*Z*	P(OEt)_3_ (equiv)	*t* (day)	conversion (%)^a^	yield (%)
1	**5a**	H	5	3	83	
2	**5a**	H	9	4	92	80 (**18a**)
3	**5d**	Me	5	3	100	76 (**18d**)
4	**5e**	Cl	5	3	80	
5	**5e**	Cl	9	4	90	81 (**18e**)

a On the basis ^31^P NMR relative intensities.

## 4 In Vitro Cytostatic Activity of the Compounds Prepared

For the evaluation of the in vitro antiproliferative activity of the compounds, the cell viability was determined by resazurin (Alamar Blue) assay on MDA-MB 231 breast carcinoma and A2058 melanoma cell culture. The control wells were treated only with the serum-free medium. The IC_50_ values (the concentration which decreases the viability of the cells to 50% from the maximal viability) were determined from the dose–response curves and presented as micromolar (μM) units (data are summarized in Table 5). Compounds **3b** and **5b** induce cytostasis on MDA-MB231 cells with IC_50_ of 16.4 and 28.0 μM, respectively. The other phosphonates have no or only slight in vitro cytostatic effect on these cells. Compounds **2b**, **4b**, and **4f** revealed IC_50_ values of 95.4, 92.1, and 130.8 μM, respectively, on MDA-MB 231 cell culture. On the melanoma culture, phosphonate **5b** showed a fair antiproliferative effect (IC_50_ = 34.9 μM). Other compounds had no or limited inhibiting activity. Species **3b**, **4b**, and **4f** revealed IC_50_ values of 110.5, 106.0, and 119.9 μM, respectively, on A2058 cells. It is worthy to mention that diethyl α-hydroxy-di-*tert*-butylbenzylphosphonate **3b** showed a selective activity on one of the two cell cultures investigated. One may also see that the effect is somehow connected with the *tert*-butyl substituent in the phenyl ring. However, one can see that our best compounds (**3b** and **5b**) are less efficient than the reference compounds Daunomycin and Tamoxifen.

**Table 5:** In Vitro Cytostatic Effect of the Compounds, IC_50_ Values Presented as μM Units

IC_50_ (μM)		
cell culture		
compound	MDA-MB 231	A2058
**2b**	95.4	>250
**3b**	16.4	110.5
**4a**	>250	>250
**4b**	92.1	106.0
**4d**	>250	>250
**4e**	>250	>250
**4f**	130.8	119.9
**5a**	>250	>250
**5b**	28.0	34.9
**5c**	>250	>250
**5d**	>250	>250
**5e**	>250	>250
**5f**	>250	>250
**7a**	>250	>250
**7e**	>250	>250
**14a**	>250	>250
**14d**	>250	>250
**14e**	>250	>250
**15a**	>250	>250
**15d**	>250	>250
**15e**	>250	>250
**16a**	>250	109.8
**16d**	>250	>250
**16e**	91.6	114.5
**18a**	>250	>250
**18d**	>250	>250
**18e**	>250	>250
daunomycin	0.7	0.9
tamoxifen	3.4	1.0

## 5 Conclusions

A new family of compounds, methane- and arenesulfonyloxy-benzylphosphonates were prepared by the reaction of α-hydroxy-benzylphosphonates and the corresponding sulfonyl chloride. When the starting hydroxyphosphonate bore a methoxy substituent in position 4 or 2 in the phenyl ring, the respective chlorophosphonate was the only product, whose formation could be explained by the intermediacy of a stabilized quinoid species, as confirmed by quantum chemical calculations. The reactivity of the methanesulfonyloxy-benzylphosphonates was explored in reaction with alcohols and in the Michaelis–Arbuzov reaction. In the first case, on MW irradiation at ∼140 °C, in the presence of triethylamine, not alcoholysis, but a selective nucleophilic substitution on the α-carbon atom to provide the α-alkoxy derivatives took place. In the second case, an efficient Michaelis–Arbuzov fission occurred, which is a novel and valuable experience, as the similar reaction of the related α-halogeno phosphonates is insufficient. Among the phosphonate derivatives, the α-hydroxy- and α-mesyloxy-3,5-di-*tert*-butylbenzylphosphonates showed significant cytostatic effect on the MDA-MB 231 human breast carcinoma cell line, and the mesyloxy derivative also on A2058 melanoma cell culture.

## 6 Experimental Section

### 6.1 General Information

The MW reactions were carried out in a CEM Discover (300 W) focused MW reactor (CEM Microwave Technology Ltd., Buckingham, U.K.) equipped with a stirrer and a pressure controller using 80–100 W irradiation under isothermal conditions. The reaction mixtures were irradiated in sealed glass vessels (with a volume of 10 mL) available from the supplier of CEM. The reaction temperature was monitored by an external IR sensor.

The ^31^P, ^13^C, ^1^H NMR spectra were taken on a Bruker DRX-500 or Bruker Avance-300 spectrometer operating at 202, 126, and 500 MHz or 122, 75, and 300 MHz, respectively. The couplings were given in Hertz. LC–MS measurements were performed with an Agilent 1200 liquid chromatography system, coupled with a 6130 quadrupole mass spectrometer equipped with an ESI ion source (Agilent Technologies, Palo Alto, CA, USA). High-resolution mass spectrometric measurements were performed using a Thermo Velos Pro Orbitrap Elite hybrid mass spectrometer in positive electrospray mode.

### 6.2 General Procedure for the Synthesis of Dialkyl α-Hydroxy-benzylphosphonates

11.0 mmol of aromatic aldehyde (benzaldehyde 1.2 g, *p*-chlorobenzaldehyde 1.5 g, *p*-nitrobenzaldehyde 1.7 g, *p*-methylbenzaldehyde 1.3 g, *p*-methoxybenzaldehyde 1.5 g, *m*-methoxybenzaldehyde 1.5 g, *o*-methoxybenzaldehyde 1.5 g, 3,5-di-*tert*-butyl-benzaldehyde 2.4 g) and 11.0 mmol of dialkyl phosphite (dimethyl phosphite 1.1 mL, diethyl phosphite 1.4 mL) and 1.1 mmol (0.15 mL) of triethylamine were stirred in 1 mL acetone at reflux. After 1–6 h, 6 mL of *n*-pentane was added to the reaction mixture, and it was cooled to 5 °C. Compounds **2a**,**b**,**d–g** and **3a**,**b**,**d–g** crystallized from the reaction mixture. The crystals were filtered off and washed with *n*-pentane. In two cases, the products (**3c**,**h**) were purified by column chromatography (using DCM–MeOH 97:3 as the eluent on silica gel). Products **2a,b**,**d**,**e**,**g** and **3a,b**,**d**,**e**,**g** are white, hydroxyphosphonates **2f** and **3f** are yellow crystalline compounds, **3c** and **3h** are colorless oils. The exact conditions are shown in Table 6.

**Table 6:**
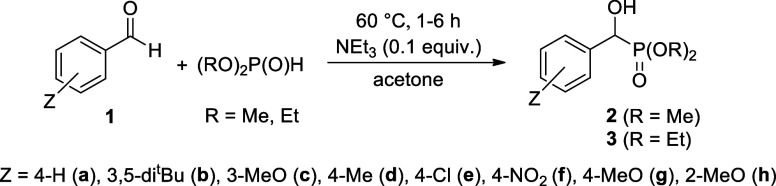
Preparation of the Starting α-Hydroxy-benzylphosphonates **2** and **3**

Z	*R*	*t* (h)	product	yield (%)	δ ^31^P (CDCl_3_)	δ ^31^P (CDCl_3_) ^lit.^	[M + H]^+^	mp (°C)	mp (°C) ^lit.^
H^11^	Me	1	**2a**	77	23.6	23.8	217	100–101	101–102^53^
3,5-di^*t*^Bu	Me	6	**2b**	64	24.0		329	130–131	
4-Me^11^	Me	1.5	**2d**	88	24.1	24.1	231	99–100	98^54^
4-Cl^11^	Me	1	**2e**	80	22.9	23.2	251	101–102	104–105^55^
4-NO_2_^11^	Me	1.5	**2f**	77	21.9	22.3	262	129–130	129–131^56^
4-MeO^57^	Me	2	**2g**	85	23.4	23.4	247	73–74	72^54^
H^11^	Et	1	**3a**	87	21.4	21.7	245	83–84	83–84^58^
3,5-di^*t*^Bu	Et	6	**3b**	78	21.8		357	84–85	
3-MeO^59^	Et	2	**3c**	87	21.4	25.2	275		
4-Me^57^	Et	3	**3d**	71	21.6	21.7	259	96–97	94–95^60^
4-Cl^11^	Et	2	**3e**	74	20.9	21.0	279	74–75	73–74^61^
4-NO_2_^11^	Et	1.5	**3f**	75	19.8	20.0	290	89–90	90–91^62^
4-MeO^57^	Et	2	**3g**	76	21.7	21.5	275	121–122	120–121.5^60^
2-MeO^59^	Et	3	**3h**	77	22.3	26.3	275		

#### 6.2.1 Dimethyl α-Hydroxy-3,5-di-*tert*-butylbenzylphosphonate (**2b**)

Yield: 2.3 g (64%); white solid; m.p.: 130–131 °C; ^31^P {^1^H} NMR (202 MHz, CDCl_3_) δ 24.0; ^13^C {^1^H} NMR (126 MHz, CDCl_3_) δ 31.5 (s, 6 C*C*H_3_), 34.9 (s, 2 *C*CH_3_), 53.6 and 53.9 (d, *J* = 7.3 Hz, 2 OCH_3_), 71.2 (d, *J* = 158.5 Hz, CH), 121.5 (d, *J* = 5.9 Hz, C_β_), 122.0 (bs, C_δ_), 135.4 (s, C_α_), 150.7 (s, C_γ_); ^1^H NMR (500 MHz, CDCl_3_) δ 1.35 (s, 18H, CCH_3_), 3.64 and 3.73 (d, *J* = 10.5 Hz, 6H, OCH_3_), 5.06 (dd, *J*_1_ = 10.6 Hz, *J*_2_ = 5.0 Hz, 1H, CH), 7.34–7.35 and 7.40–7.41 (m, 3H, ArH); [M + H]^+^ = 329; HRMS *m*/*z*: [M + Na]^+^ calculated for C_17_H_29_O_4_PNa 351.1701; found 351.1697.

#### 6.2.2 Diethyl α-Hydroxy-3,5-di-*tert*-butylbenzylphosphonate (**3b**)

Yield: 3.1 g (78%); white solid; m.p.: 84–85 °C; ^31^P {^1^H} NMR (202 MHz, CDCl_3_) δ 21.8; ^13^C {^1^H} NMR (126 MHz, CDCl_3_) δ 16.4 (t, *J* = 6.2 Hz, 2 CH_2_*C*H_3_), 31.5 (s, 6 C*C*H_3_), 34.9 (2 *C*CH_3_), 62.8 and 63.2 (d, *J* = 7.2 Hz, 2 OCH_2_), 71.4 (d, *J* = 158.7 Hz, CH), 121.5 (d, *J* = 5.9 Hz, C_β_), 121.9 (bs, C_δ_), 135.6 (s, C_α_), 150.5 (s, C_γ_); ^1^H NMR (500 MHz, CDCl_3_) δ 1.21 and 1.29 (t, *J* = 6.9 Hz, 6H, CH_2_C*H*_*3*_), 1.35 (s, 18H, CCH_3_), 3.93–4.10 (m, 4H, OCH_2_), 5.03 (d, *J* = 1.3 Hz, 1H, CH), 7.34–7.39 (m, 3H, ArH); [M + H]^+^ = 357; HRMS *m*/*z*: [M + Na]^+^ calculated for C_19_H_33_O_4_PNa 379.2014; found 379.2007.

### 6.3 General Procedure for the Synthesis of Dialkyl α-Methanesulfonyloxy-arylphosphonates

1.0 mmol of dialkyl α-hydroxy-benzylphosphonate (**2a**: 0.22 g; **3a**: 0.24 g; **2b**: 0.33 g; **3b**: 0.36 g; **3c**: 0.27 g; **2d**: 0.23 g; **3d**: 0.26 g; **2e**: 0.25 g, **3e**: 0.28 g, **2f**: 0.26 g, **3f**: 0.29 g), 1.5 mmol (0.12 mL) of methanesulfonyl chloride and 1.5 mmol (0.21 mL) of triethylamine in 5 mL of toluene were mixed at room temperature for half an hour. The precipitated triethylamine hydrochloride salt was filtered off, the filtrate was evaporated under vacuum, and the crude product was purified by column chromatography (using DCM–MeOH 95:5 as the eluent on silica gel).

#### 6.3.1 Dimethyl α-Methanesulfonyloxy-benzylphosphonate (**4a**)

Yield: 0.20 g (67%); white solid; m.p.: 105–106 °C; ^31^P {^1^H} NMR (202 MHz, CDCl_3_) δ 16.9; ^13^C {^1^H} NMR (126 MHz, CDCl_3_) δ 39.5 (s, SCH_3_), 54.1 and 54.5 (d, *J* = 7.1 Hz, 2 OCH_3_), 77.0 (d, *J* = 171.4 Hz, CH), 128.1 (d, *J* = 5.9 Hz, C_β_), 129.0 (d, *J* = 2.0 Hz, C_γ_), 129.8 (d, *J* = 2.7 Hz, C_δ_), 131.7 (bs, C_α_); ^1^H NMR (500 MHz, CDCl_3_) δ 2.87 (s, 3H, SCH_3_), 3.70 and 3.84 (d, *J* = 10.7 Hz, 6H, OCH_3_), 5.79 (d, *J* = 15.1 Hz, 1H, CH), 7.43–7.57 (m, 5H, ArH), [M + H]^+^ = 295; HRMS *m*/*z*: [M + Na]^+^ calculated for C_10_H_15_O_6_PSNa 317.0225; found 317.0222.

#### 6.3.2 Dimethyl α-Methanesulfonyloxy-3,5-di-*tert*-butylbenzylphosphonate (**4b**)

Yield: 0.24 g (60%); colorless oil; ^31^P {^1^H} NMR (202 MHz, CDCl_3_) δ 17.3; ^13^C {^1^H} NMR (126 MHz, CDCl_3_) δ 31.4 (s, 6 C*C*H_3_), 35.0 (s, 2 *C*CH_3_), 39.7 (s, SCH_3_), 54.1 and 54.4 (d, *J* = 6.9 Hz, 2 OCH_3_), 77.8 (d, *J* = 170.3 Hz, CH), 122.7 (d, *J* = 6.0 Hz, C_β_), 123.7 (d, *J* = 2.6 Hz, C_δ_), 130.6 (s, C_α_), 151.6 (d, *J* = 2.0 Hz, C_γ_); ^1^H NMR (500 MHz, CDCl_3_) δ 1.33 (s, 18H, CCH_3_), 2.79 (s, 3H, SCH_3_), 3.65 and 3.80 (d, *J* = 10.7 Hz, 6H, OCH_3_), 5.77 (d, *J* = 14.7 Hz, 1H, CH), 7.36–7.37 and 7.44–7.46 (m, 3H, ArH); [M + H]^+^ = 407; HRMS *m*/*z* [M + Na]^+^ calculated for C_18_H_31_O_6_PSNa 429.1477; found 429.1477.

#### 6.3.3 Dimethyl α-Methanesulfonyloxy-4-methylbenzylphosphonate (**4d**)

Yield: 0.20 g (61%); white solid; m.p.: 85–86 °C; ^31^P {^1^H} NMR (202 MHz, CDCl_3_) δ 17.1; ^13^C {^1^H} NMR (126 MHz, CDCl_3_) δ 21.3 (s, ArCH_3_), 39.7 (s, SCH_3_), 54.1 and 54.5 (d, *J* = 6.8 Hz, 2 OCH_3_), 77.2 (d, *J* = 172.9 Hz, CH), 128.3 (d, *J* = 6.1 Hz, C_β_), 128.6 (d, *J* = 1.7 Hz, C_α_), 129.7 (d, *J* = 2.0 Hz, C_γ_), 140.1 (d, *J* = 2.7 Hz, C_δ_); ^1^H NMR (500 MHz, CDCl_3_) δ 2.39 (s, 3H, ArCH_3_), 2.84 (s, 3H, SCH_3_), 3.70 and 3.86 (d, *J* = 10.7 Hz, 6H, OCH_3_), 5.76 (d, *J* = 14.9 Hz, 1H, CH), 7.25–7.26 and 7.45–7.46 (m, 4H, ArH); [M + H]^+^ = 309; HRMS *m*/*z*: [M + Na]^+^ calculated for C_11_H_17_O_6_PSNa 331.0381; found 331.0376.

#### 6.3.4 Dimethyl α-Methanesulfonyloxy-4-chlorobenzylphosphonate (**4e**)

Yield: 0.22 g (66%); white solid; m.p.: 120–121 °C; ^31^P {^1^H} NMR (202 MHz, CDCl_3_) δ 16.6; ^13^C {^1^H} NMR (126 MHz, CDCl_3_) δ 39.6 (s, SCH_3_), 54.2 and 54.6 (d, *J* = 6.7 Hz, 2 OCH_3_), 76.1 (d, *J* = 172.6 Hz, CH), 129.3 (d, *J* = 1.9 Hz, C_γ_), 129.5 (d, *J* = 6.1 Hz, C_β_), 130.4 (d, *J* = 1.8 Hz, C_α_), 135.9 (d, *J* = 3.2 Hz, C_δ_); ^1^H NMR (500 MHz, CDCl_3_) δ 2.94 (s, 3H, SCH_3_), 3.74 and 3.85 (d, *J* = 10.7 Hz, 6H, OCH_3_), 5.77 (d, *J* = 15.1 Hz, 1H, CH), 7.43–7.52 (m, 4H, ArH), [M + H]^+^ = 329; HRMS *m*/*z*: [M + Na]^+^ calculated for C_10_H_14_ClO_6_PSNa 350.9835; found 350.9831.

#### 6.3.5 Dimethyl α-Methanesulfonyloxy-4-nitrobenzylphosphonate (**4f**)

Yield: 0.26 g (76%); pale yellow solid; m.p.: 163–164 °C; ^31^P {^1^H} NMR (202 MHz, CDCl_3_) δ 15.8; ^13^C {^1^H} NMR (75 MHz, CDCl_3_) δ 39.4 (s, SCH_3_), 54.4 and 54.7 (d, *J* = 6.8 Hz, 2 OCH_3_), 75.3 (d, *J* = 169.1 Hz, CH), 124.0 (d, *J* = 2.3 Hz, C_γ_), 128.6 (d, *J* = 5.3 Hz, C_β_), 139.2 (d, *J* = 1.9 Hz, C_α_), 148.5 (bs, C_δ_); ^1^H NMR (500 MHz, CDCl_3_) δ 3.09 (s, 3H, SCH_3_), 3.81 and 3.85 (d, *J* = 10.8 Hz, 6H, OCH_3_), 5.90 (d, *J* = 15.9 Hz, 1H, CH), 7.72–7.74 and 8.30–8.32 (m, 4H, ArH); [M + H]^+^ = 340; HRMS *m*/*z*: [M + Na]^+^ calculated for C_10_H_14_NO_8_PSNa 362.0075; found 362.0072.

#### 6.3.6 Diethyl α-Methanesulfonyloxy-benzylphosphonate (**5a**)

Yield: 0.25 g (78%); white solid; m.p.: 72–73 °C; ^31^P {^1^H} NMR (202 MHz, CDCl_3_) δ 14.5; ^13^C {^1^H} NMR (126 MHz, CDCl_3_) δ 16.2 and 16.4 (d, *J* = 5.7 Hz, 2 CH_2_*C*H_3_), 39.6 (s, SCH_3_), 63.8 and 64.1 (d, *J* = 6.9 Hz, 2 OCH_2_), 77.5 (d, *J* = 171.2 Hz, CH), 128.2 (d, *J* = 5.9 Hz, C_β_), 128.8 (d, *J* = 1.8 Hz, C_γ_), 129.7 (d, *J* = 2.6 Hz, C_δ_), 131.9 (d, J = 1.6 Hz, C_α_); ^1^H NMR (500 MHz, CDC1_3_) δ 1.24 and 1.35 (t, *J* = 7.1 Hz, 6H, CH_2_C*H*_*3*_), 2.86 (s, 3H, SCH_3_), 3.95–4.23 (m, 4H, OCH_2_), 5.76 (d, *J* = 15.1 Hz, 1H, CH), 7.41–7.58 (m, 5H, ArH), δ lit.^63^ 1.20 and 1.30 (t, 6H, *J* = 7.0 Hz, CH_2_C*H*_*3*_), 2.82 (s, 3H, SCH_3_), 3.70–4.40 (m, 4H, OCH_2_), 5.70 (d, 1H, *J* = 15.0 Hz, CH), 7.20–7.60 (m, 5H, ArH); [M + H]^+^ = 323; HRMS *m*/*z*: [M + Na]^+^ calculated for C_12_H_19_O_6_PSNa 345.0538; found 345.0536.

#### 6.3.7 Diethyl α-Methanesulfonyloxy-3,5-di-*tert*-butylbenzylphosphonate (**5b**)

Yield: 0.24 g (54%); colorless oil; ^31^P {^1^H} NMR (202 MHz, CDCl_3_) δ 14.9; ^13^C {^1^H} NMR (126 MHz, CDCl_3_) δ 16.2 and 16.4 (d, *J* = 5.9 Hz, 2 CH_2_*C*H_3_), 31.4 (s, 6 C*C*H_3_), 34.9 (s, 2 *C*CH_3_), 39.7 (s, SCH_3_), 63.7 and 63.9 (d, *J* = 6.5 Hz, 2 OCH_2_), 78.5 (d, *J* = 171.1 Hz, CH), 122.7 (d, *J* = 6.0 Hz, C_β_), 123.5 (d, *J* = 2.6 Hz, C_δ_), 130.8 (s, C_α_), 151.4 (d, *J* = 1.9 Hz, C_γ_); ^1^H NMR (500 MHz, CDCl_3_) δ 1.21 and 1.35 (t, *J* = 7.1 Hz, 6H, CH_2_C*H*_*3*_), 1.35 (s, 18H, CCH_3_), 2.81 (s, 3H, SCH_3_), 3.93–4.21 (m, 8H, OCH_2_), 5.76 (d, *J* = 14.7 Hz, 1H, CH), 7.37–7.46 (m, 3H, ArH); [M + H]^+^ = 437; HRMS *m*/*z*: [M + Na]^+^ calculated for C_20_H_35_O_6_PSNa 457.1790; found 457.1776.

#### 6.3.8 Diethyl α-Methanesulfonyloxy-3-methyoxybenzylphosphonate (**5c**)

Yield: 0.28 g (80%), pale yellow oil; ^31^P {^1^H} NMR (202 MHz, CDCl_3_) δ 14.5; ^13^C {^1^H} NMR (126 MHz, CDCl_3_) δ 16.3 and 16.4 (d, *J* = 5.8 Hz, 2 CH_2_*C*H_3_), 39.6 (s, SCH_3_), 55.4 (s, OCH_3_), 63.8 and 64.2 (d, *J* = 6.9 Hz, 2 OCH_2_), 77.5 (d, *J* = 171.2 Hz, CH), 113.5 (d, *J* = 5.7 Hz, C_6_*), 115.6 (d, *J* = 2.6 Hz, C_5_*) 120.5 (d, *J* = 6.0 Hz, C_2_*), 129.9 (d, *J* = 1.9 Hz, C_4_), 133.3 (d, *J* = 1.7 Hz, C_1_) 159.8 (s, C_3_(OMe)), *tentative; ^1^H NMR (500 MHz, CDCl_3_) δ 1.17 and 1.26 (t, *J* = 7.1 Hz, 6H, CH_2_C*H*_*3*_), 2.79 (s, 3H, SCH_3_), 3.76 (s, 3H, OCH_3_), 3.87–4.14 (m, 4H, OCH_2_), 5.63 (d, *J* = 15.1 Hz, 1H, CH), 6.86–6.87, 7.01–7.04 and 7.23–7.27 (m, 4H, ArH); [M + H]^+^ = 353; HRMS *m*/*z*: [M + Na]^+^ calculated for C_13_H_21_O_7_PSNa 375.0643; found 375.0642.

#### 6.3.9 Diethyl α-Methanesulfonyloxy-4-methylbenzylphosphonate (**5d**)

Yield: 0.26 g (76%), colorless oil; ^31^P {^1^H} NMR (202 MHz, CDCl_3_) δ 14.7; ^13^C {^1^H} NMR (126 MHz, CDCl_3_) δ 16.3 and 16.4 (d, *J* = 5.7 Hz, 2 CH_2_*C*H_3_), 21.3 (s, ArCH_3_), 39.7 (s, SCH_3_), 63.7 and 64.1 (d, *J* = 6.7 Hz, 2 OCH_2_), 77.6 (d, *J* = 172.4 Hz, CH), 128.3 (d, *J* = 5.9 Hz, C_β_), 128.8 (d, *J* = 1.6 Hz, C_α_), 129.6 (d, *J* = 1.9 Hz, C_γ_), 139.8 (d, *J* = 2.8 Hz, C_δ_); ^1^H NMR (500 MHz, CDCl_3_) δ 1.24 and 1.36 (t, *J* = 7.1 Hz, 6H, CH_2_C*H*_*3*_), 2.39 (s, 3H, ArCH_3_) 2.83 (s, 3H, SCH_3_), 3.94–4.23 (m, 4H, OCH_2_), 5.72 (d, *J* = 14.9 Hz, 1H, CH), 7.23–7.25 and 7.45–7.46 (m, 4H, ArH); [M + H]^+^ = 337; HRMS *m*/*z* [M + Na]^+^ calculated for C_13_H_21_O_6_PSNa 359.0694; found 359.0694.

#### 6.3.10 Diethyl α-Methanesulfonyloxy-4-chlorobenzylphosphonate (**5e**)

Yield: 0.28 g (79%); white crystals; m.p.: 76–77 °C; ^31^P {^1^H} NMR (202 MHz, CDCl_3_) δ 14.2; ^13^C {^1^H} NMR (126 MHz, CDCl_3_) δ 16.2 and 16.3 (d, *J* = 5.7 Hz, 2 CH_2_*C*H_3_), 39.5 (s, SCH_3_), 63.9 and 64.2 (d, *J* = 6.8 Hz, 2 OCH_2_), 76.5 (d, *J* = 171.6 Hz, CH), 129.0 (d, *J* = 2.0 Hz, C_γ_), 129.5 (d, *J* = 5.7 Hz, C_β_), 130.7 (bs, C_α_), 135.6 (bs, C_δ_); ^1^H NMR (500 MHz, CDCl_3_) δ 1.27 and 1.35 (t, *J* = 7.0 Hz, 6H, CH_2_C*H*_*3*_), 2.93 (s, 3H, SCH_3_), 3.99–4.23 (m, 4H, OCH_2_), 5.73 (d, *J* = 15.1 Hz, 1H, CH), 7.41–7.51 (m, 4H, ArH); [M + H]^+^ = 357; HRMS *m*/*z* [M + Na]^+^ calculated for C_12_H_18_ClO_6_PSNa 379.0148; found 379.0143.

#### 6.3.11 Diethyl α-Methanesulfonyloxy-4-nitrobenzylphosphonate (**5f**)

Yield: 0.28 g (76%); yellow oil; ^31^P {^1^H} NMR (202 MHz, CDCl_3_) δ 13.3; ^13^C {^1^H} NMR (75 MHz, CDCl_3_) δ 16.3 and 16.4 (d, *J* = 5.7 Hz, 2 CH_2_*C*H_3_), 39.4 (s, SCH_3_), 64.2 and 64.5 (d, *J* = 6.8 Hz, 2 OCH_2_), 75.8 (d, *J* = 168.4 Hz, CH), 123.8 (d, *J* = 2.2 Hz, C_γ_), 128.6 (d, *J* = 5.2 Hz, C_β_), 139.6 (d, *J* = 1.9 Hz, C_α_), 148.3 (d, *J* = 3.1 Hz, C_δ_); ^1^H NMR (500 MHz, CDCl_3_) δ 1.31 and 1.34 (t, *J* = 7.1 Hz, 6H, CH_2_C*H*_*3*_), 3.09 (s, 3H, SCH_3_), 4.10–4.21 (m, 4H, OCH_2_), 5.87 (d, *J* = 15.9 Hz, 1H, CH), 7.71–7.73 and 8.29–8.30 (m, 4H, ArH); [M + H]^+^ = 368; HRMS *m*/*z* [M + Na]^+^ calculated for C_12_H_18_NO_8_PSNa 390.0388; found 390.0390.

### 6.4 General Procedure for the Synthesis of Dialkyl α-Toluenesulfonyloxy-arylphosphonates

1.0 mmol of dialkyl α-hydroxy-benzylphosphonate (**3a**: 0.24 g; **3d**: 0.26 g; **3e**: 0.28 g), 1.5 mmol (0.29 g) of *p*-toluenesulfonyl chloride and 1.5 mmol (0.21 mL) of triethylamine in 5 mL of toluene were mixed at room temperature for 24 h. The precipitated salt was filtered off, the filtrate evaporated under vacuum, and the crude product purified by column chromatography (using DCM–MeOH 97:3 as the eluent on silica gel).

#### 6.4.1 Diethyl α-4-Methylbenzenesulfonyloxy-benzylphosphonate (**6a**)

Yield: 0.26 g (65%); white solid; m.p.: 63–64 °C, mp lit.:^29^ 63–65 °C; ^31^P {^1^H} NMR (202 MHz, CDCl_3_) δ 14.6, δ_P_ lit.^42^ 14.6; [M + H]^+^ = 399; HRMS *m*/*z*: [M + Na]^+^ calculated for C_18_H_23_O_6_PSNa 421.0851; found 421.0857.

#### 6.4.2 Diethyl α-4-Methylbenzenesulfonyloxy-4-methylbenzylphosphonate (**6d**)

Yield: 0.29 g (71%); colorless oil; ^31^P {^1^H} NMR (202 MHz, CDCl_3_) δ 14.7, δ_P_ lit.^42^ 14.8; [M + H]^+^ = 413; HRMS *m*/*z*: [M + Na]^+^ calculated for C_19_H_25_O_6_PSNa 435.1007; found 435.1006.

#### 6.4.3 Diethyl α-4-Methylbenzenesulfonyloxy-4-chlorobenzylphosphonate (**6e**)

Yield: 0.30 g (70%); colorless oil; ^31^P {^1^H} NMR (202 MHz, CDCl_3_) δ 14.1, δ_P_ lit.^42^ 14.1; [M + H]^+^ = 433; HRMS *m*/*z*: [M + Na]^+^ calculated for C_18_H_22_ClO_6_PSNa 455.0461; found 455.0460.

### 6.5 General Procedure for the Synthesis of Diethyl α-Chloro-arylphosphonates

1.0 mmol of dialkyl **α-hydroxy-benzylphosphonate** (**2g**: 0.25 g; **3g**: 0.27 g; **3h**: 0.27 g), 1.5 mmol (0.12 mL) of methanesulfonyl chloride and 1.5 mmol (0.21 mL) of triethylamine in 5 mL of toluene were mixed at room temperature for half an hour. The precipitated salt was filtered off, the filtrate evaporated under vacuum, and the crude product purified by column chromatography (using DCM–MeOH 95:5 as the eluent on silica gel).

#### 6.5.1 Dimethyl α-Chloro-4-methyoxybenzylphosphonate (**8g**)

Yield: 0.18 g (68%); pale yellow oil; ^31^P {^1^H} NMR (202 MHz, CDCl_3_) δ 19.7; ^13^C {^1^H} NMR (126 MHz, CDCl_3_) δ 53.0 (d, *J* = 163.1 Hz, CH), 54.3 and 54.6 (d, *J* = 6.9 Hz, 2 OCH_3_), 55.3 (s, OCH_3_), 114.1 (d, *J* = 1.7 Hz, C_γ_), 125.8 (d, *J* = 3.6 Hz, C_α_), 130.3 (d, *J* = 6.5 Hz, C_β_), 160.2 (d, *J* = 2.3 Hz, C_δ_); ^1^H NMR (500 MHz, CDCl_3_) δ 3.63 and 3.85 (d, *J* = 10.7 Hz, 6H, OCH_3_), 3.82 (s, 3H, OCH_3_), 4.90 (d, *J* = 13.8 Hz, 1H, CH), 6.91 (d, *J* = 8.7 Hz, 2H, ArH), 7.47 (dd, *J*_1_ = 8.8 Hz, *J*_2_ = 1.9 Hz, 2H, ArH), δ lit.^64^ 3.60 and 3.81 (d, *J* = 10.0 Hz, 6H, OCH_3_), 3.79 (s, 3H, OCH_3_), 4.90 (d, *J* = 14.0 Hz, 1H, CH), 6.90 (d, *J* = 8 Hz, 2H, ArH), 7.50 (dd, *J*_1_ = 8 Hz, *J*_2_ = 2 Hz, 2H, ArH); [M + H]^+^ = 265; HRMS *m*/*z*: [M + Na]^+^ calculated for C_10_H_14_ClO_4_PNa 287.0216; found 287.0210.

#### 6.5.2 Diethyl α-Chloro-4-methyoxybenzylphosphonate (**9g**)

Yield: 0.20 g (70%); pale yellow oil; ^31^P {^1^H} NMR (202 MHz, CDCl_3_) δ 17.4, δ_P_ lit.^65^ 17.6; [M + H]^+^ = 293; HRMS *m*/*z* [M + Na]^+^ calculated for C_12_H_18_ClO_4_PNa 315.0529; found 315.0528.

#### 6.5.3 Diethyl α-Chloro-2-methyoxybenzyl]phosphonate (**9h**)

Yield: 0.18 g (65%); pale yellow oil; ^31^P {^1^H} NMR (202 MHz, CDCl_3_) δ 18.1; ^13^C {^1^H} NMR (126 MHz, CDCl_3_) δ 16.2 and 16.4 (d, *J* = 5.9 Hz, 2 CH_2_*C*H_3_), 45.6 (d, *J* = 163.6 Hz, CH), 55.7 (s, OCH_3_), 63.7 and 63.8 (d, J = 7.0 Hz, 2 OCH_2_), 110.5 (d, *J* = 1.4 Hz, C_5_), 121.0 (d, *J* = 2.3 Hz, C_4_*), 122.8 (d, *J* = 2.7 Hz, C_1_), 130.2 (d, *J* = 2.3 Hz, C_3_*), 130.6 (d, *J* = 4.0 Hz, C_6_), 156.4 (d, *J* = 7.7 Hz, C_2_ (OCH_3_)), *may be reversed; ^1^H NMR (500 MHz, CDCl_3_) δ 1.18 and 1.34 (t, *J* = 7.1 Hz, 6H, CH_2_C*H*_*3*_), 3.87 (s, 3H, OCH_3_), 3.89–3.97, 4.03–4.10 and 4.19–4.27 (m, 4H, OCH_2_), 5.64 (d, *J* = 14.0 Hz, 1H, CH), 6.88 (d, *J* = 8.3 Hz, 1H, ArH), 7.02 (t, *J* = 7.6 Hz, 1H, ArH), 7.29–7.33 and 7.77–7.79 (m, 2H, ArH); [M + H]^+^ = 293; HRMS *m*/*z*: [M + Na]^+^ calculated for C_12_H_18_ClO_4_PNa 315.0529; found 315.0526.

### 6.6 General Procedure for the Synthesis of Diethyl α-Alkoxy-arylphosphonates

0.50 mmol of diethyl α-methanesulfonyloxy-arylphosphonate (**5a**: 0.16 g; **5d**: 0.17 g; **5e**: 0.18 g), 7.5 mmol of primary alcohol (methyl alcoholl: 0.30 mL, ethyl alcohol: 0.44 mL, butyl alcohol: 0.69 mL), and 0.50 mmol (0.07 mL) of triethylamine were mixed at 135–145 °C for 2–4.5 h under MW irradiation (for the details see Table 2). The reaction mixture was evaporated under vacuum, and the crude product was purified by column chromatography (using DCM–MeOH 97:3 as the eluent on silica gel).

#### 6.6.1 Diethyl α-Methoxybenzylphosphonate (**14a**)

Yield: 0.08 g (63%); colorless oil; ^31^P {^1^H} NMR (202 MHz, CDCl_3_) δ 19.1, δ_P_ lit.^66^ 19.1; [M + H]^+^ = 259; HRMS *m*/*z*: [M + Na]^+^ calculated for C_12_H_19_O_4_PNa 281.0919; found 281.0913.

#### 6.6.2 Diethyl α-Methoxy-4-methylbenzylphosphonate (**14d**)

Yield: 0.11 g (77%); colorless oil; ^31^P {^1^H} NMR (122 MHz, CDCl_3_) δ 19.3, δ_P_ lit.^66^ 19.3; [M + H]^+^ = 273; HRMS *m*/*z*: [M + Na]^+^ calculated for C_13_H_21_O_4_PNa 295.1075; found 295.1068.

#### 6.6.3 Diethyl α-Methoxy-4-chlorobenzylphosphonate (**14e**)

Yield: 0.09 g (60%); colorless oil; ^31^P {^1^H} NMR (202 MHz, CDCl_3_) δ 18.4, δ_P_ lit.^66^ 18.4; [M + H]^+^ = 293; HRMS *m*/*z*: [M + Na]^+^ calculated for C_12_H_18_ClO_4_PNa 315.0529; found 315.0523.

#### 6.6.4 Diethyl α-Ethoxy-benzylphosphonate (**15a**)

Yield: 0.10 g (75%); colorless oil; ^31^P {^1^H} NMR (202 MHz, CDCl_3_) δ 19.2, δ_P_ lit.^67^ 19.6; ^13^C {^1^H} NMR (75 MHz, CDCl_3_) δ 15.2 (s, CH_2_*C*H_3_), 16.4 (t, J = 5.7 Hz, 2 CH_2_*C*H_3_), 62.9 and 63.1 (d, *J* = 7.0 Hz, 2 OCH_2_), 66.5 (d, *J* = 13.8 Hz, OCH_2_), 78.6 (d, *J* = 168.1 Hz, CH), 127.9 (d, *J* = 5.8 Hz, C_β_), 128.2 (d, *J* = 4.2 Hz, C_δ_), 128.3 (d, *J* = 2.5 Hz, C_γ_), 135.1 (d, *J* = 1.8 Hz, C_α_); ^1^H NMR (CDCl_3_) δ lit.^68^ 1.25 (t, *J* = 7.0 Hz, 9H, CH_2_C*H*_*3*_), 3.62 (q, *J* = 7.0 Hz, 2H, OCH_2_), 4.20 (qq, *J* = 7.0 Hz, 4H, OCH_2_), 4.80 and 5.00 (2d, *J* = 15.0 Hz, 1H, CH), 7.45 (s, 5H, ArH); [M + H]^+^ = 273; HRMS *m*/*z*: [M + H]^+^ calculated for C_13_H_22_O_4_P 273.1256; found 273.1260; [M + Na]^+^ calculated for C_13_H_21_O_4_PNa 295.1075; found 295.1083.

#### 6.6.5 Diethyl α-Ethoxy-4-methylbenzylphosphonate (**15d**)

Yield: 0.09 g (66%); colorless oil; ^31^P {^1^H} NMR (122 MHz, CDCl_3_) δ 19.5, δ_P_ lit.^67^ 19.9; ^13^C {^1^H} NMR (CDCl_3_) δ lit.^69^ 14.9 (s, CH_2_*C*H_3_), 16.1 and 16.2 (d, *J* = 6.0 Hz, 2 CH_2_*C*H_3_), 20.9 (s, ArCH_3_), 62.6 and 62.8 (d, *J* = 7.0 Hz, 2 OCH_2_), 66.0 (d, *J* = 14.2 Hz, OCH_2_), 78.2 (d, *J* = 168.0 Hz, CH), 127.7 (d, *J* = 6.0 Hz, C_β_), 128.8 (d, *J* = 2.4 Hz, C_γ_), 131.8 (d, *J* = 1.9 Hz, C_α_), 137.7 (d, *J* = 3.4 Hz, C_δ_); ^1^H NMR (CDCl_3_) δ lit.^69^ 1.15–1.32 (m, 9H, CH_2_C*H*_*3*_), 2.34 (s, 3H, ArCH3), 3.40–3.64 (m, 2H, OCH_2_), 4.15–3.93 (m, 4H, OCH_2_), 4.59 (d, *J* = 15.8 Hz, 1H, CH), 7.16 (d, *J* = 7.4 Hz, 2H, ArH), 7.34 (d, *J* = 6.8 Hz, 2H, ArH); [M + H]^+^ = 287; HRMS *m*/*z*: [M + Na]^+^ calculated for C_14_H_23_O_4_PNa 309.1232; found 309.1229.

#### 6.6.6 Diethyl α-Ethoxy-4-chlorobenzylphosphonate (**15e**)

Yield: 0.10 g (74%); colorless oil; ^31^P {^1^H} NMR (202 MHz, CDCl_3_) δ 18.6; δ_P_ lit.^67^ 18.7; ^13^C {^1^H} NMR (126 MHz, CDCl_3_) δ 15.1 (s, CH_2_*C*H_3_), 16.3 and 16.4 (d, *J* = 5.7 Hz, 2 CH_2_*C*H_3_), 63.0 and 63.3 (d, *J* = 7.0 Hz, 2 OCH_2_), 66.7 (d, *J* = 13.6 Hz, OCH_2_), 78.0 (d, *J* = 168.5 Hz, CH), 128.5 (d, *J* = 2.6 Hz, C_γ_), 129.2 (d, *J* = 5.9 Hz, C_β_), 133.9 (d, *J* = 1.8 Hz, C_α_), 134.1 (d, *J* = 3.8 Hz, C_δ_); ^1^H NMR (CDCl_3_) δ lit.^68^ 1.22 and 1.27 (t, *J* = 7.0 Hz, 9H, CH_2_C*H*_*3*_), 3.60 (q, J = 7.0 Hz, 2H, OCH_2_), 4.06 (qq, 4H, OCH_2_), 4.66 (d, *J* = 16.0 Hz, 1H, CH), 7.44 (s, 4H, ArH); [M + H]^+^ = 307; HRMS *m*/*z*: [M + Na]^+^ calculated for C_13_H_20_ClO_4_PNa 329.0685; found 329.0608.

#### 6.6.7 Diethyl α-Butoxy-benzylphosphonate (**16a**)

Yield: 0.09 g (62%); colorless oil; ^31^P {^1^H} NMR (202 MHz, CDCl_3_) δ 19.2; ^13^C {^1^H} NMR (75 MHz, CDCl_3_) δ 13.8 (s, CH_2_*C*H_3_), 16.3 and 16.4 (d, *J* = 5.8 Hz, 2 CH_2_*C*H_3_), 19.2 (s, *C*H_2_CH_3_), 31.7 (s, CH_2_*C*H_2_), 62.9 and 63.1 (d, J = 6.9 Hz, 2 OCH_2_), 70.9 (d, *J* = 13.5 Hz, OCH_2_), 78.8 (d, *J* = 168.2 Hz, CH), 127.9 (d, *J* = 5.8 Hz, C_β_), 128.2 (d, *J* = 3.2 Hz, C_δ_), 128.3 (d, *J* = 2.5 Hz, C_γ_), 135.2 (d, *J* = 1.8 Hz, C_α_); ^1^H NMR (500 MHz, CDCl_3_) δ 0.91 (t, *J* = 7.4 Hz, 3H, CH_2_C*H*_*3*_), 1.25 and 1.27 (t, *J* = 7.1 Hz, 6H, CH_2_C*H*_*3*_) 1.34–1.46 (m, 2H, C*H*_*2*_CH_3_), 1.56–1.64 (m, 2H, CH_2_C*H*_*2*_), 3.44–3.55 (m, 2H, OCH_2_), 3.95–4.13 (m, 4H, OCH_2_), 4.63 (d, *J* = 16.1 Hz, 1H, CH), 7.31–7.47 (m, 5H, ArH); [M + H]^+^ = 301; HRMS *m*/*z*: [M + Na]^+^ calculated for C_15_H_25_O_4_PNa 323.1388; found 323.1385.

#### 6.6.8 Diethyl α-Butoxy-4-methylbenzylphosphonate (**16d**)

Yield: 0.11 g (70%); colorless oil; ^31^P {^1^H} NMR (122 MHz, CDCl_3_) δ 19.5; ^13^C {^1^H} NMR (75 MHz, CDCl_3_) δ 13.8 (s, CH_2_*C*H_3_), 16.3 and 16.4 (d, *J* = 6.0 Hz, 2 CH_2_*C*H_3_), 19.2 (s, *C*H_2_CH_3_), 21.2 (s, ArCH_3_), 31.7 (s, CH_2_*C*H_2_), 62.8 and 63.1 (d, J = 6.9 Hz, 2 OCH_2_), 70.7 (d, *J* = 13.7 Hz, OCH_2_), 78.6 (d, *J* = 169.0 Hz, CH), 127.9 (d, *J* = 5.9 Hz, C_β_), 129.0 (d, *J* = 2.5 Hz, C_γ_), 132.1 (d, *J* = 1.9 Hz, C_α_), 138.0 (d, *J* = 3.3 Hz, C_δ_); ^1^H NMR (500 MHz, CDCl_3_) δ 0.91 (t, *J* = 7.4 Hz, 3H, CH_2_C*H*_*3*_), 1.25 and 1.28 (t, *J* = 6.8 Hz, 6H, CH_2_C*H*_*3*_) 1.35–1.43 (m, 2H, C*H*_*2*_CH_3_), 1.59–1.62 (m, 2H, CH_2_C*H*_*2*_), 2.37 (s, ArCH_3_), 3.41–3.54 (m, 2H, OCH_2_), 3.95–4.13 (m, 4H, OCH_2_); 4.59 (d, *J* = 15.7 Hz, 1H, CH), 7.18–7.20 and 7.34–7.36 (m, 4H, ArH); [M + H]^+^ = 315; HRMS *m*/*z*: [M + Na]^+^ calculated for C_16_H_27_O_4_PNa 337.1545; found 337.1533.

#### 6.6.9 Diethyl α-Butoxy-4-chlorobenzylphosphonate (**16e**)

Yield: 0.11 g (61%); colorless oil; ^31^P {^1^H} NMR (202 MHz, CDCl_3_) δ 18.6; ^13^C {^1^H} NMR (126 MHz, CDCl_3_) δ 13.8 (s, CH_2_*C*H_3_), 16.3 and 16.4 (d, *J* = 5.7 Hz, 2 CH_2_*C*H_3_), 19.2 (s, *C*H_2_CH_3_), 31.7 (s, CH_2_*C*H_2_), 63.0 and 63.2 (d, *J* = 7.0 Hz, 2 OCH_2_), 71.1 (d, *J* = 13.2 Hz, OCH_2_), 78.2 (d, *J* = 168.9 Hz, CH), 128.5 (d, *J* = 2.7 Hz, C_γ_), 129.2 (d, *J* = 5.8 Hz, C_β_), 133.9 (d, *J* = 1.8 Hz, C_α_), 134.1 (d, *J* = 3.8 Hz, C_δ_); ^1^H NMR (500 MHz, CDCl_3_) δ 0.91 (t, *J* = 7.4 Hz, 3H, CH_2_C*H*_*3*_), 1.26 and 1.28 (t, *J* = 7.1 Hz, 6H, CH_2_C*H*_*3*_) 1.34–1.43 (m, 2H, C*H*_*2*_CH_3_), 1.60–1.63 (m, 2H, CH_2_C*H*_*2*_), 3.44–3.53 (m, 2H, OCH_2_), 3.99–4.14 (m, 4H, OCH_2_); 4.59 (d, *J* = 16.1 Hz, 1H, CH), 7.35–7.43 (m, 4H, ArH); [M + H]^+^ = 335; HRMS *m*/*z*: [M + Na]^+^ calculated for C_15_H_24_ClO_4_PNa 357.0998; found 357.1006.

### 6.7 General Procedure for the Synthesis of Tetraethyl 4-Substituted-(phenylmethylene)bisphosphonates

0.50 mmol of diethyl **α-methanesulfonyloxy-arylphosphonate** (**5a**: 0.16 g; **5d**: 0.17 g; **5e**: 0.18 g), 2.5 or 4.5 mmol of triethyl phosphite (0.43 or 0.77 mL, respectively) were mixed at 135 °C for 3 or 4 days in a sealed tube (for the details see Table 4). The crude product was purified by column chromatography (using DCM–MeOH 97:3 as the eluent on silica gel).

#### 6.7.1 Tetraethyl (Phenylmethylene)bisphosphonates (**18a**)

Yield: 0.15 g (80%); colorless oil; ^31^P {^1^H} NMR (202 MHz, CDCl_3_) δ 18.6, δ_P_ lit.^51^ 19.1 [M + H]^+^ = 365; HRMS *m*/*z*: [M + Na]^+^ calculated for C_15_H_26_O_6_P_2_Na 387.1102; found 387.1099.

#### 6.7.2 Tetraethyl (4-Methylphenyl)methylenebisphosphonates (**18d**)

Yield: 0.14 g (76%); colorless oil; ^31^P {^1^H} NMR (202 MHz, CDCl_3_) δ 18.8; ^13^C {^1^H} NMR (75 MHz, CDCl_3_) δ 16.1–16.3 (m, 4 CH_2_*C*H_3_), 21.1 (s, ArCH_3_), 45.2 (t, *J* = 133.0 Hz, CH), 62.8–62.8 and 63.3–63.4 (dm, 4 OCH_2_), 126.8 (t, *J* = 7.8 Hz, C_α_), 129.2 (t, *J* = 2.1 Hz, C_γ_), 130.2 (t, *J* = 6.4 Hz, C_β_), 137.3 (t, *J* = 2.7 Hz, C_δ_); ^1^H NMR (500 MHz, CDCl_3_) δ 1.18 and 1.30 (t, *J* = 7.1 Hz, 12H, CH_2_C*H*_*3*_), 2.35 (s, 3H, ArCH_3_), 3.72 (t, *J* = 25.1 Hz, 1H, CH), 3.93–4.18 (m, 8H, OCH_2_), 7.15 (d, *J* = 7.8 Hz, 2H, ArH), 7.36–7.38 (m, 2H, ArH); [M + H]^+^ = 379; HRMS *m*/*z*: [M + Na]^+^ calculated for C_16_H_28_O_6_P_2_Na 401.1259; found 401.1260.

#### 6.7.3 Tetraethyl (4-Chlorophenyl)methylenebisphosphonates (**18e**)

Yield: 0.16 g (81%); colorless oil; ^31^P {^1^H} NMR (202 MHz, CDCl_3_) δ 18.1, δ_P_ lit.^52^ 15.3; ^13^C {^1^H} NMR (126 MHz, CDCl_3_) δ 15.2 and 15.3 (d, *J* = 6.2 Hz, 4 CH_2_*C*H_3_), 44.1 (t, *J* = 133.2 Hz, CH), 62.1 and 62.5 (d, *J* = 6.5 Hz, 4 OCH_2_), 127.7 (t, *J* = 2.0 Hz, C_γ_), 127.9 (t, *J* = 7.8 Hz, C_α_), 130.7 (t, *J* = 6.4 Hz, C_β_), 132.7 (t, *J* = 3.0 Hz, C_δ_); ^1^H NMR (500 MHz, CDCl_3_) δ 1.21 and 1.31 (t, *J* = 7.1 Hz, 6H, CH_2_C*H*_*3*_), 3.72 (t, *J* = 25.0 Hz, 1H, CH), 3.98–4.19 (m, 4H, OCH_2_), 7.33–7.49 (m, 4H ArH); [M + H]^+^ = 399; HRMS *m*/*z*: [M + Na]^+^ calculated for C_15_H_25_ClO_6_P_2_Na 421.0713; found 421.0707.

### 6.8 Experimental for Computations

DFT computations at the M062*X*/6-31+G (d,p) level of theory were performed considering the solvent effect of toluene using the SMD (universal solvation model based on solute electron density) solvent model with the Gaussian 09 program package.^70−72^ The geometries of the molecules were optimized in all cases, and frequency calculations were also performed to ensure that the structures were at a local minimum. The solution-phase enthalpies and Gibbs free energies were obtained by frequency calculations as well. The H and G values obtained were given under standard conditions; the standard state correction and the corrected total energies of the molecules were considered. Entropic and thermal corrections were evaluated for isolated molecules using standard rigid rotor harmonic oscillator approximations, that is, the Gibbs free energy was taken as the “sum of electronic and thermal free energies” printed in a Gaussian 09 vibrational frequency calculation. The standard state correction was taken into account. For details of the calculations, see the Supporting Information.

### 6.9 Single X-ray Experimental

Single crystals of compound **5e**, suitable for X-ray diffraction, were obtained by slow evaporation of acetone solution. The crystals were introduced into perfluorinated oil and a suitable single crystal was carefully mounted on the top of a thin glass wire. Data collection was performed with an Oxford Xcalibur 3 diffractometer equipped with a Spellman generator (50 kV, 40 mA) and a Kappa CCD detector, operating with Mo–K_α_ radiation (λ = 0.71071 Å).

Data collection and data reduction were performed with the CrysAlisPro software.^73^ Absorption correction using the multiscan method^74^ was applied. The structures were solved with SHELXS-97,^75^ refined with SHELXL-97^75^ and finally checked using PLATON.^76^ Details for data collection and structure refinement are summarized in Table 7.

**Table 7:** Details for X-ray Data Collection and Structure Refinement for Compound **5e**

	5e
empirical formula	C_12_H_18_ClO_6_PS
formula mass	356.74
*T*[K]	123(2)
crystal size [mm]	0.25 × 0.15 × 0.10
crystal description	colorless block
crystal system	monoclinic
space group	*P*21/*n*
*a* [Å]	5.30830(10)
*b* [Å]	17.8886(4)
*c* [Å]	17.1694(4)
α [°]	90.0
β [°]	97.308(2)
γ [°]	90.0
*V* [Å^3^]	1617.13(6)
*Z*	4
ρ_calcd._ [g cm^–3^]	1.465
μ [mm^–1^]	0.486
*F*(000)	744
Θ range [°]	2.39–25.24
index ranges	–7 ≤ *h* ≤ 7
	–25 ≤ *k* ≤ 25
	–24 ≤ *l* ≤ 24
reflns. collected	32826
reflns. obsd.	3935
reflns. unique	4954 (*R*_int_ = 0.0408)
*R*_1_, *wR*_2_ (2σ data)	0.0356, 0.0789
*R*_1_, *wR*_2_ (all data)	0.0520, 0.0863
GOOF on *F*^2^	1.041
peak/hole [e Å^–3^]	0.424/–0.360

CCDC-2351759 contains supplementary crystallographic data for this compound. These data can be obtained free of charge from The Cambridge Crystallographic Data Centre via www.ccdc.cam.ac.uk/data_request/cif.

## 7 Bioactivity Experimental

### 7.1 Cell Culturing and Evaluation of In Vitro Cytostasis on Tumor Cell Lines

Cytostatic effect of the compounds was studied on tumor cell cultures in vitro. MDA-MB-231 human breast adenocarcinoma^77^ cells were cultured in DMEM medium supplemented with 10% FBS, 2 mM l-glutamine, penicillin-streptomycin antibiotics mixture (50 IU/mL and 50 μg/mL, respectively), 1 mM sodium pyruvate and 1% nonessential amino acid mixture. A2058^78^ human melanoma cells were cultured in RPMI medium supplemented with 10% FBS, 2 mM l-glutamine, and penicillin-streptomycin antibiotics mixture (50 IU/mL and 50 μg/mL, respectively). The cultures were maintained at 37 °C in a humidified atmosphere with 5% CO_2_. The cells were grown to confluence, and then they were divided into 96-well tissue culture plates with the initial cell number of 5.0 × 10^3^ cells/well. After 24 h incubation at 37 °C, the cells were treated with the compounds in 200 μL final volume containing 1.0 v/v% DMSO at 10–250 μM (daunomycin: 0.016–10 μM, tamoxifen: 0.16–100 μM) concentration overnight. Control cells were treated with serum-free medium only or with DMSO (*c* = 1.0 v/v %) at the same conditions. After this incubation period, cells were washed twice with serum-free medium and following that, they were cultured for another 72 h in 10% serum-containing medium at 37 °C. After that, cell viability was determined by Alamar Blue assay. Alamar Blue is a nontoxic, resazurin-based dye that is reduced by living cells to a fluorescent molecule, resorufin.^79^ Resazurin sodium salt (Merck, Darmstadt, Germany) was dissolved in phosphate-buffered saline at *c* = 0.15 mg/mL, pH 7.4. 32.5 μL of the dye was added to each well and incubated at 37 °C for 3 h until the pink color of the reduced dye appeared. Fluorescence intensity in each well was measured using a Synergy H4 multimode microplate reader (BioTek, Winooski, VT); at λ_ex_ = 530/30 and λ_em_ = 610/10 nm. Cytostatic effect was calculated with the following equation:
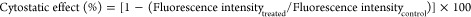


Cytostasis values were expressed in the percentage of untreated control. 50% inhibitory concentration (IC_50_) was determined by fitting a sigmoid curve on the data points using Microcal Origin2021 software and the calculating *X* values at *Y* = 50 and expressed in micromolar units.

## Data Availability

The data underlying this study are available in the published article and its Supporting Information.

## Supporting Information Available

^31^P, ^13^C, and ^1^H NMR spectra, the geometrical data for compound **5e**, as well as data of the theoretical calculations (PDF)
