# Supplementary material for: Synthesis of Mesylated and Tosylated α-Hydroxy-Benzylphosphonates; Their Reactivity and Cytostatic Activity
Source: ACS Omega. 2024 Jul 2;9(28):31043–55. doi: 10.1021/acsomega.4c04382 (PMC11256086; doi:10.1021/acsomega.4c04382)
Supplement: Supplementary file 1 — ao4c04382_si_001.pdf [file ao4c04382_si_001.pdf]

Supporting Information

Synthesis of Mesylated and Tosylated α-Hydroxy-benzylphosphonates; their Reactivity and Cytostatic Activity

Zsuzsanna Szalai,[1] Márton Debrei,[1] Péter Ábrányi-Balogh,[1,2,3] Szilvia Bősze,[4] Rita Oláhné Szabó,[4,5,]*Konstantin Karaghiosoff,[6] László Drahos,[7] and György Keglevich[1,]*

[1]*Department of Organic Chemistry and Technology, Faculty of Chemical Technology and Biotechnology, Budapest University of Technology and Economics, 1111 Budapest, Műegyetem rkp. 3., Hungary; keglevich.gyorgy@vbk.bme.hu.*

[2]*Medicinal Chemistry Research Group, HUN-REN Research Centre for Natural Sciences, 1117 Budapest, Hungary.*

[3]*National Drug Research and Development Laboratory, HUN-REN Research Centre for Natural Sciences, 1117 Budapest, Hungary.*

[4]*Hungarian Research Network (HUN-REN), HUN-REN-ELTE Research Group of Peptide Chemistry, Eötvös Loránd University, 1117 Budapest, Hungary; rita.szabo@ttk.elte.hu*

[5]*Department of Genetics, Cell-and Immunobiology, Semmelweis University, Nagyvárad tér 4, 1089 Budapest, Hungary*

[6]*Department Chemie, Ludwig-Maximilians-Universität München, Butenandtstr. 5-13, D-81377 München, Germany*

[7]*MS Proteomics Research Group, Research Centre for Natural Sciences, 1117 Budapest, Hungary*

Table of Contents

1. Spectra of the compounds **2b**, **3b**, **4a**,**b**, **4d-f**, **5a-f**, **6a**,**d**,**e**, **8g**, **9g**,**h**, **14a**,**d**,**e**, **15a**,**d**,**e**, **16a**,**d**,**e**, **18a**,**d**,**e** synthesized 2
2. Geometrical data for compound **5e** obtained from the X-ray measurement 40
3. Computed energy values and coordinates 43

1. Spectra of the compounds 2b, 3b, 4a,b, 4d-f, 5a-f, 6a,d,e, 8g, 9g,h, 14a,d,e, 15a,d,e, 16a,d,e, 18a,d,e synthesized

^{31}P {^{1}H} NMR (202 MHz, $CDCl_3$) spectra for 2b

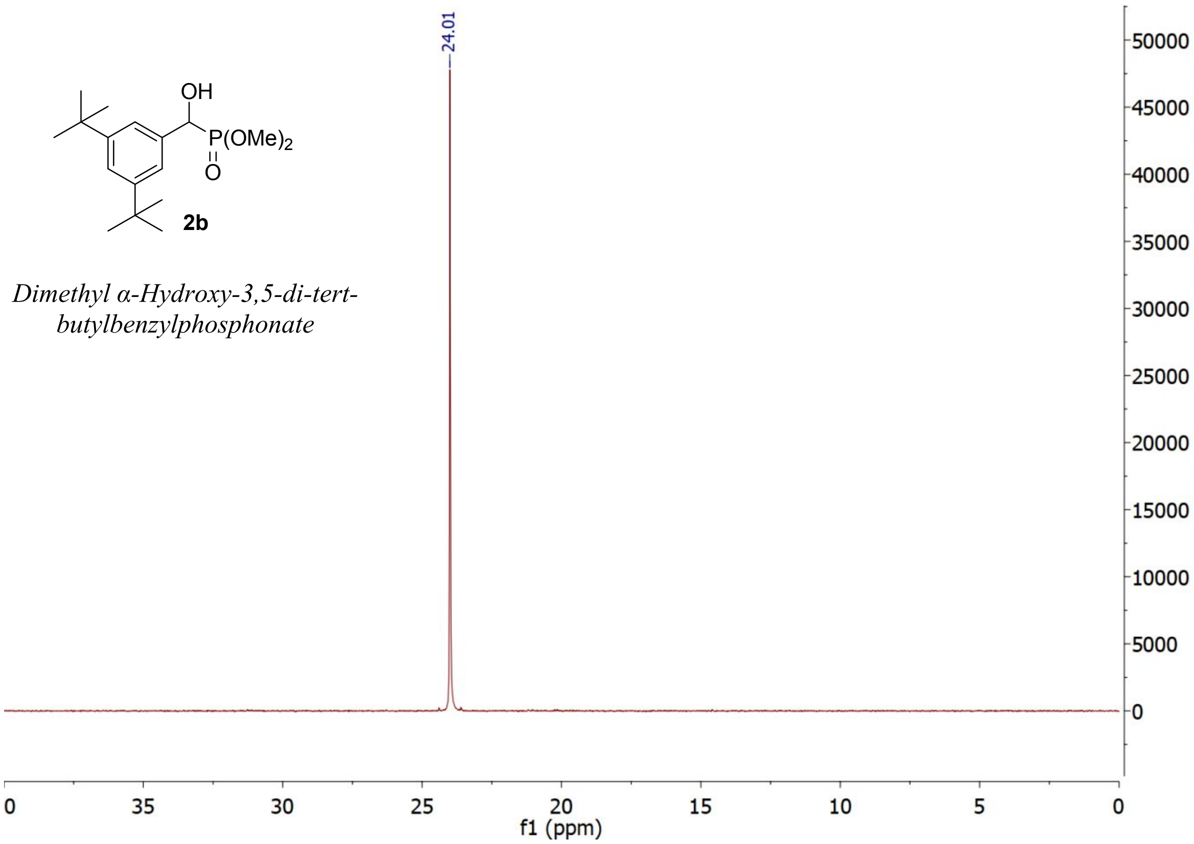

^{13}C {^{1}H} NMR (126 MHz, $CDCl_3$) spectra for 2b

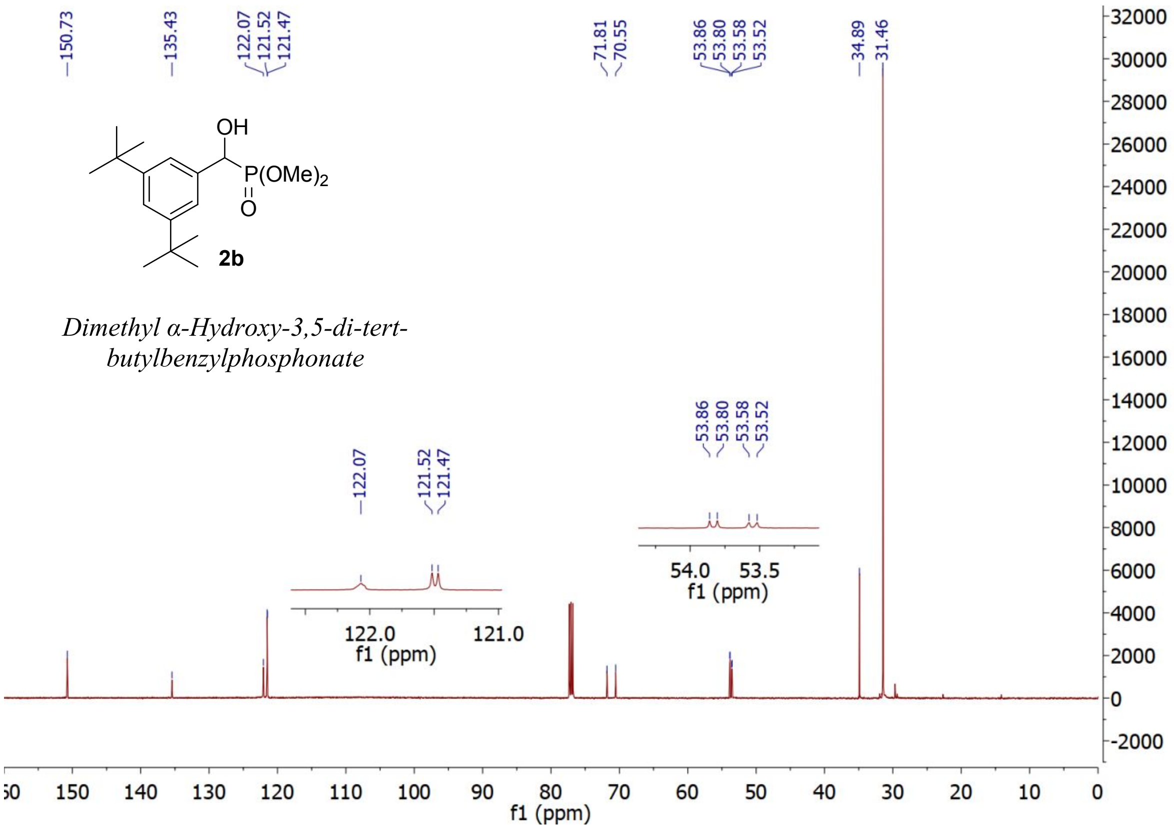

^{1}H NMR (500 MHz, $CDCl_3$) spectra for 2b

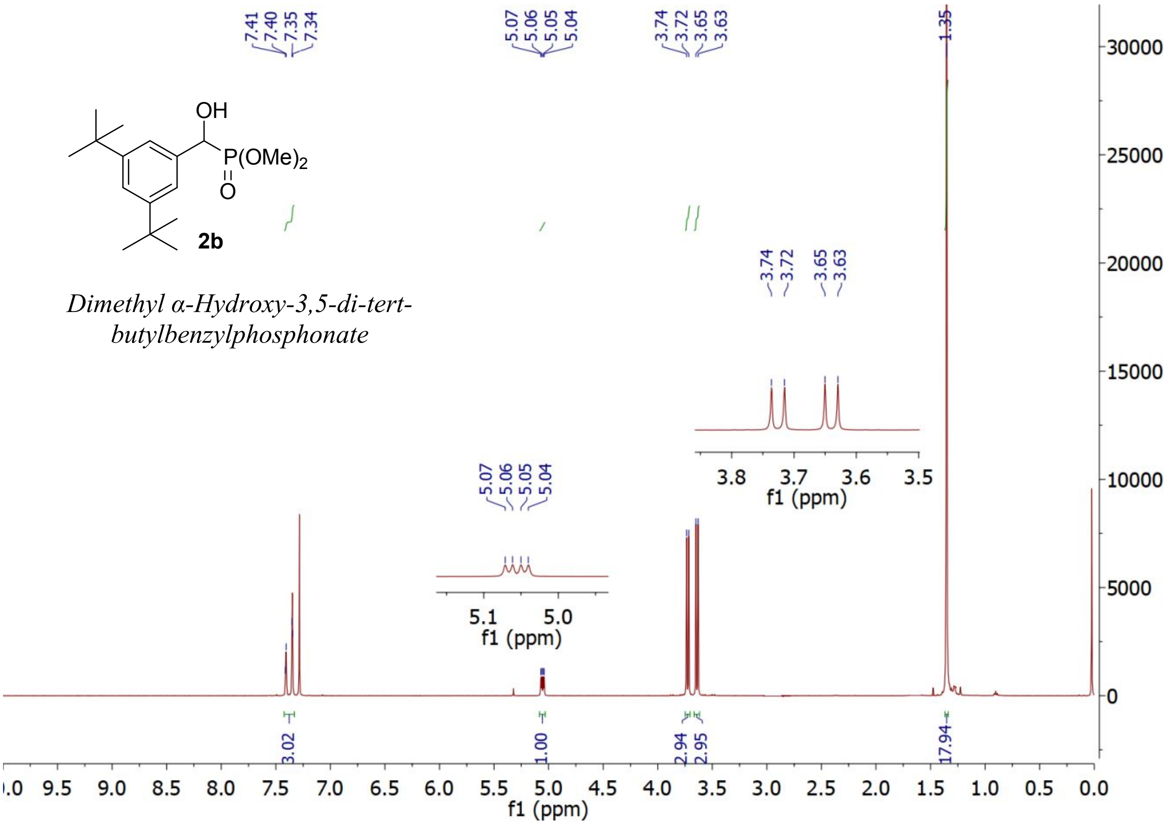

^{31}P {^{1}H} NMR (202 MHz, $CDCl_3$) spectra for 3b

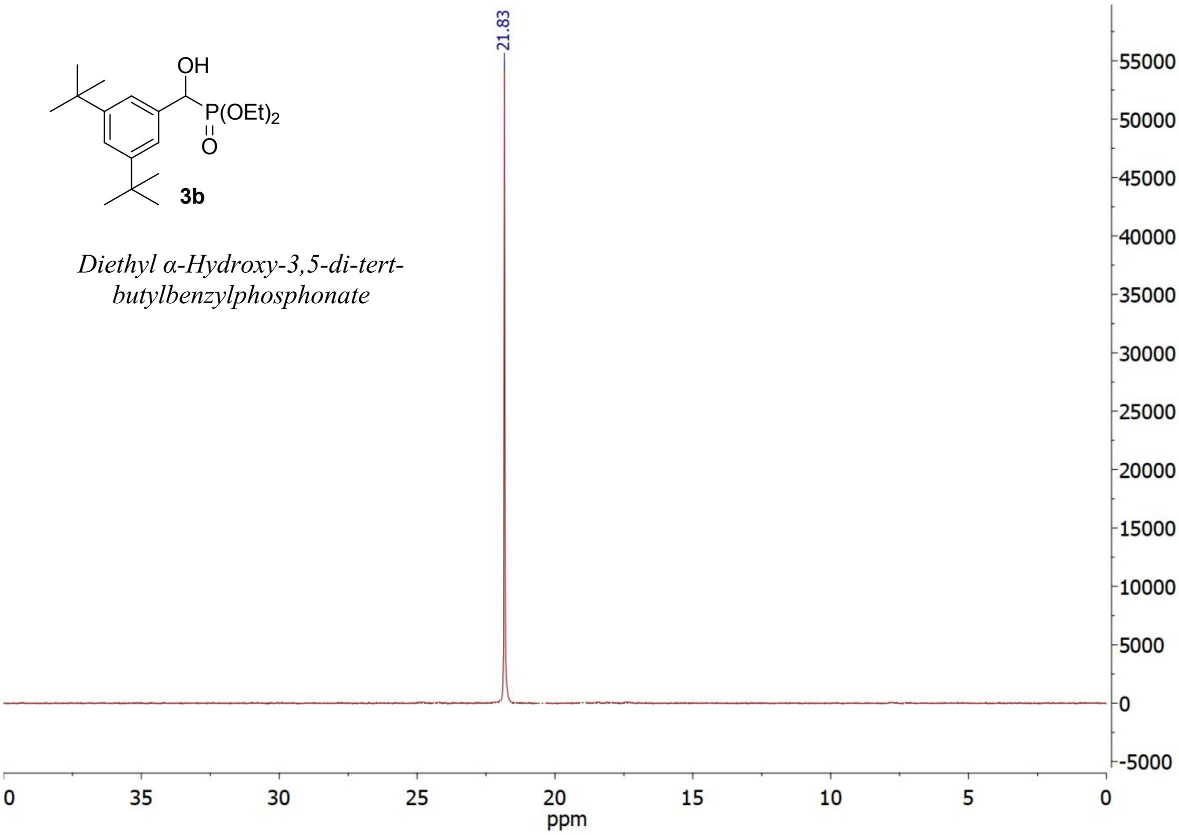

^{13}C {^{1}H} NMR (126 MHz, $CDCl_3$) spectra for 3b

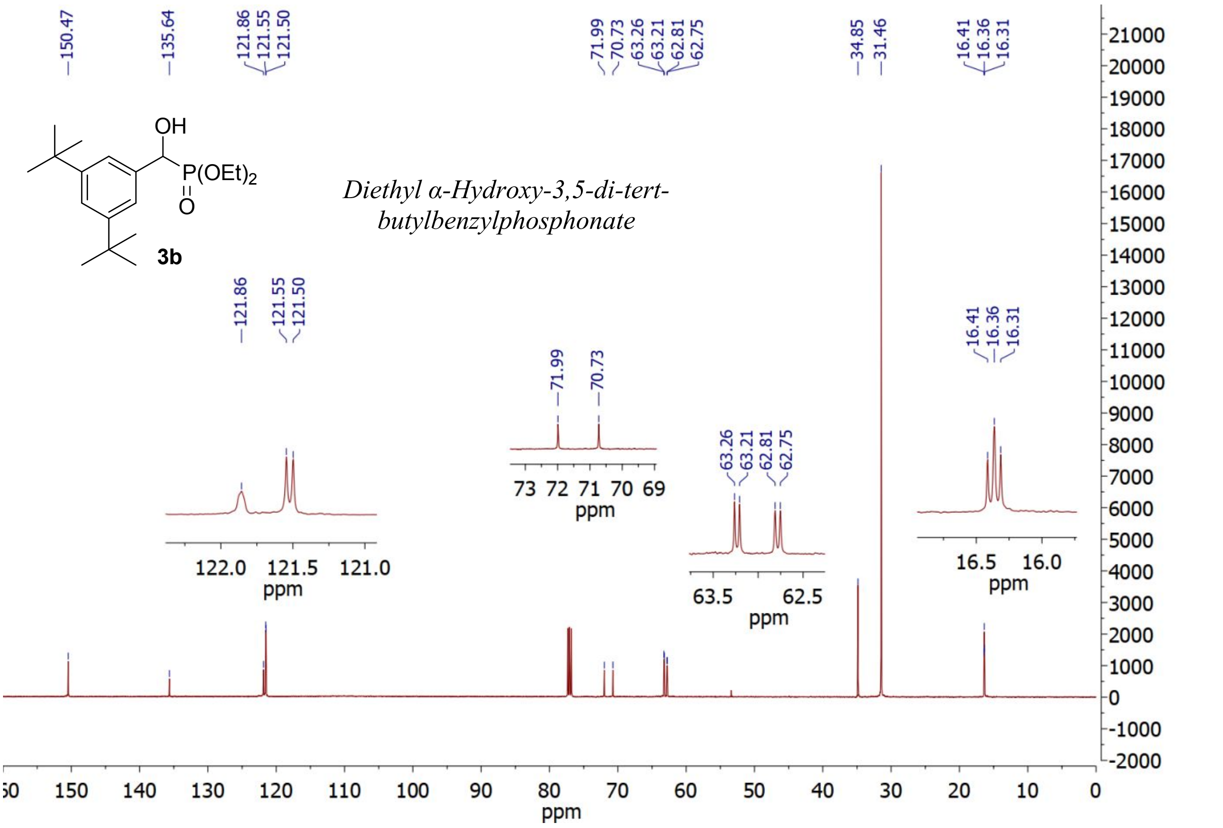

^{1}H NMR (500 MHz, $CDCl_3$) spectra for 3b

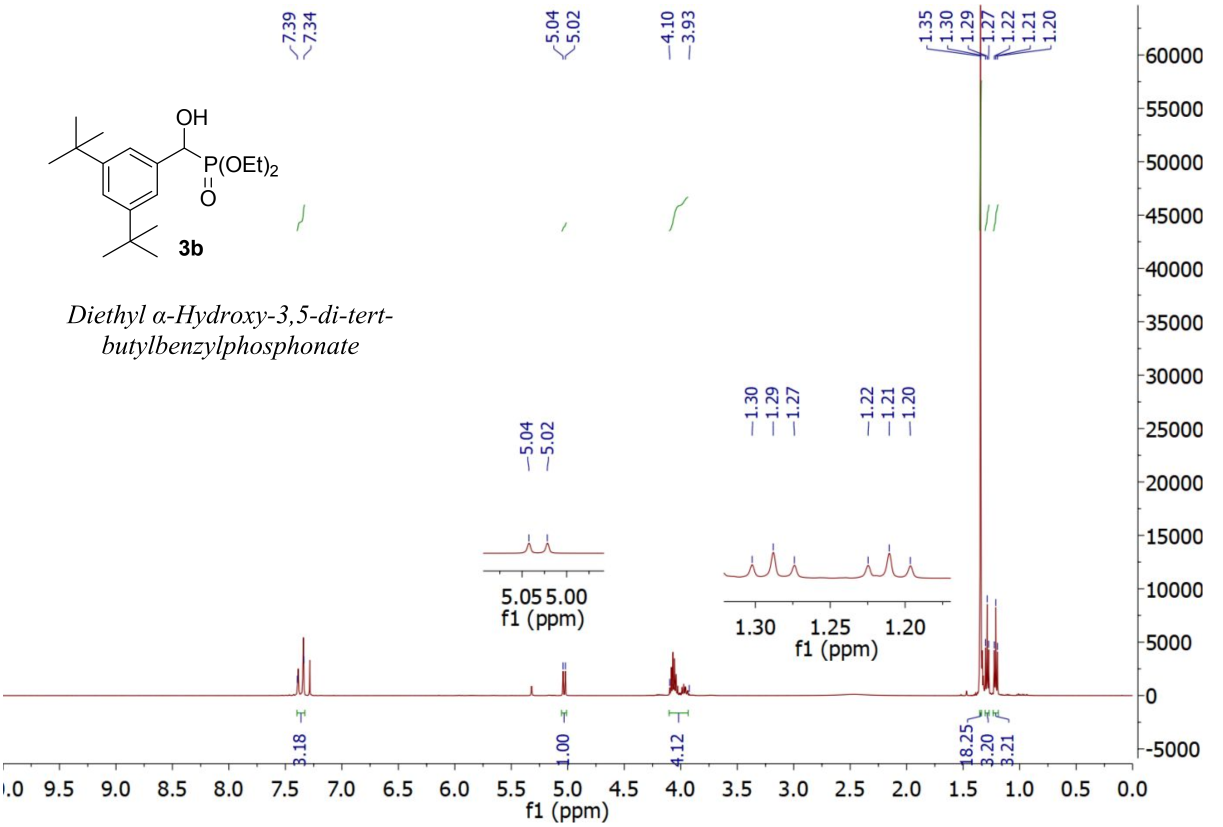

$^{31}P\{^1H\}$ NMR (202 MHz, $CDCl_3$) spectra for 4a

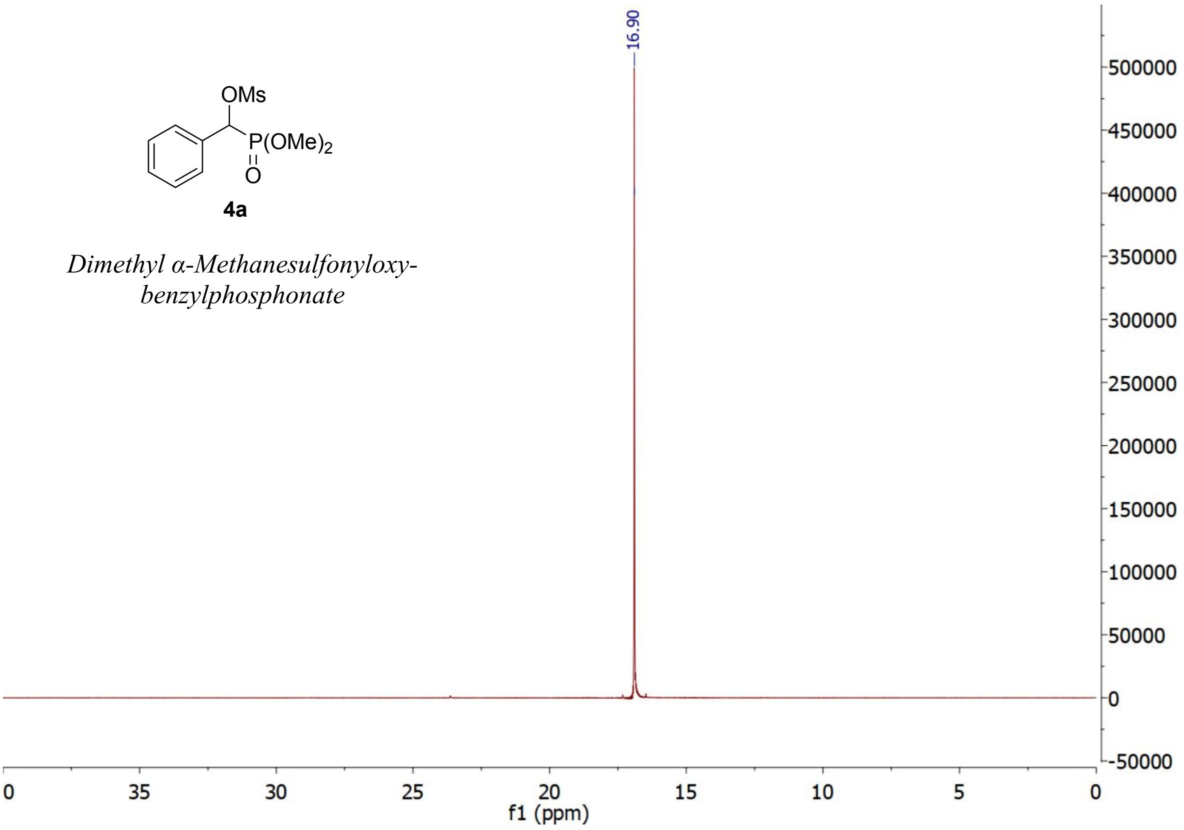

$^{13}C\{^1H\}$ NMR (126 MHz, $CDCl_3$) spectra for 4a

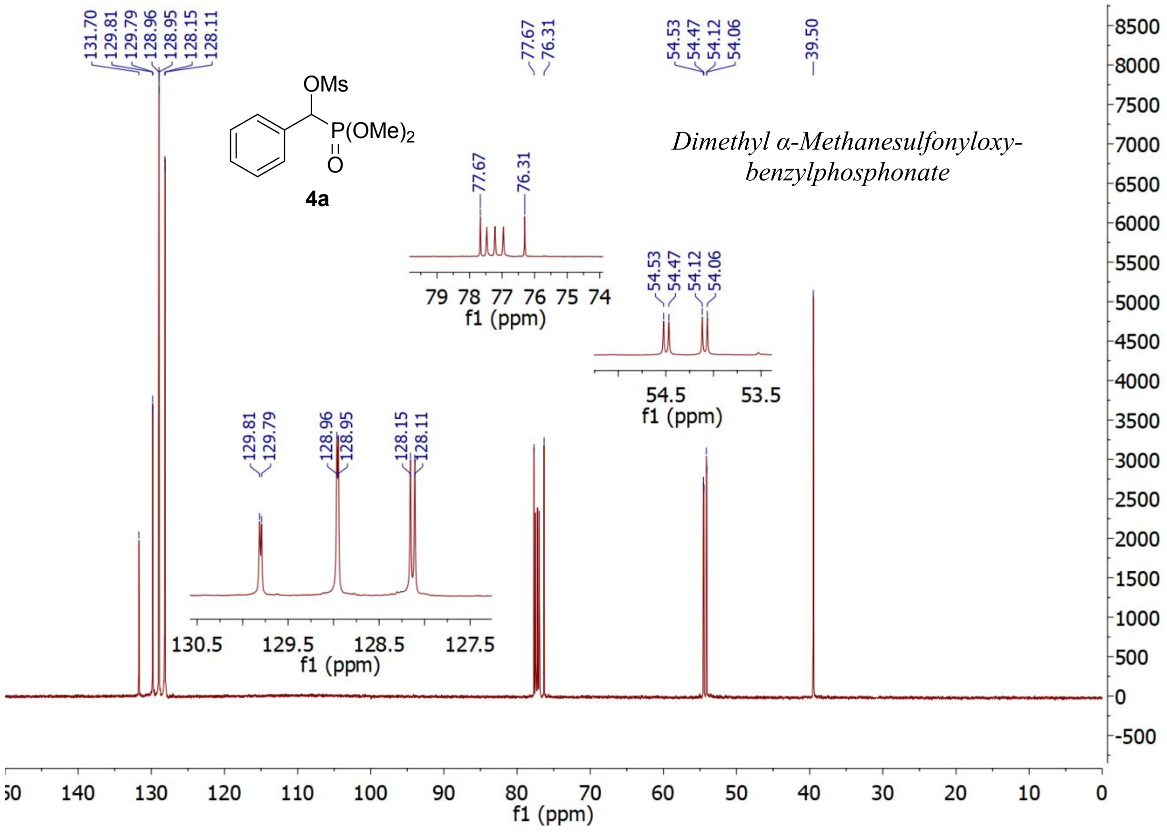

^{1}H NMR (500 MHz, $CDCl_3$) spectra for 4a

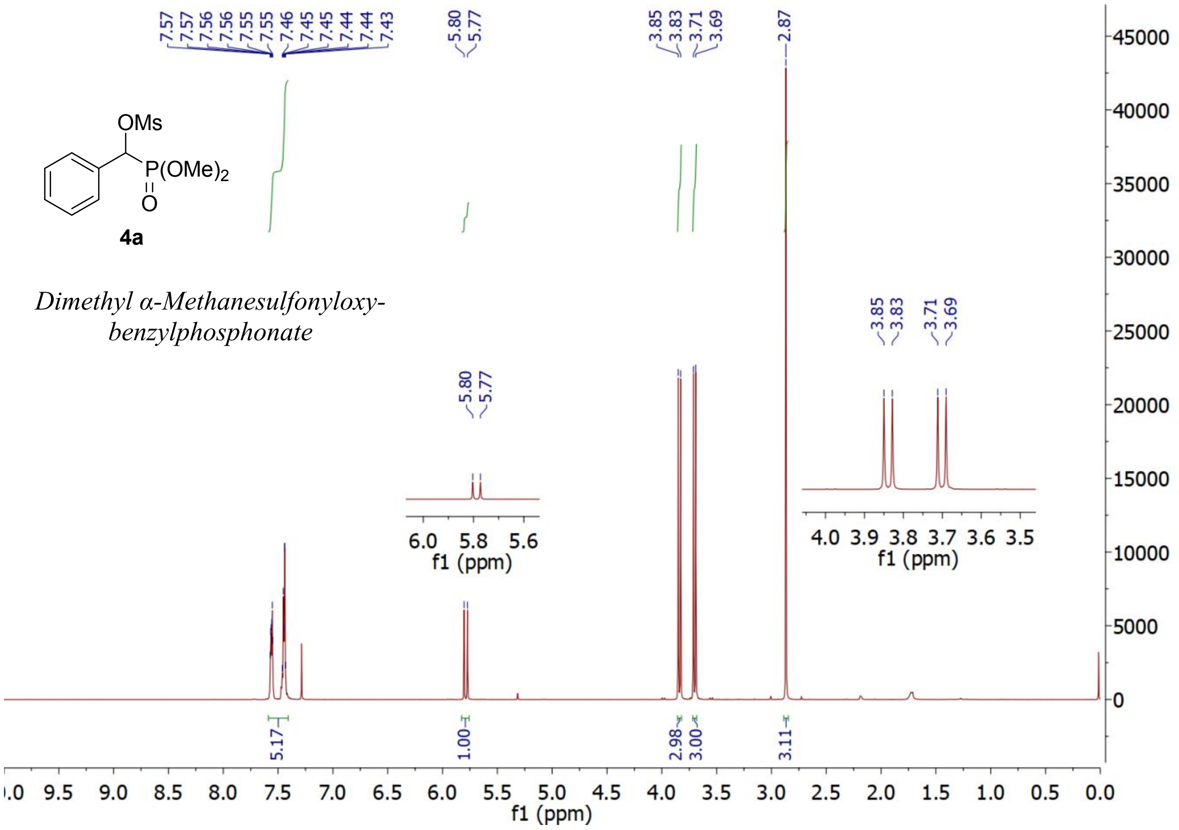

^{31}P {^{1}H} NMR (202 MHz, $CDCl_3$) spectra for 4b

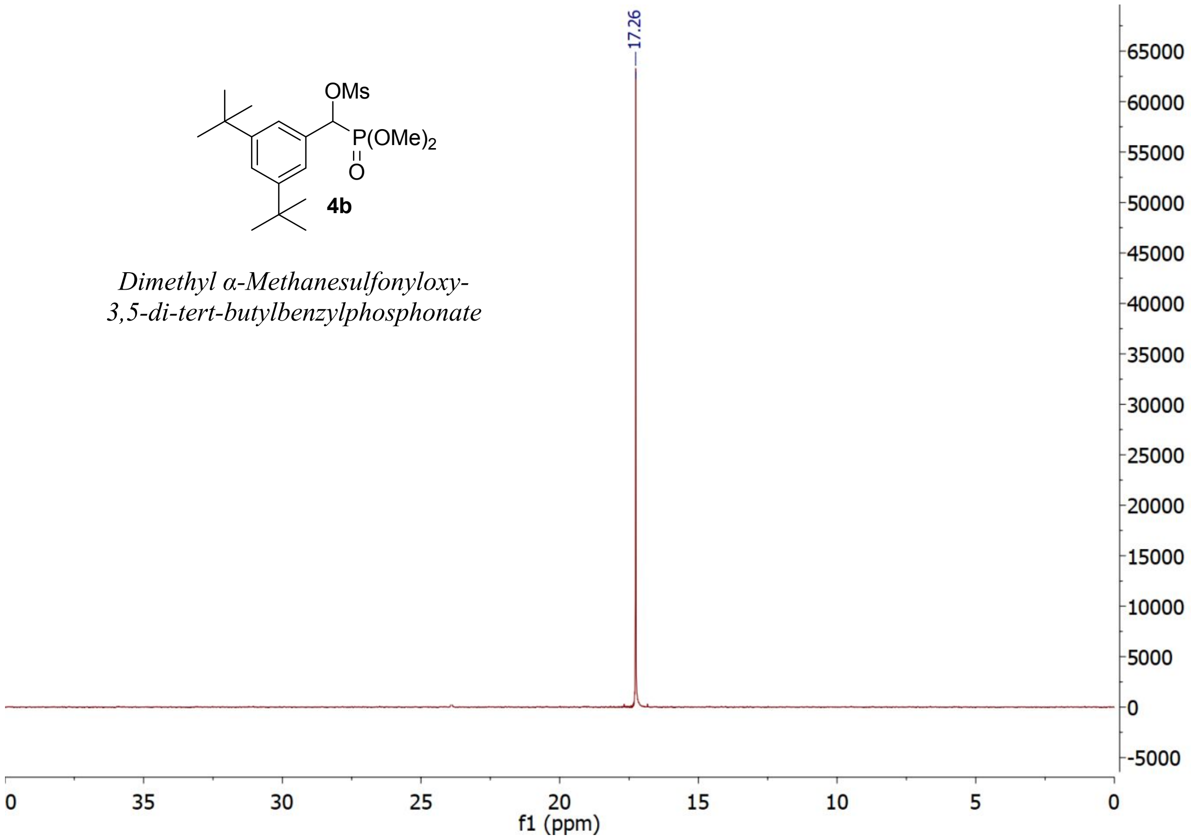

^{13}C {^{1}H} NMR (126 MHz, $CDCl_3$) spectra for 4b

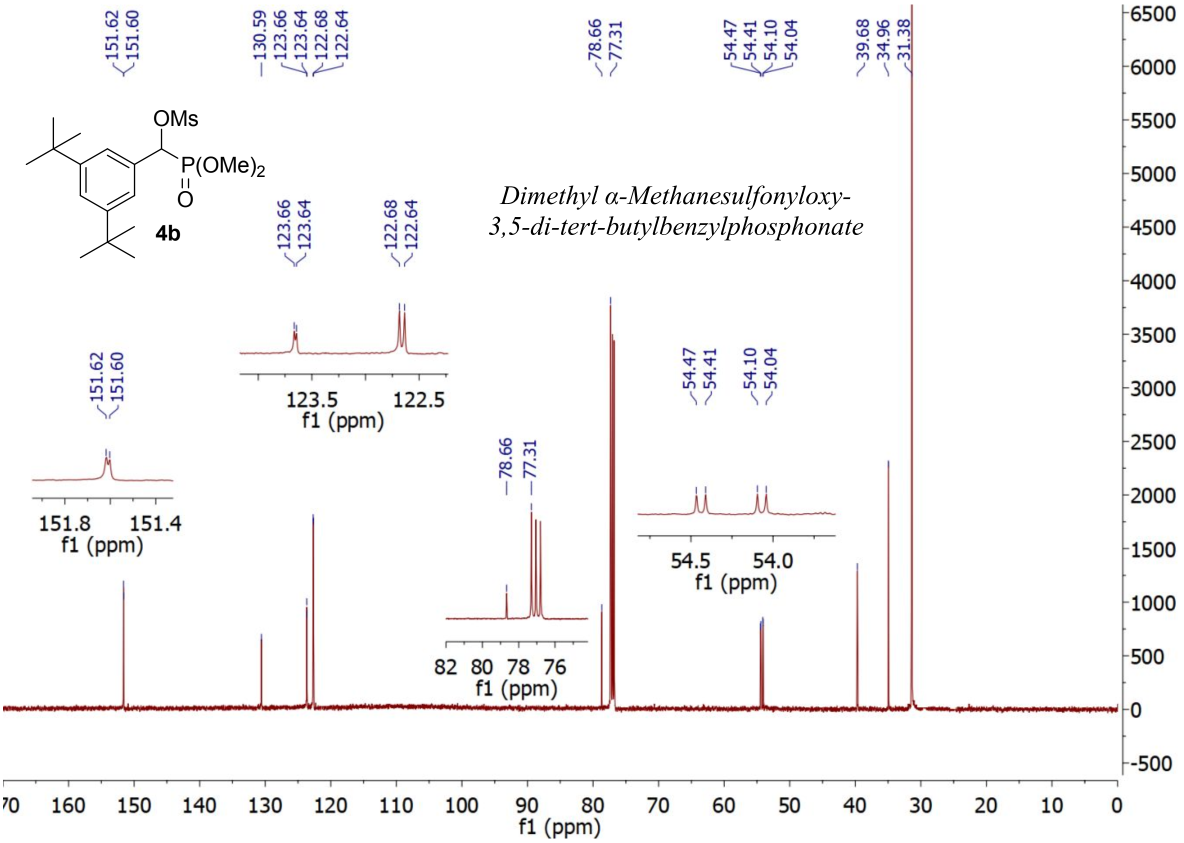

^{1}H NMR (500 MHz, $CDCl_3$) spectra for 4b

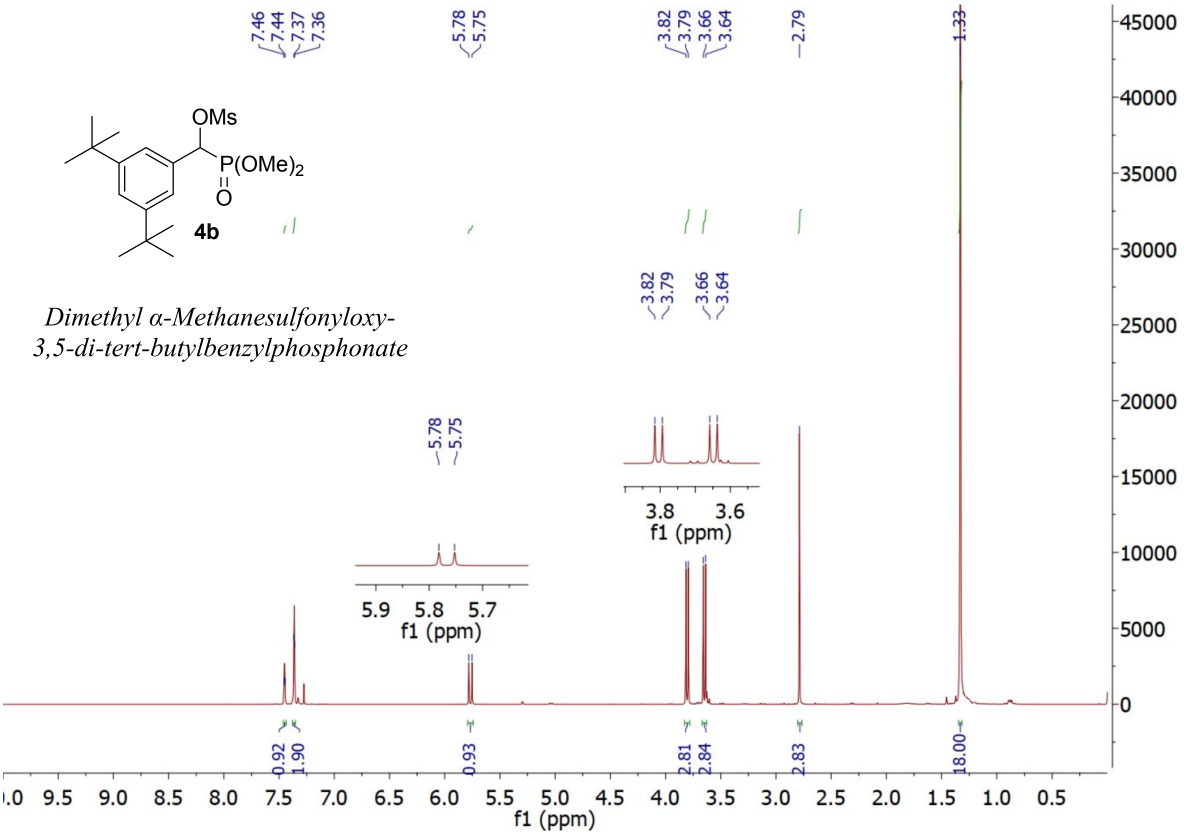

^{31}P {^{1}H} NMR (202 MHz, $CDCl_3$) spectra for 4d

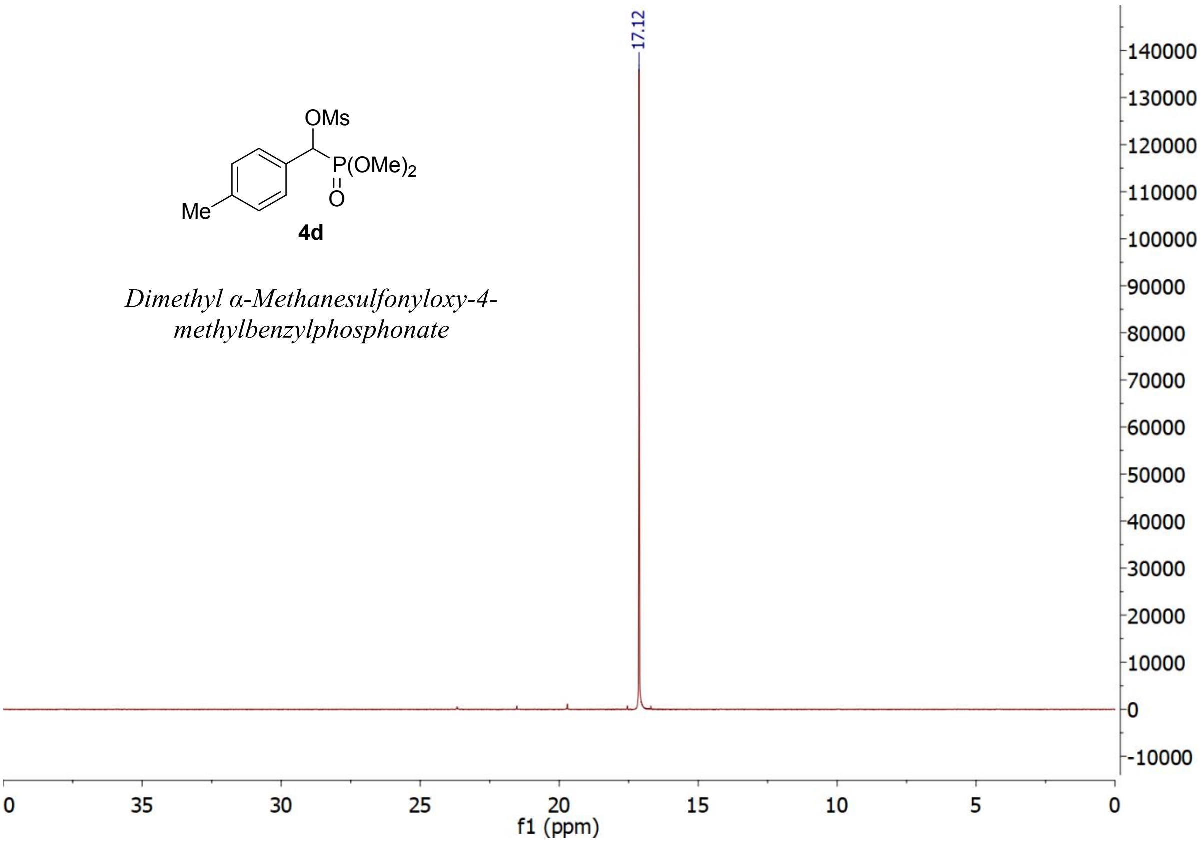

^{13}C {^{1}H} NMR (126 MHz, $CDCl_3$) spectra for 4d

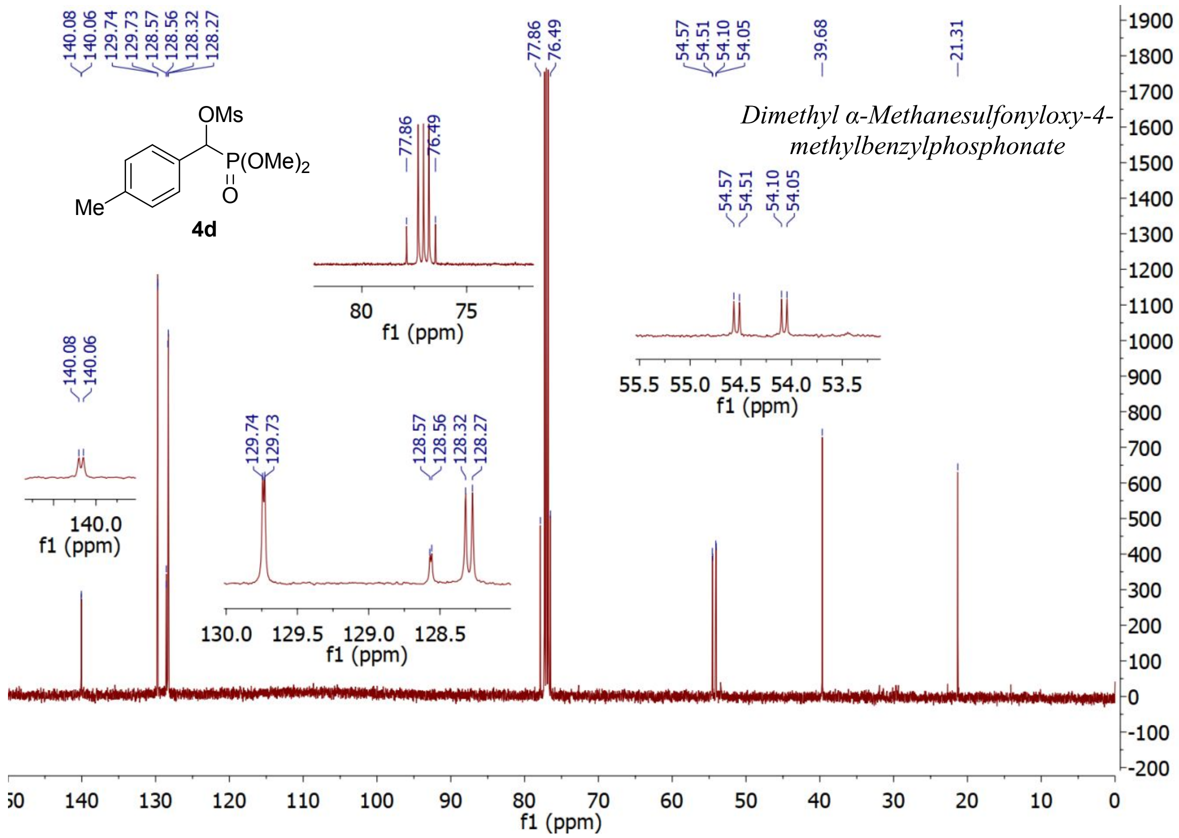

^{1}H NMR (500 MHz, $CDCl_3$) spectra for 4d

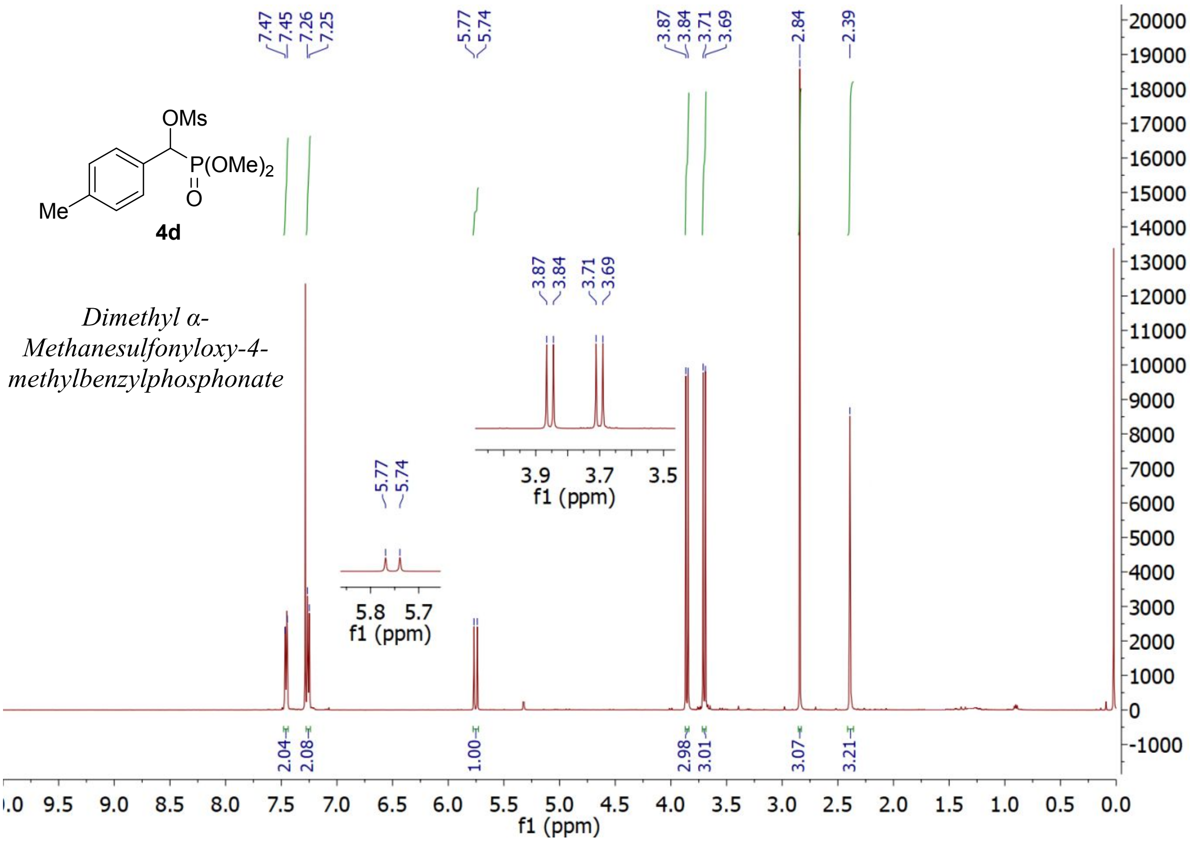

^{31}P {^{1}H} NMR (202 MHz, $CDCl_3$) spectra for 4e

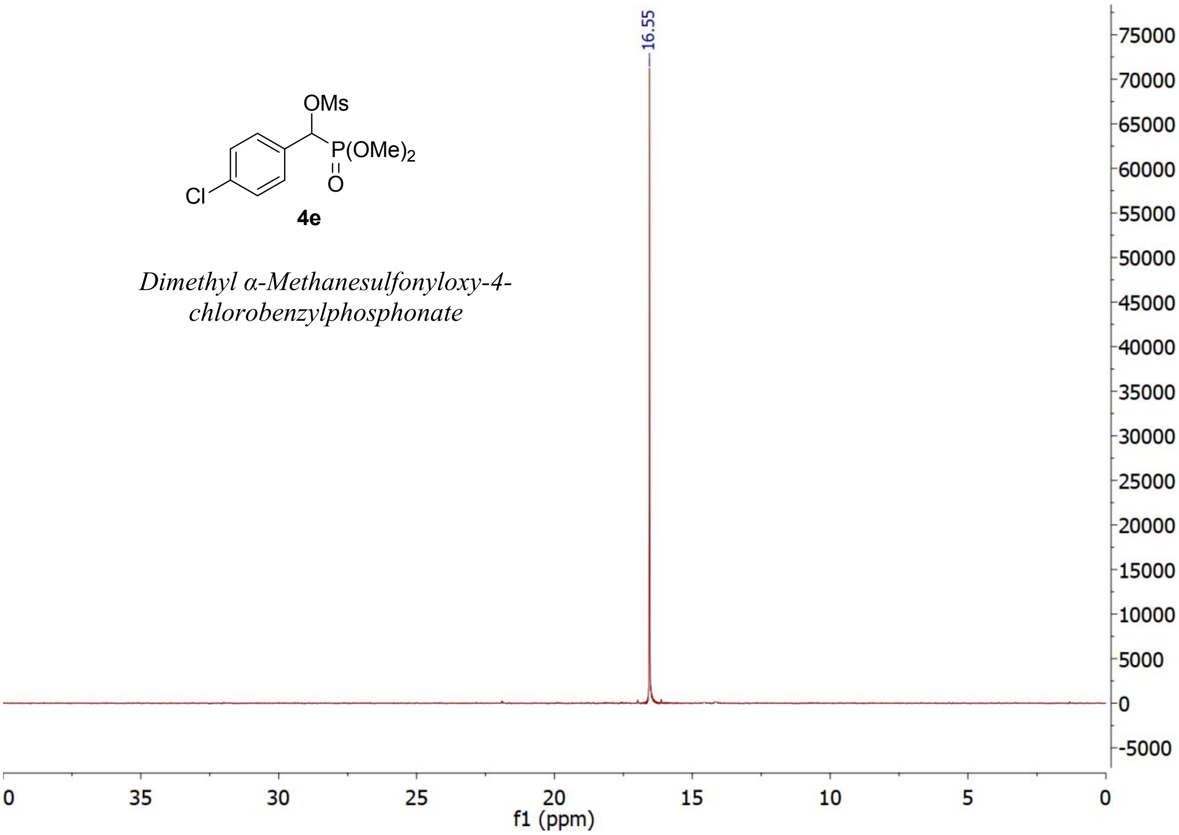

^{13}C {^{1}H} NMR (126 MHz, $CDCl_3$) spectra for 4e

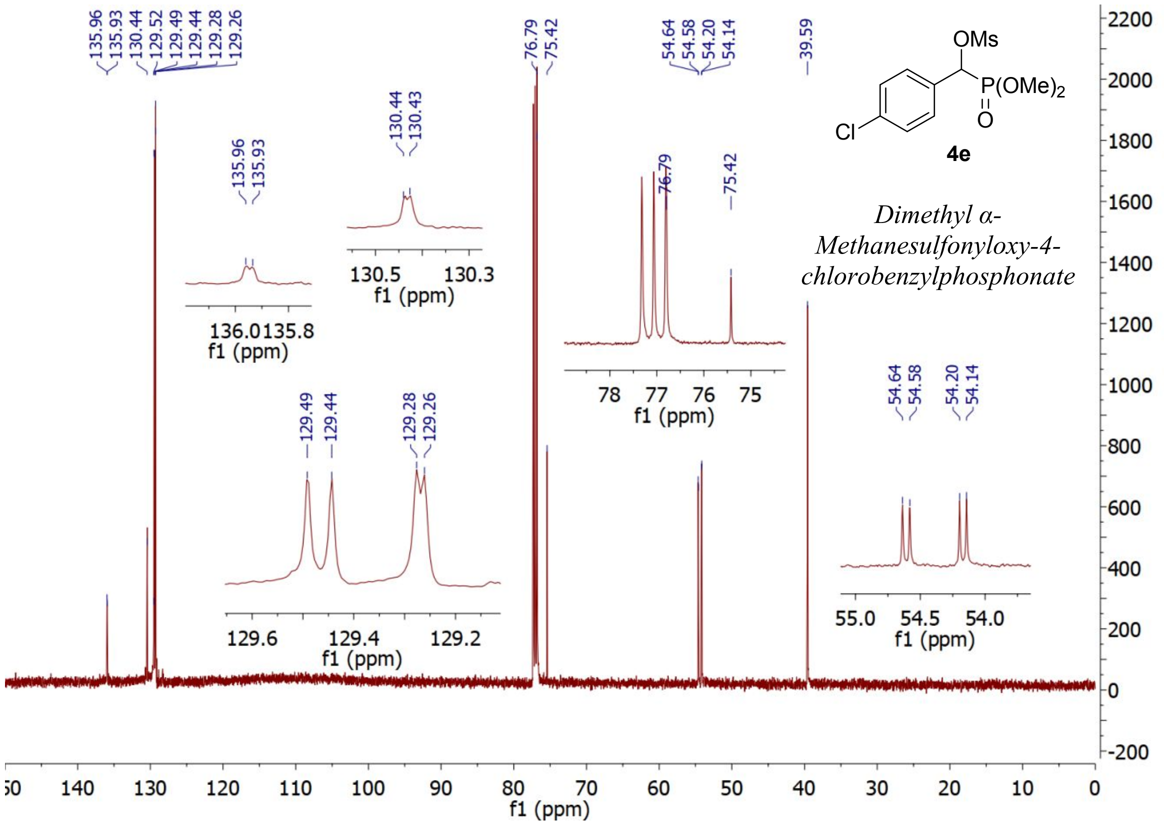

^{1}H NMR (500 MHz, $CDCl_3$) spectra for 4e

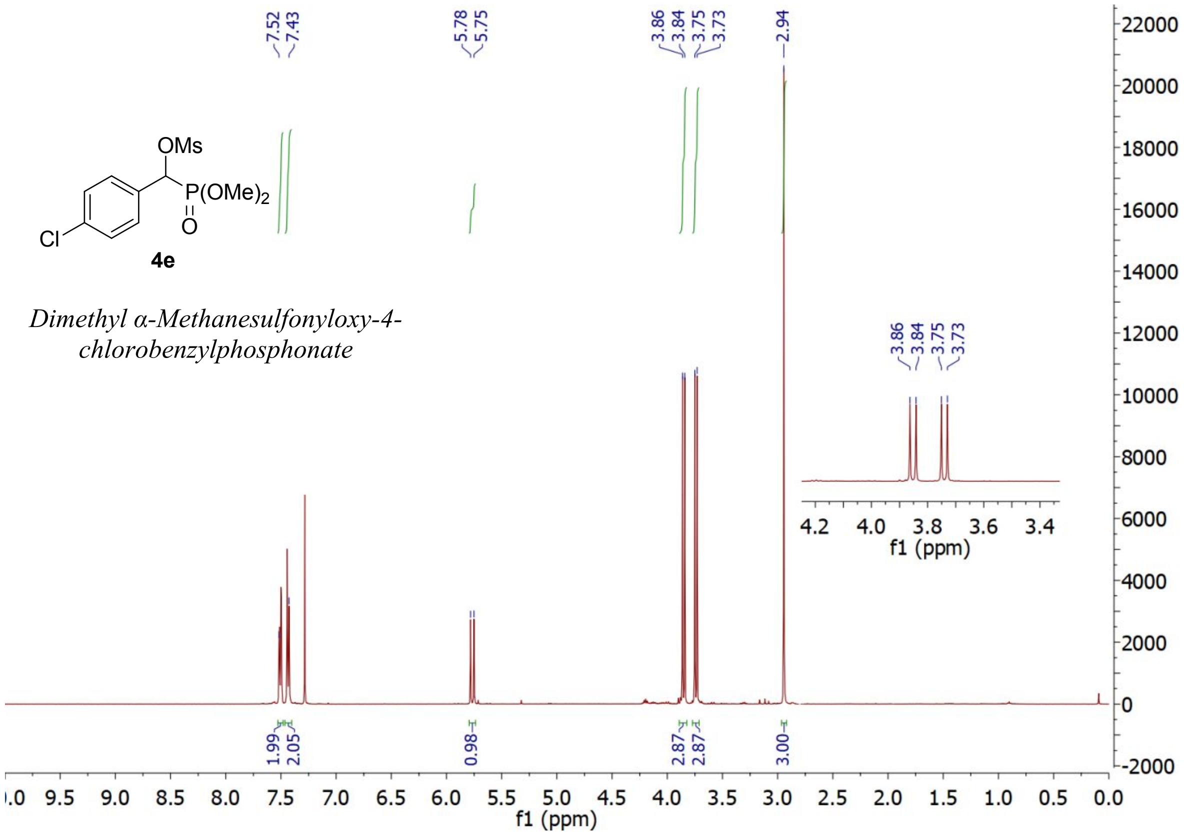

^{31}P {^{1}H} NMR (202 MHz, $CDCl_3$) spectra for 4f

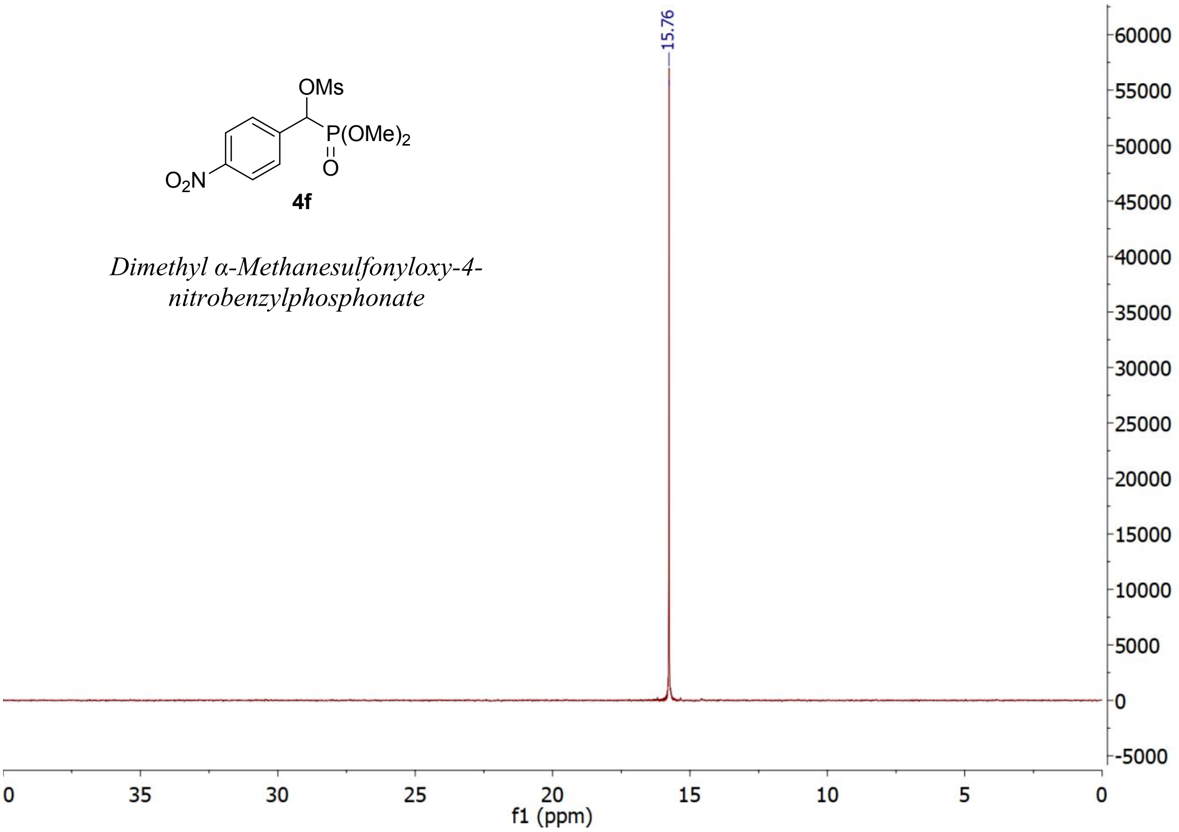

^{13}C {^{1}H} NMR (75 MHz, $CDCl_3$) spectra for 4f

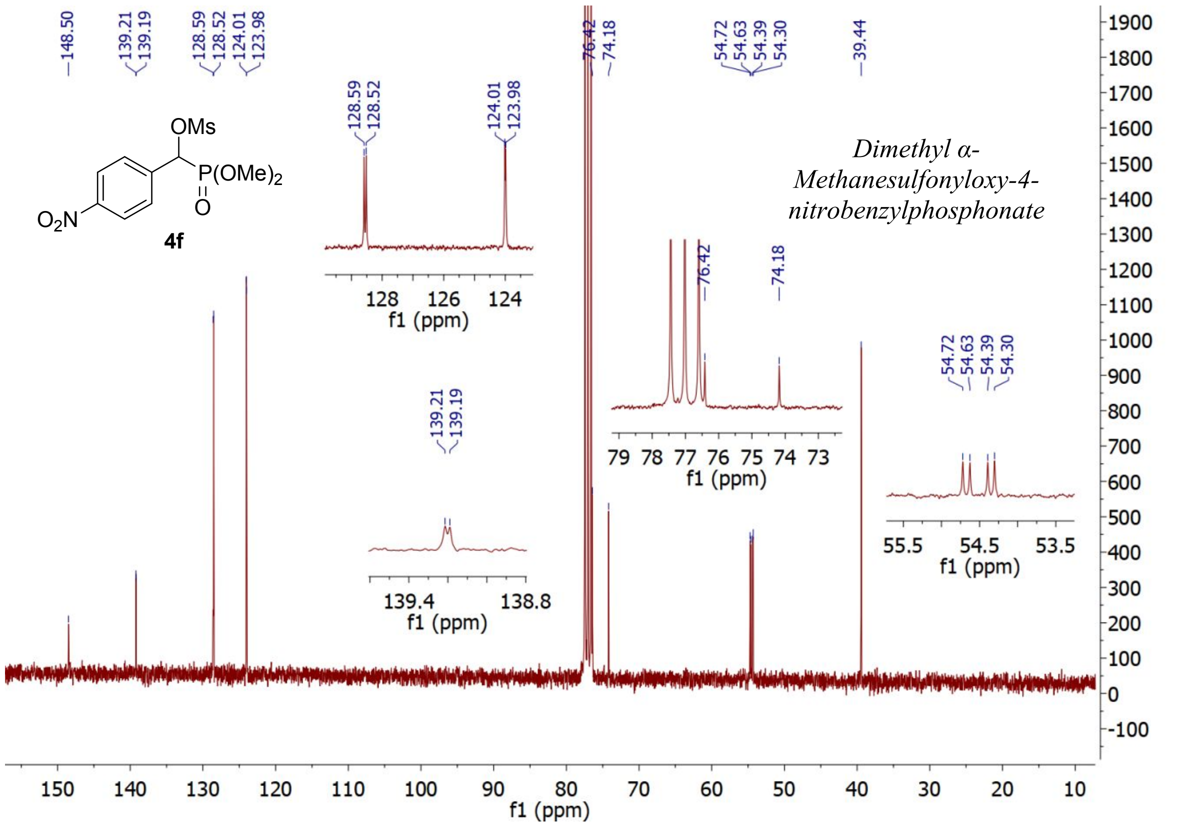

^{1}H NMR (500 MHz, $CDCl_3$) spectra for 4f

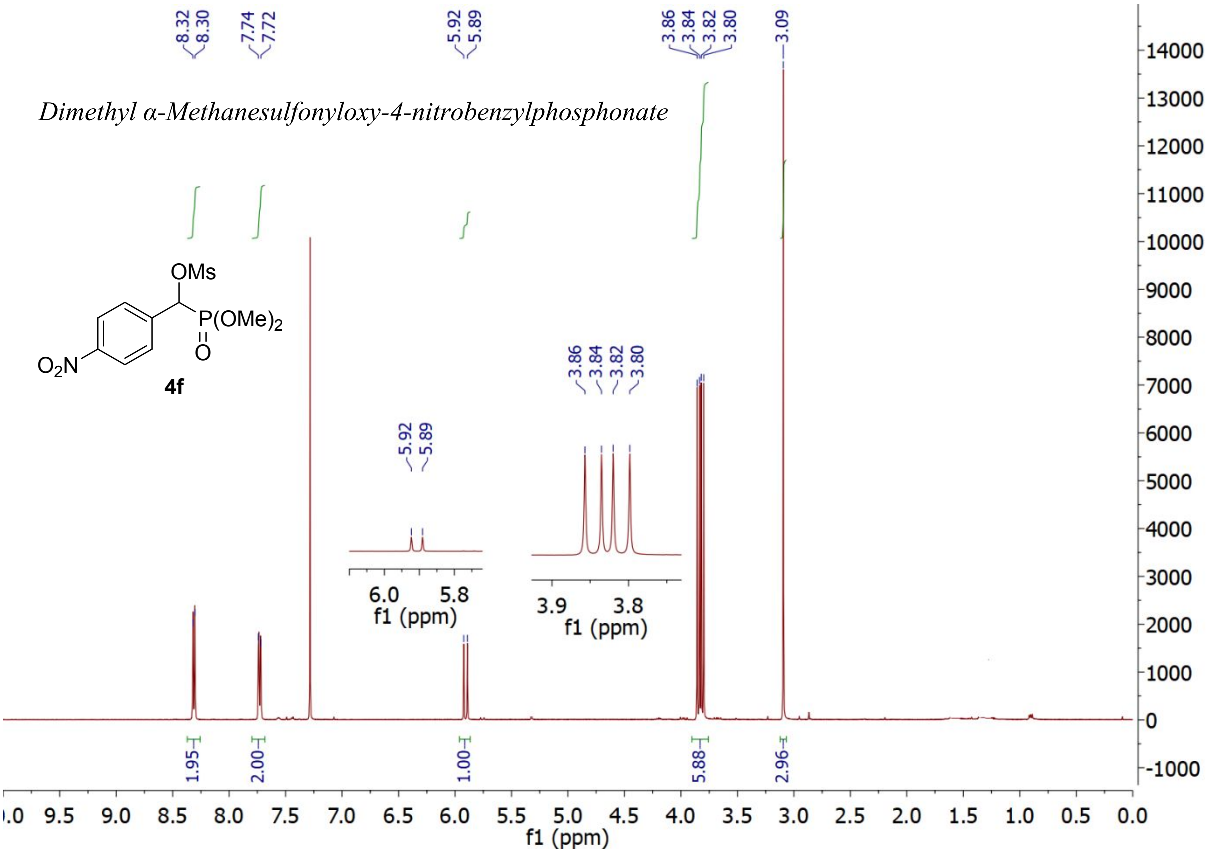

^{31}P {^{1}H} NMR (202 MHz, $CDCl_3$) spectra for 5a

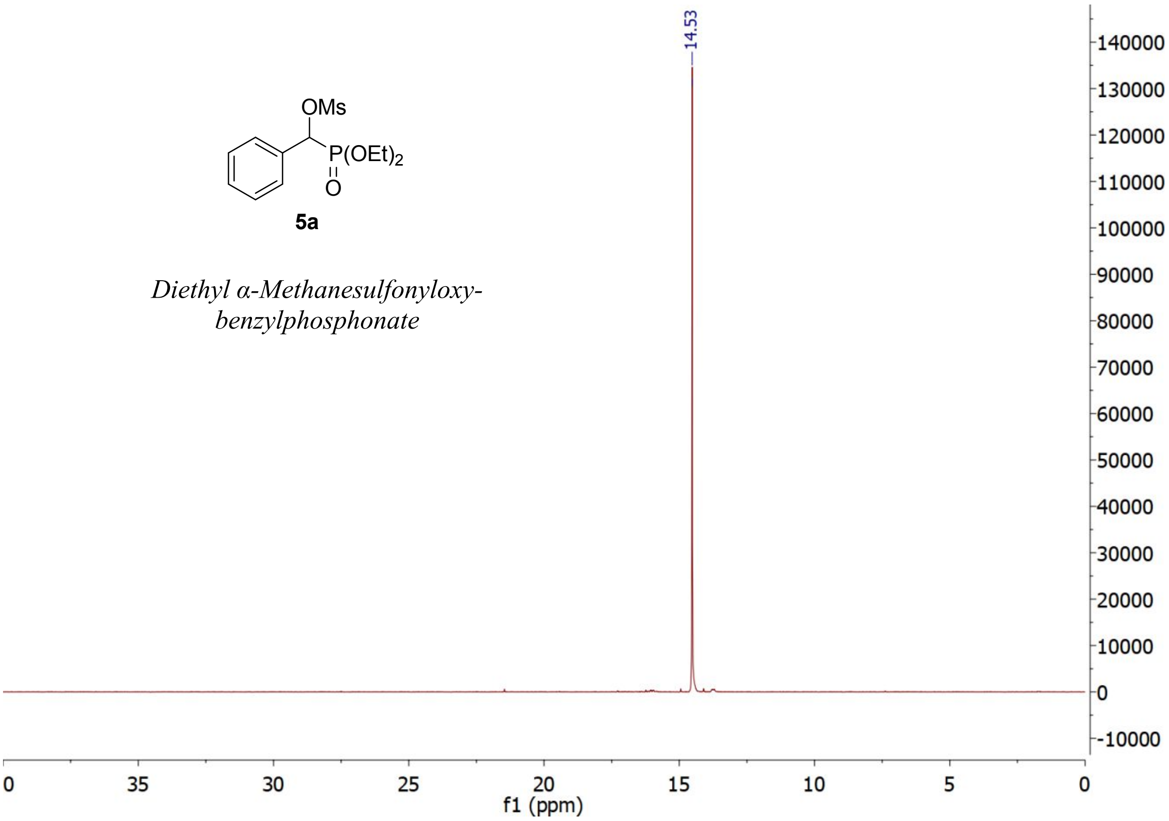

$^{13}C\ \{^{1}H\}$ NMR (126 MHz, $CDCl_3$) spectra for 5a

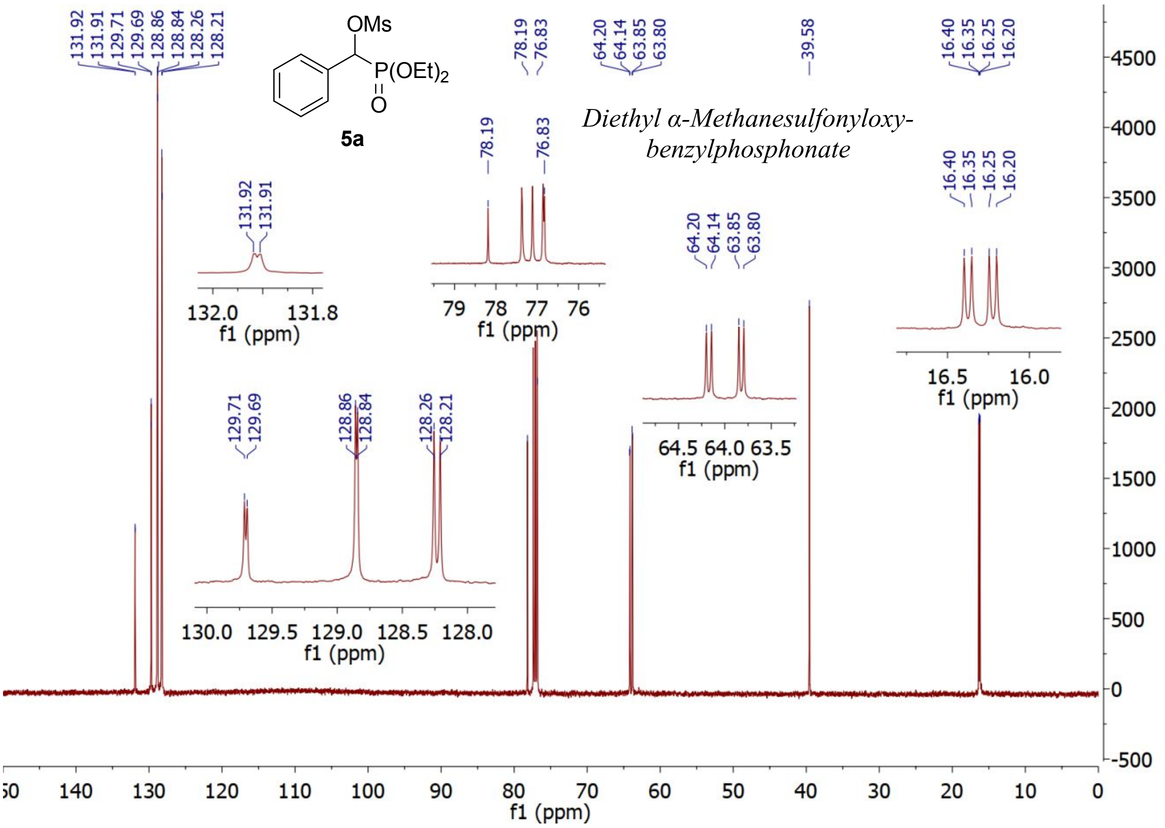

^{1}H NMR (500 MHz, $CDCl_3$) spectra for 5a

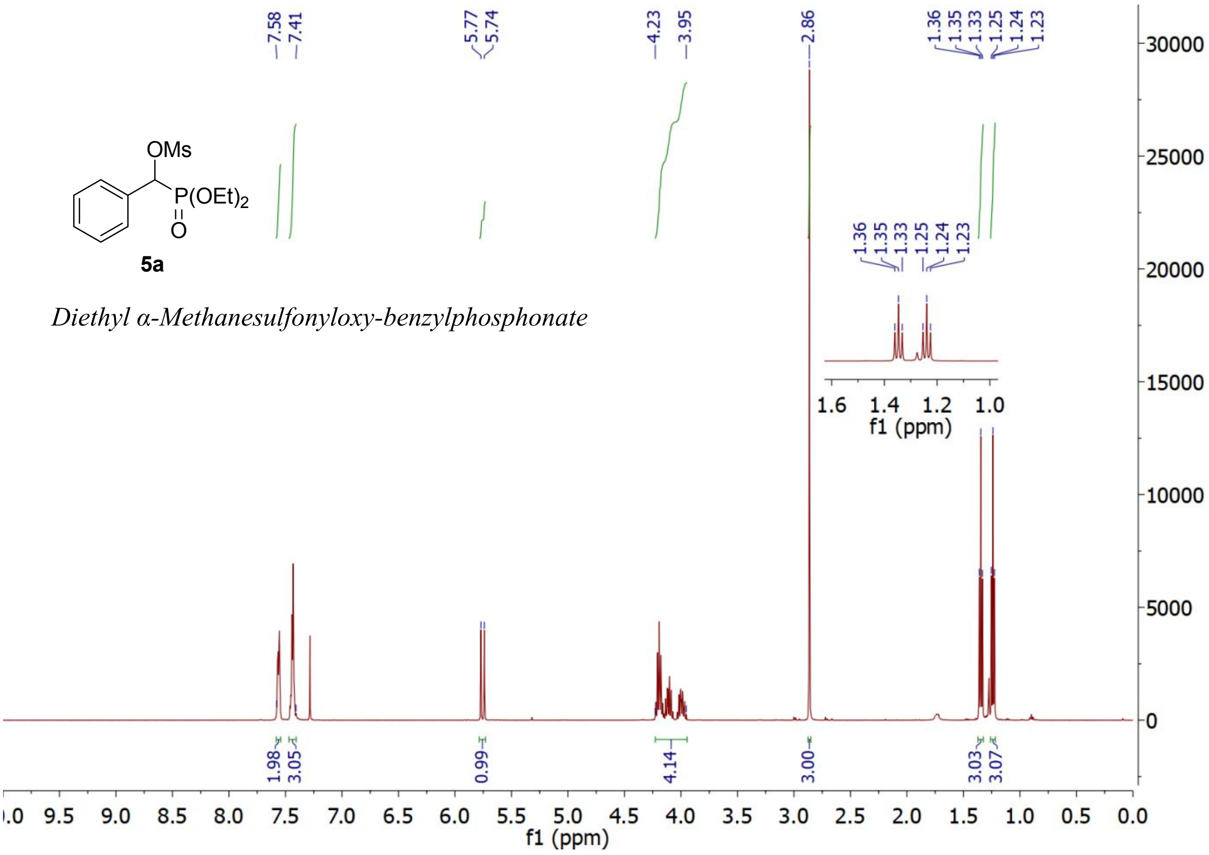

$^{31}P\ \{^{1}H\}$ NMR (202 MHz, $CDCl_3$) spectra for 5b

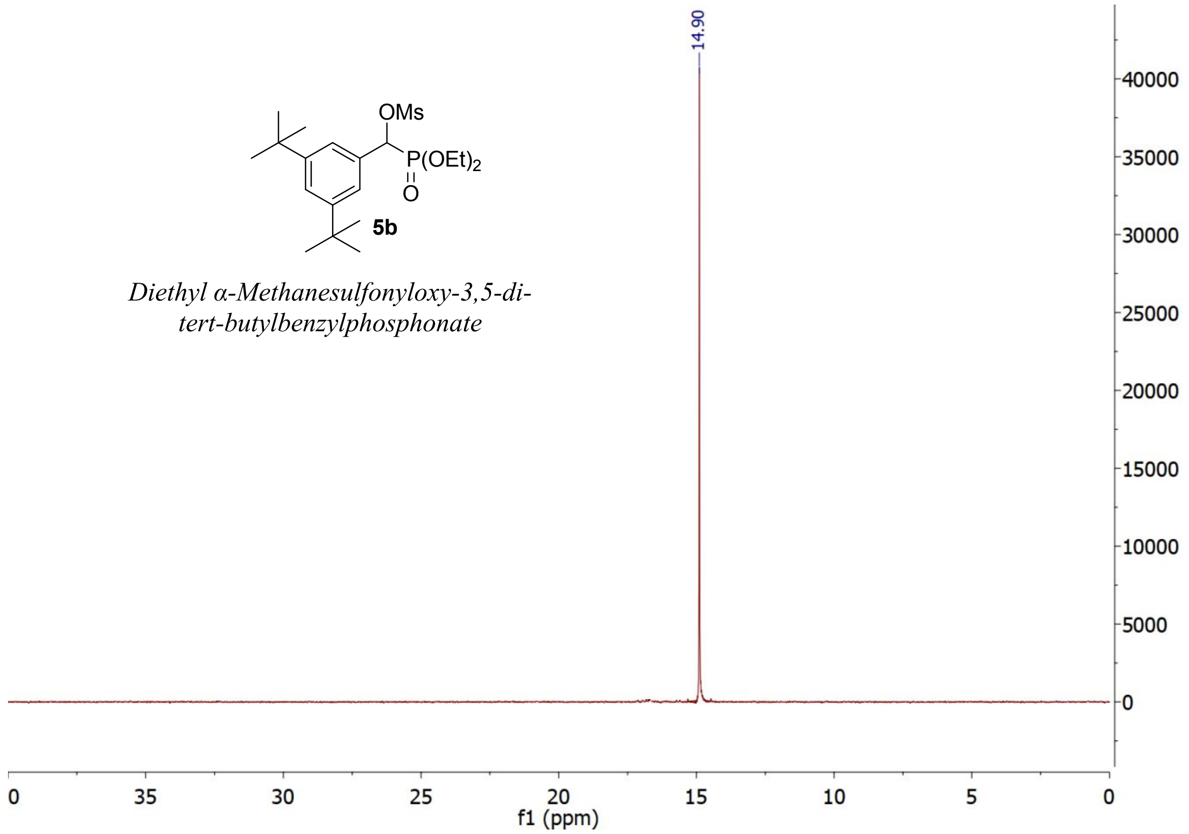

$^{13}C\ \{^{1}H\}$ NMR (126 MHz, $CDCl_3$) spectra for 5b

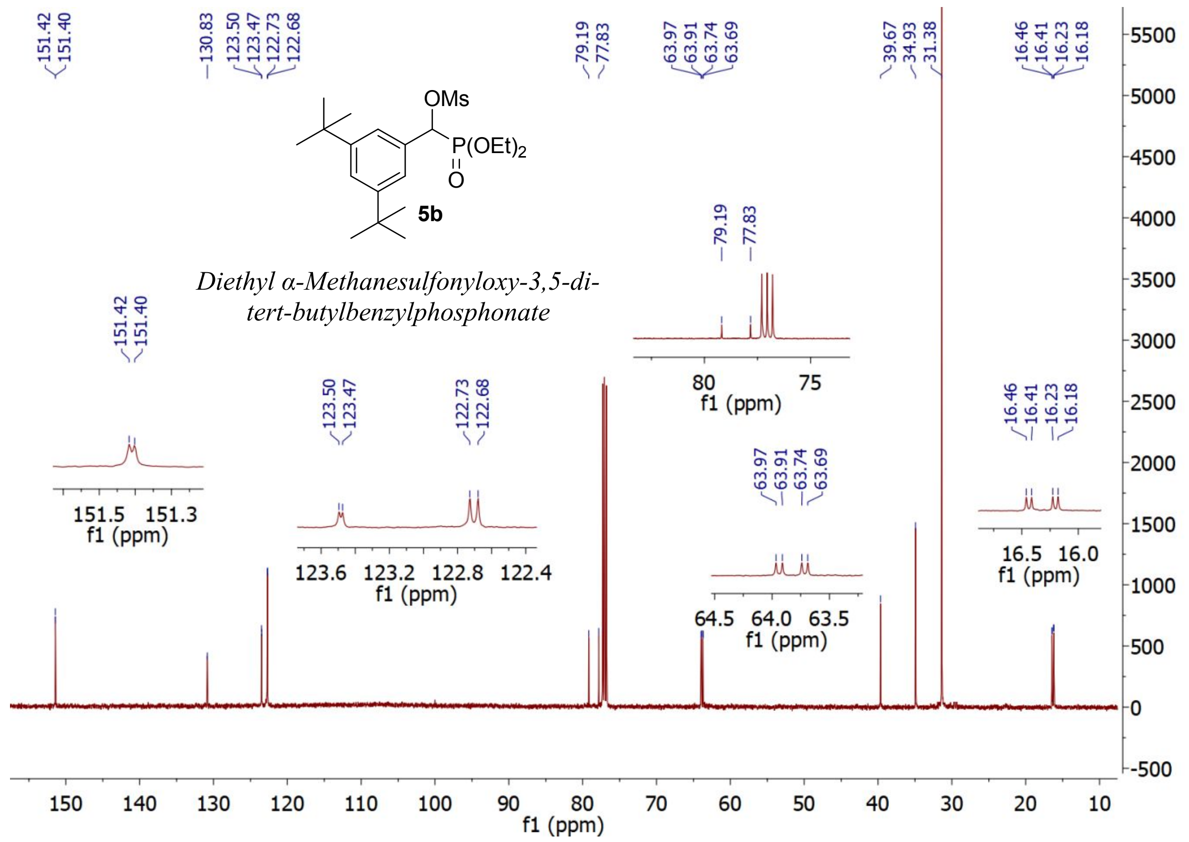

^{1}H NMR (500 MHz, $CDCl_3$) spectra for 5b

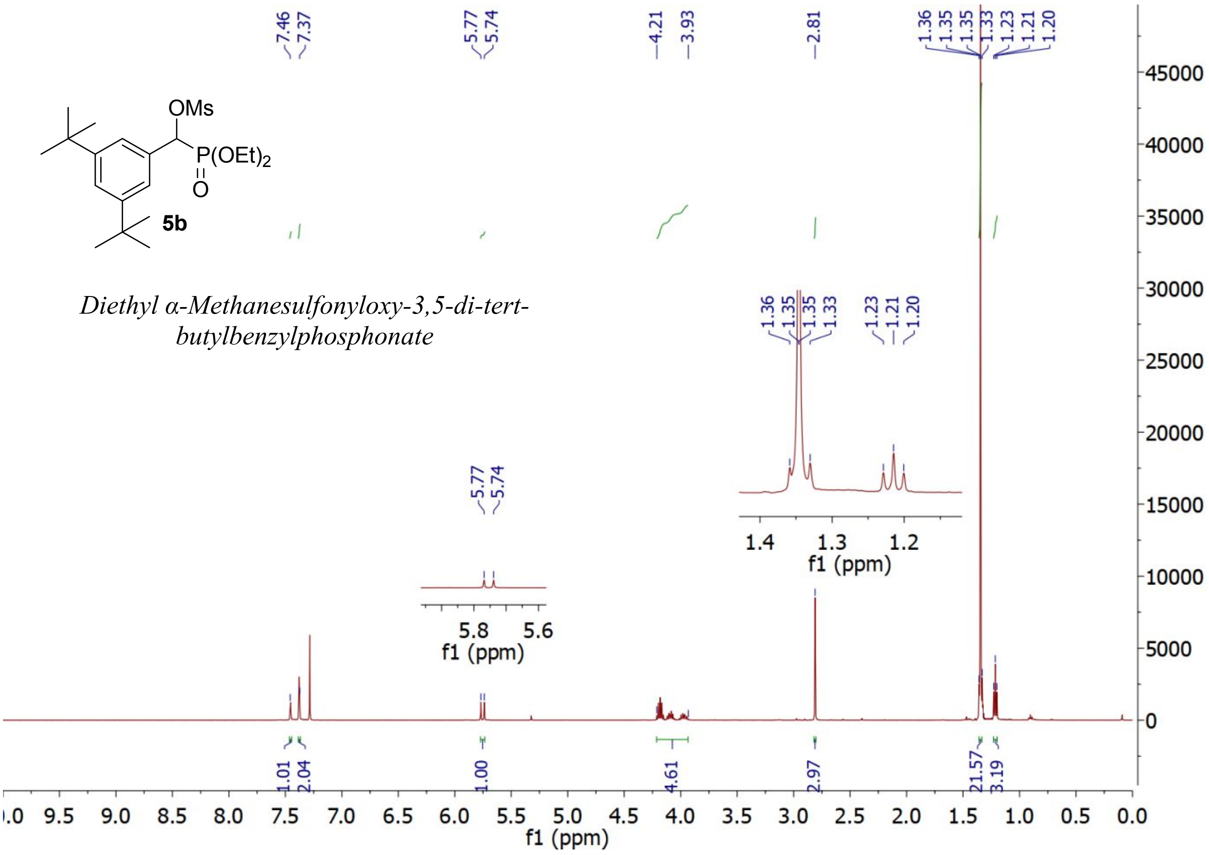

^{31}P {^{1}H} NMR (500 MHz, $CDCl_3$) spectra for 5c

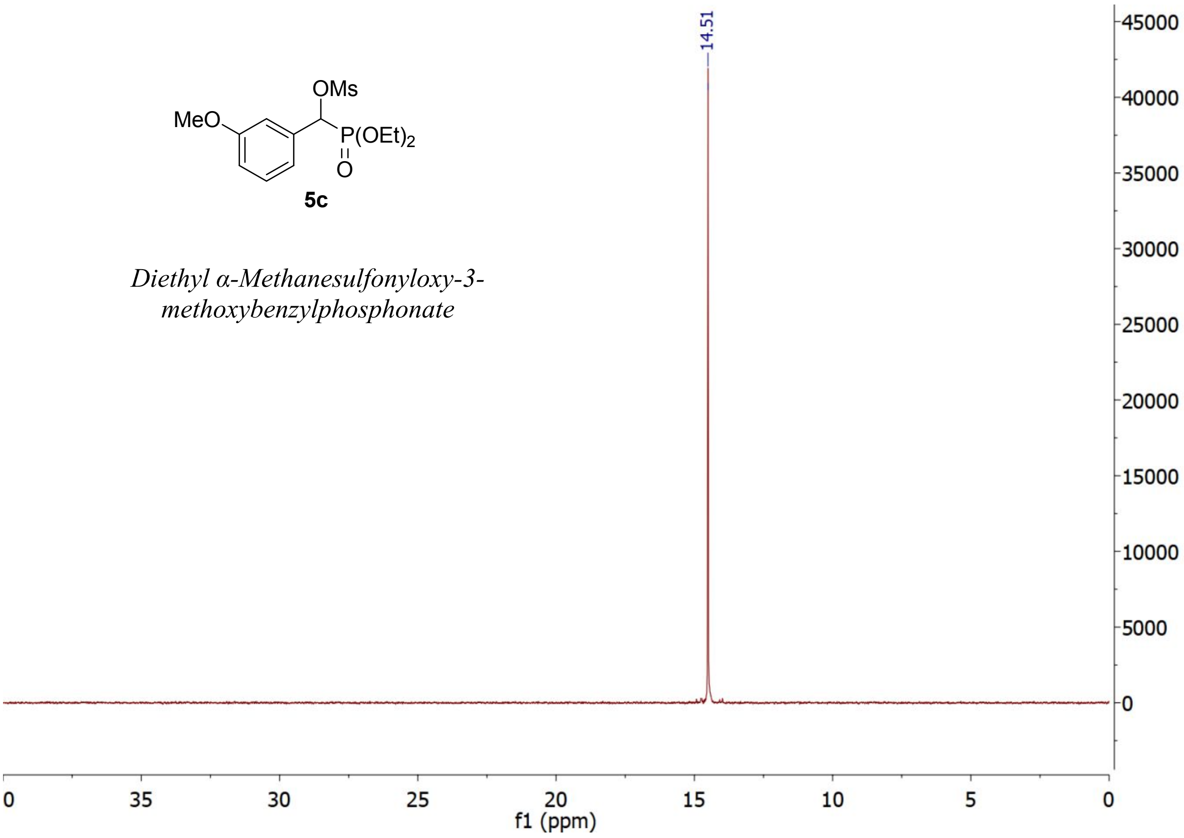

^{13}C {^{1}H} NMR (126 MHz, $CDCl_3$) spectra for 5c

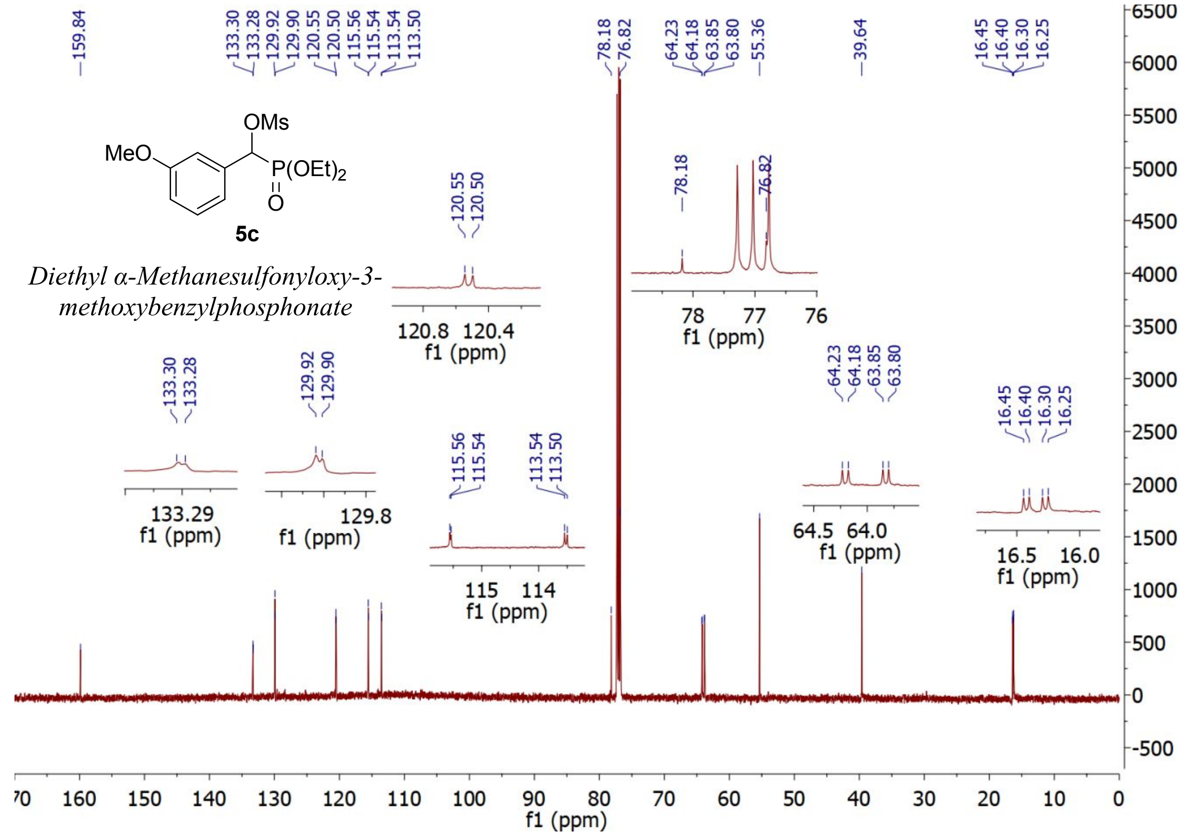

^{1}H NMR (500 MHz, $CDCl_3$) spectra for 5c

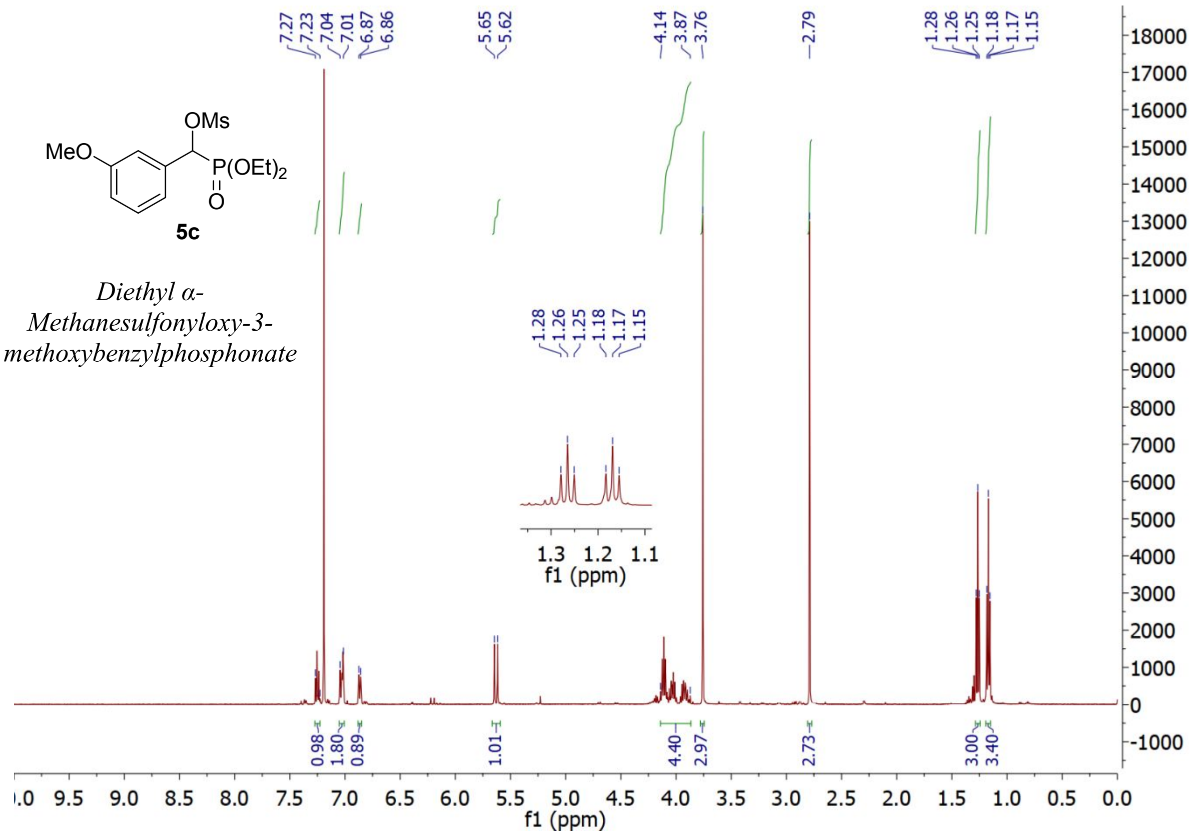

^{31}P {^{1}H} NMR (202 MHz, $CDCl_3$) spectra for 5d

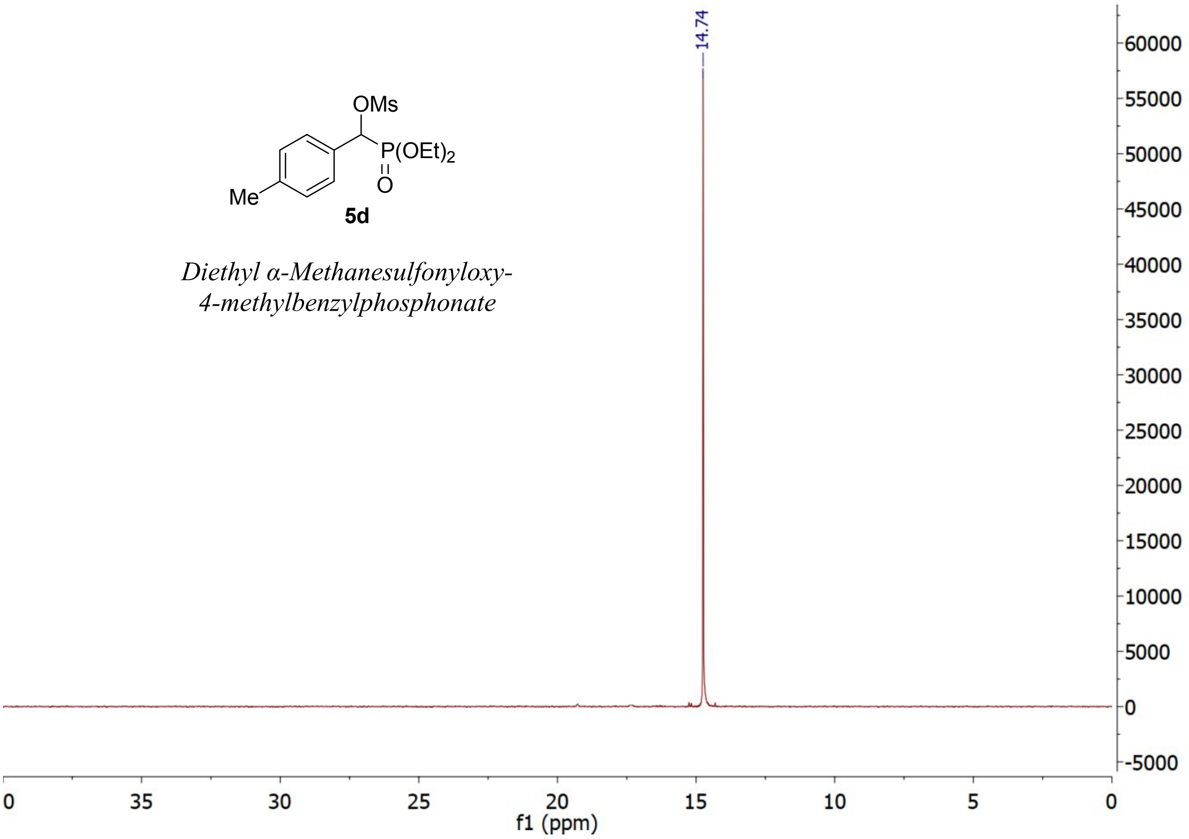

^{13}C {^{1}H} NMR (126 MHz, $CDCl_3$) spectra for 5d

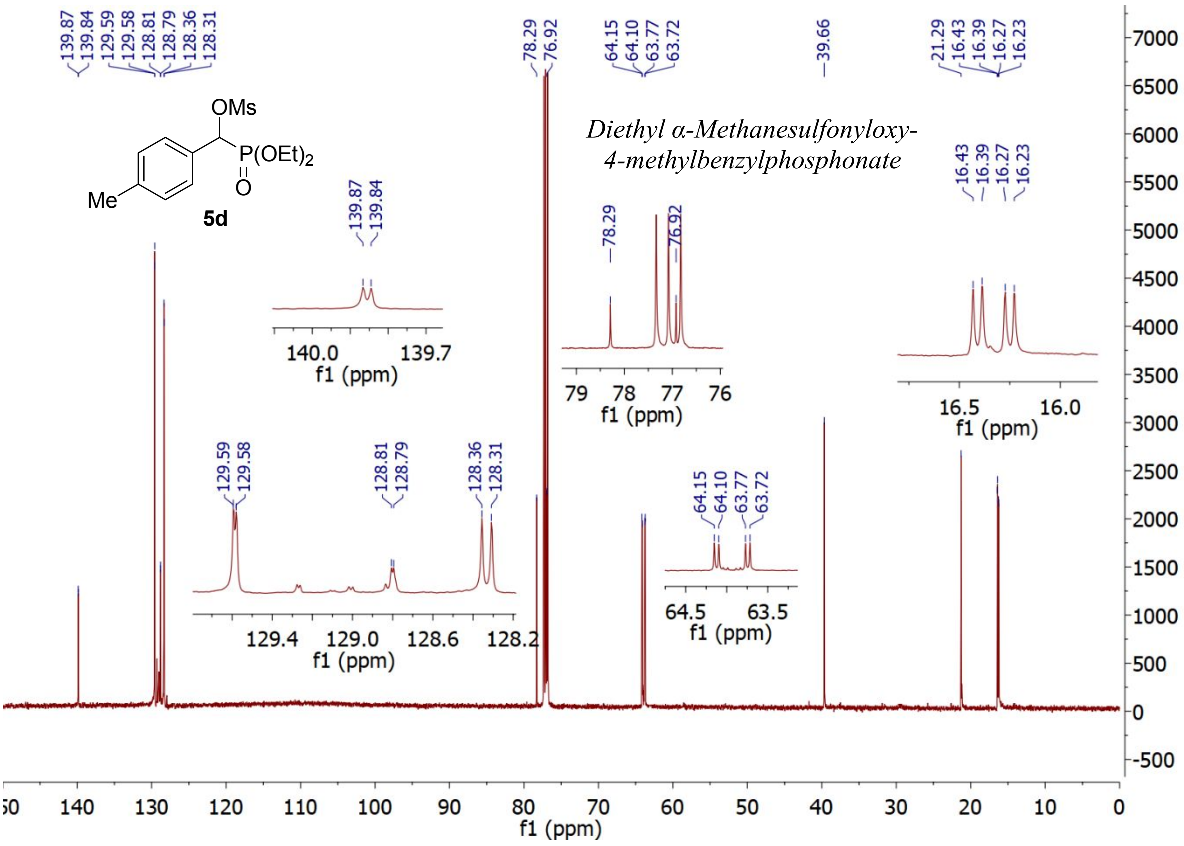

^{1}H NMR (500 MHz, $CDCl_3$) spectra for 5d

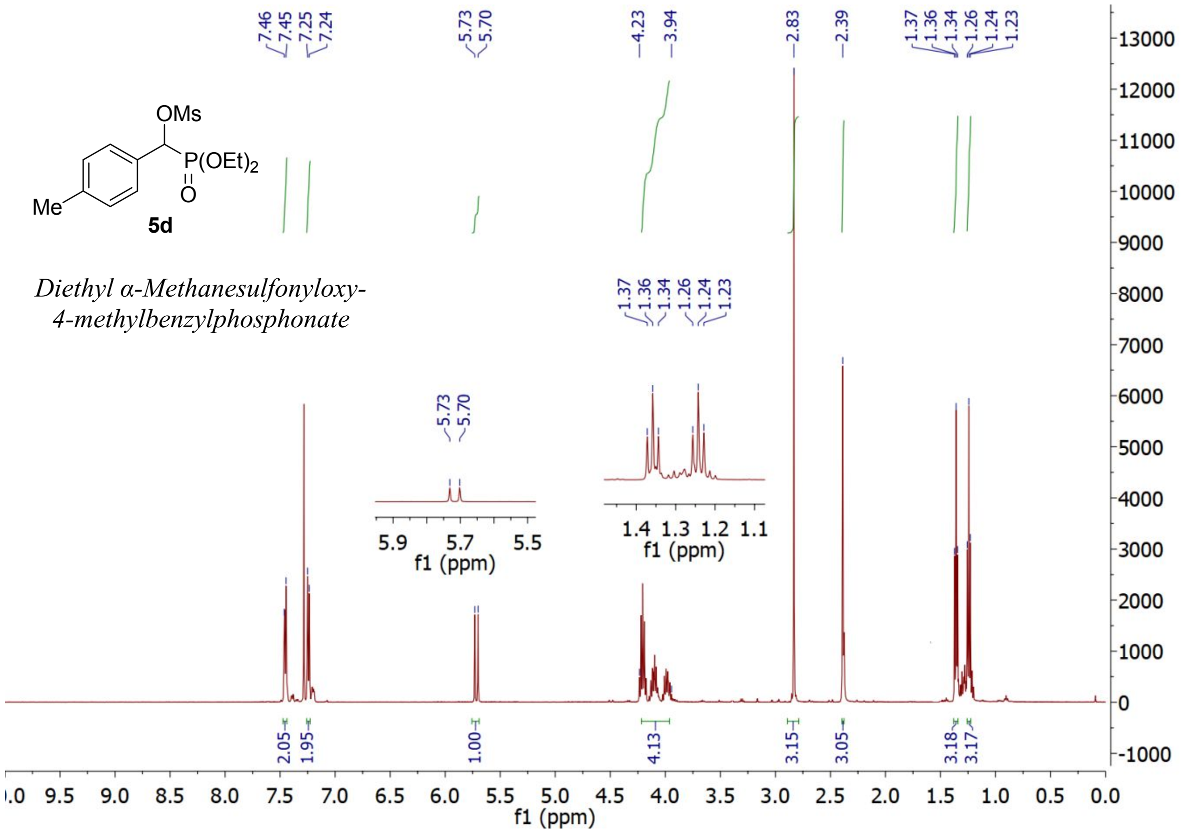

^{31}P {^{1}H} NMR (202 MHz, $CDCl_3$) spectra for 5e

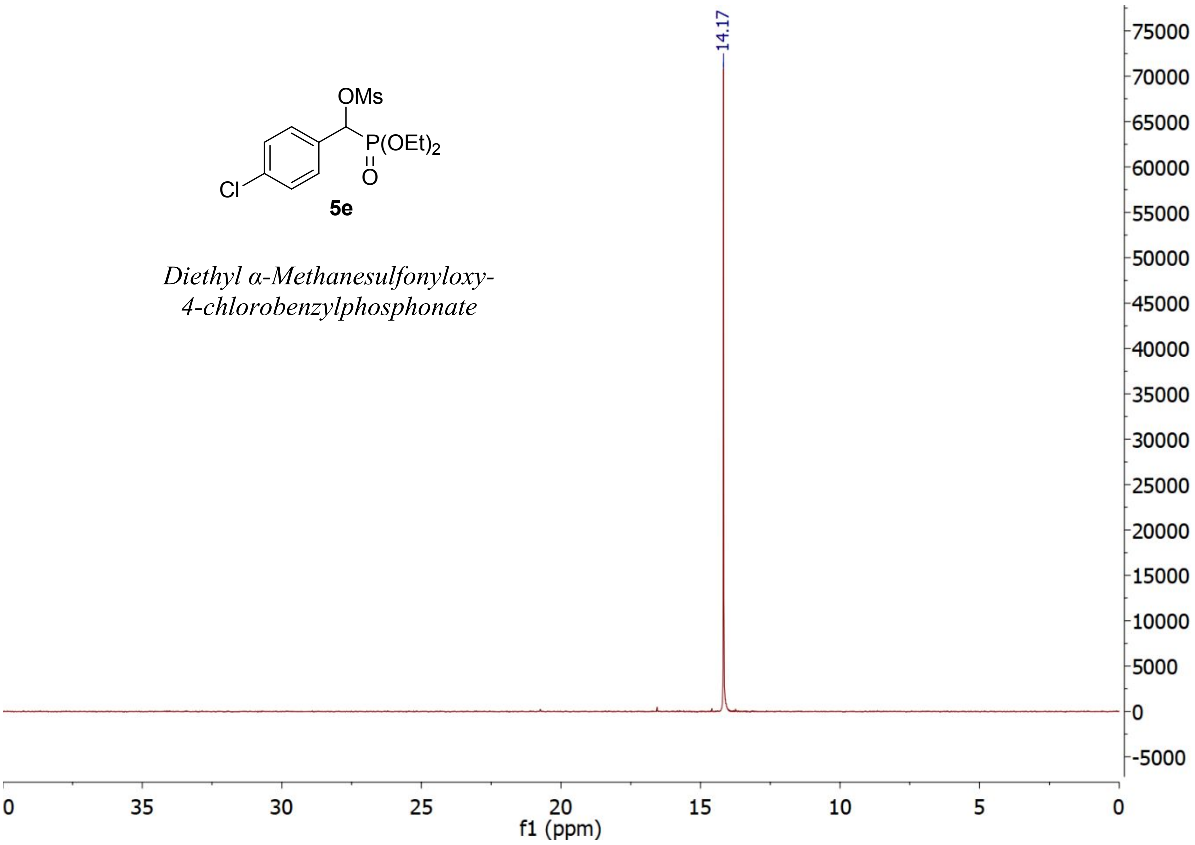

$^{13}C\ \{^1H\}$ NMR (126 MHz, $CDCl_3$) spectra for 5e

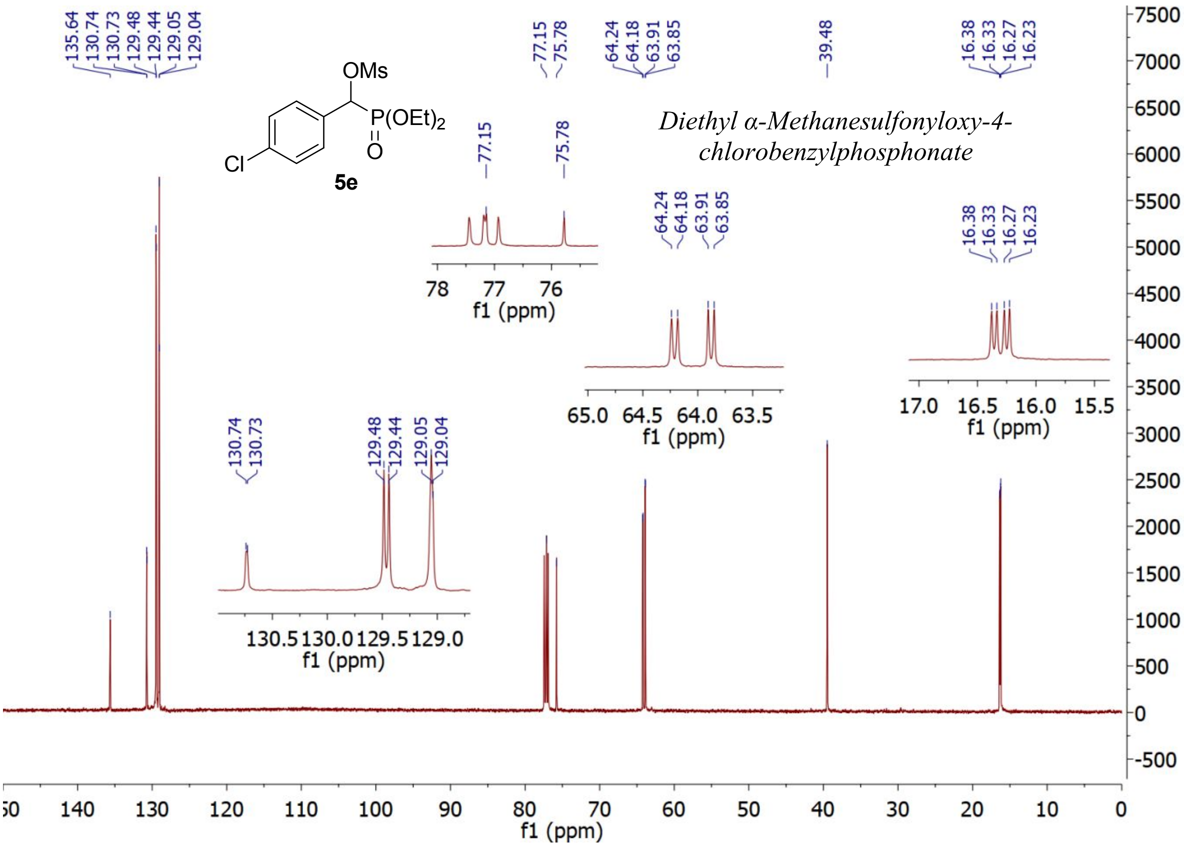

^1H NMR (500 MHz, $CDCl_3$) spectra for 5e

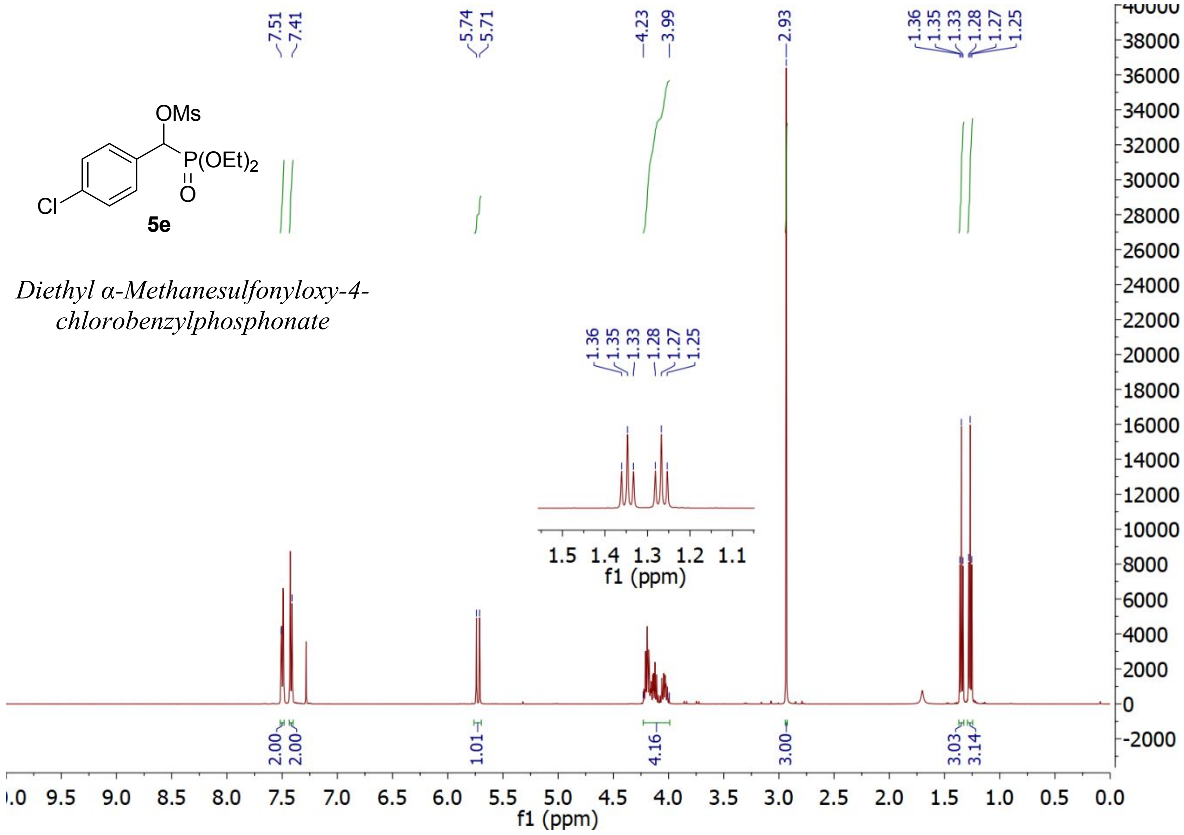

^{31}P {^{1}H} NMR (202 MHz, $CDCl_3$) spectra for 5f

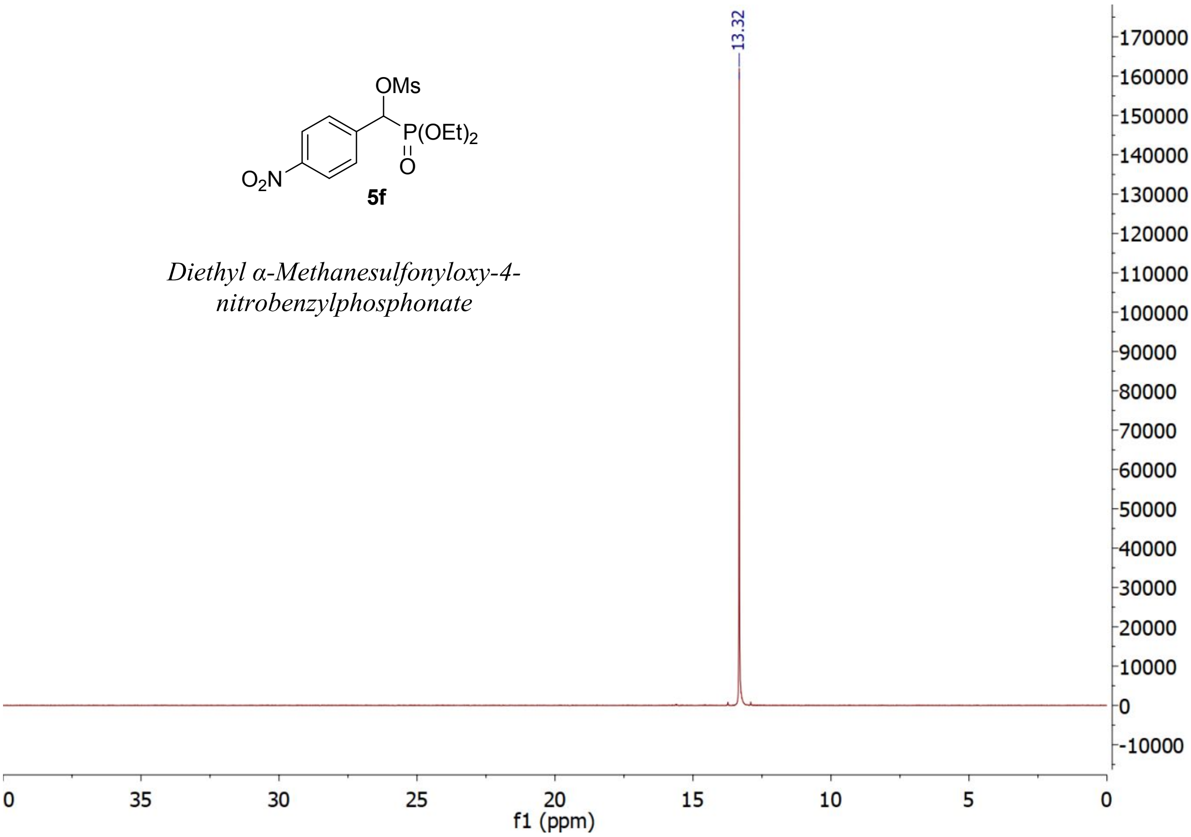

^{13}C {^{1}H} NMR (75 MHz, $CDCl_3$) spectra for 5f

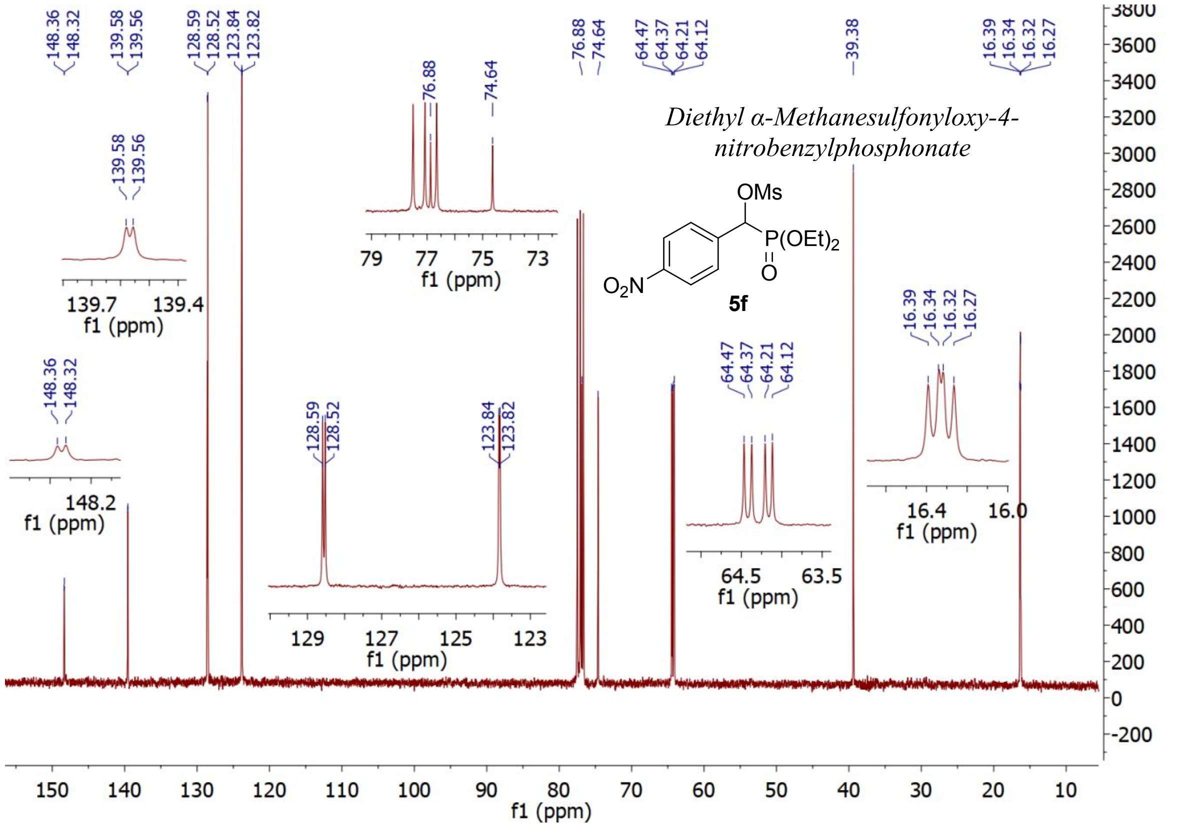

^{1}H NMR (500 MHz, $CDCl_3$) spectra for 5f

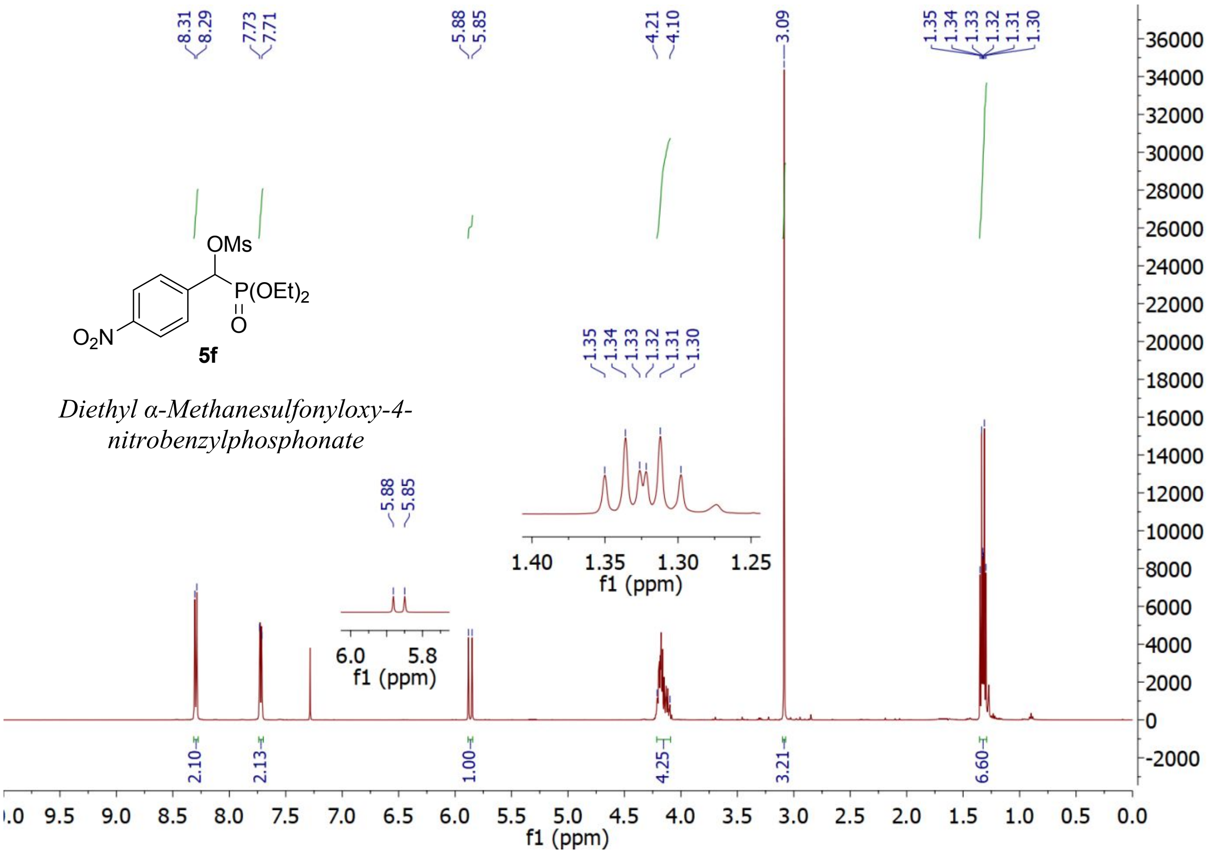

^{31}P {^{1}H} NMR (202 MHz, $CDCl_3$) spectra for 6a

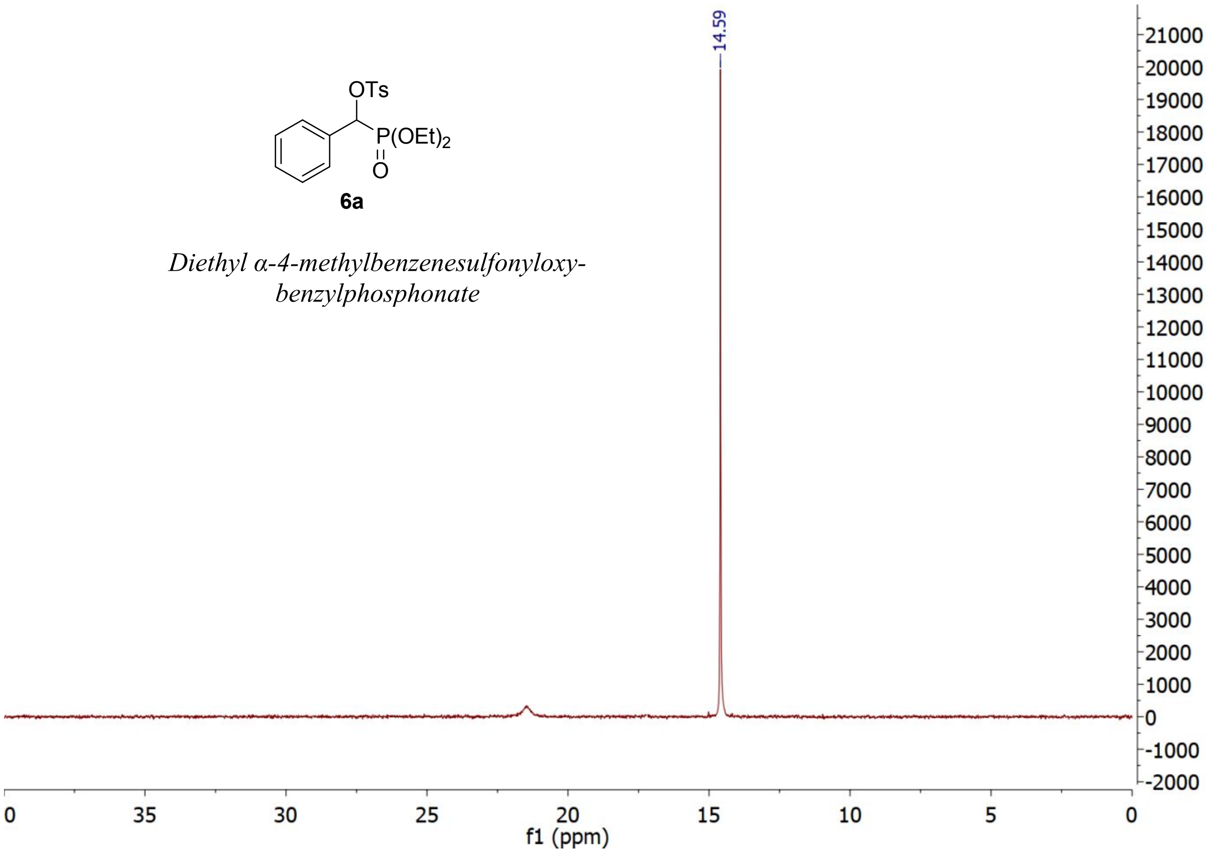

$^{31}P\ \{^{1}H\}$ NMR (202 MHz, $CDCl_3$) spectra for 6d

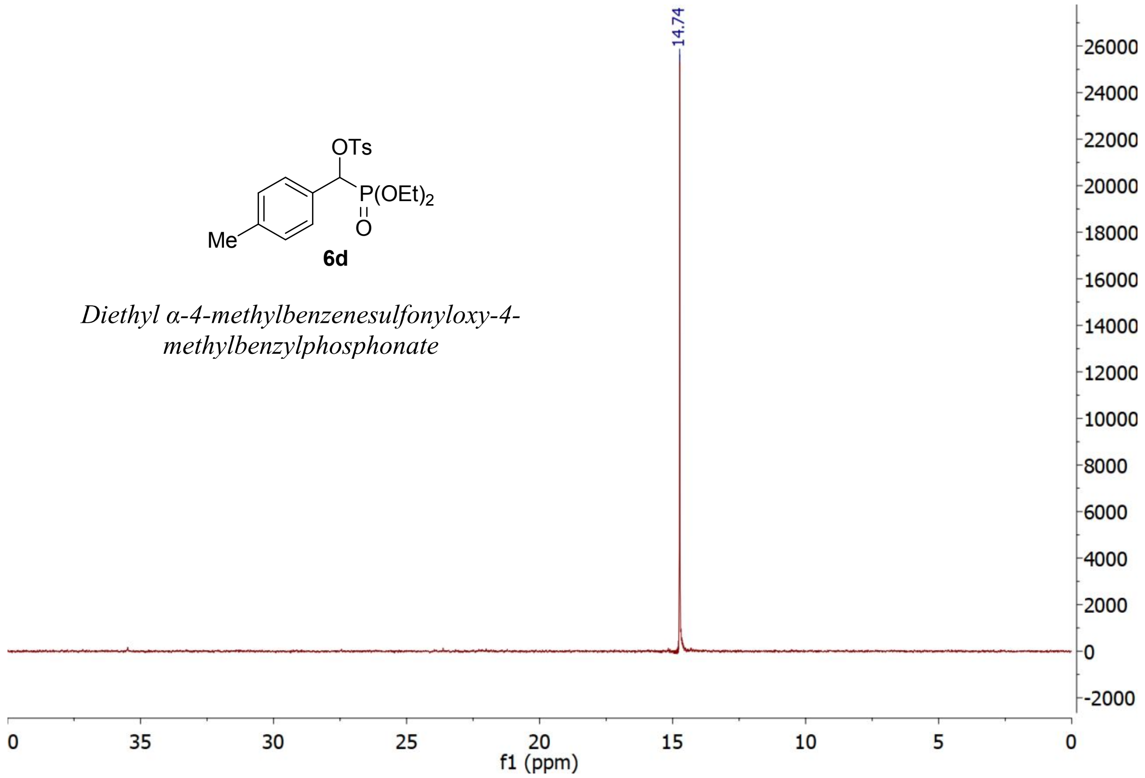

Diethyl α-4-methylbenzenesulfonyloxy-4-methylbenzylphosphonate

$^{31}P\ \{^{1}H\}$ NMR (202 MHz, $CDCl_3$) spectra for 6e

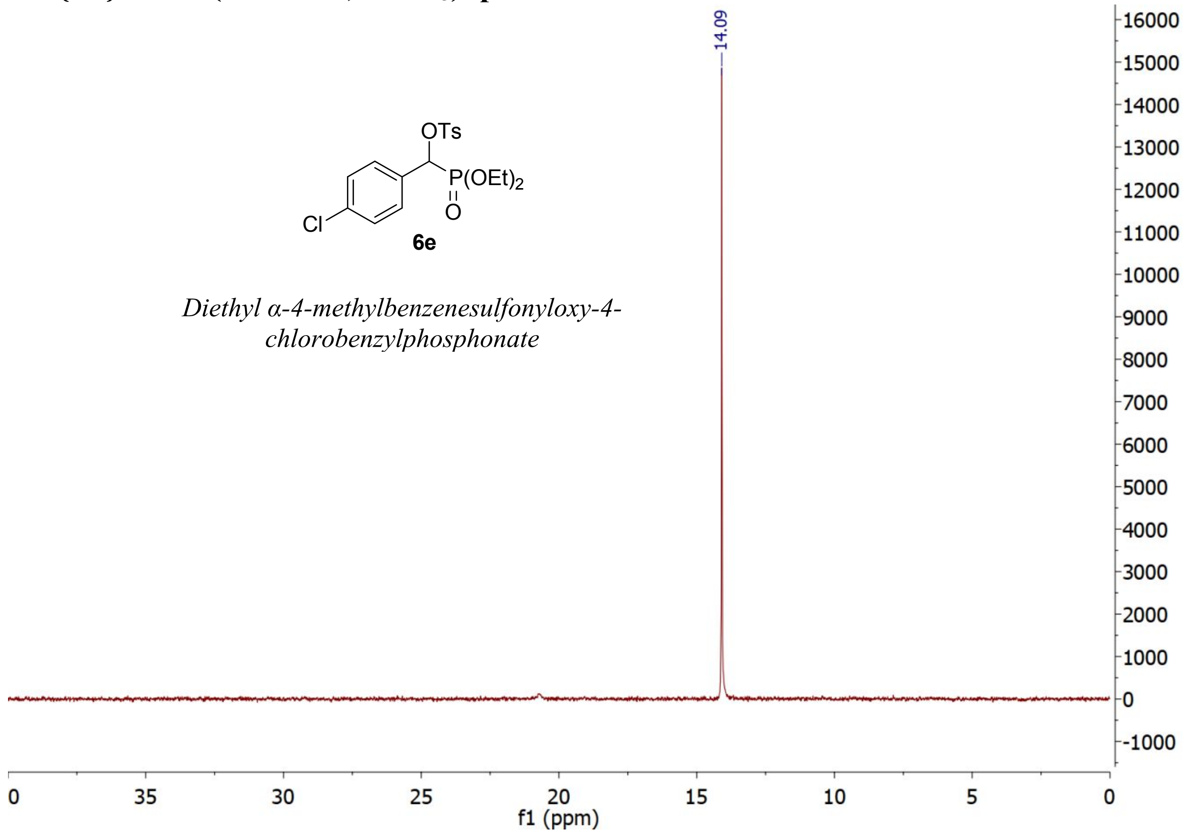

Diethyl α-4-methylbenzenesulfonyloxy-4-chlorobenzylphosphonate

^{31}P {^{1}H} NMR (202 MHz, CDCl$_3$) spectra for 8g

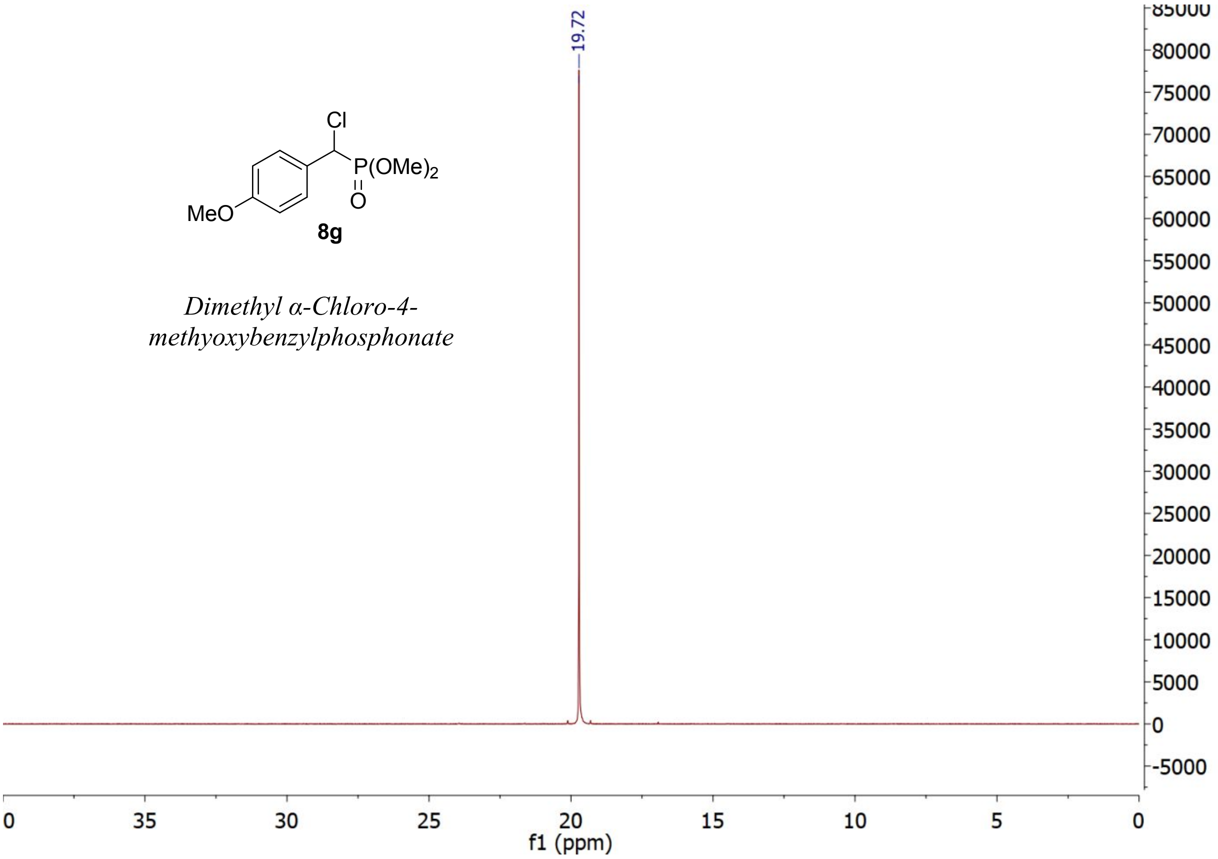

^{13}C {^{1}H} NMR (126 MHz, CDCl$_3$) spectra for 8g

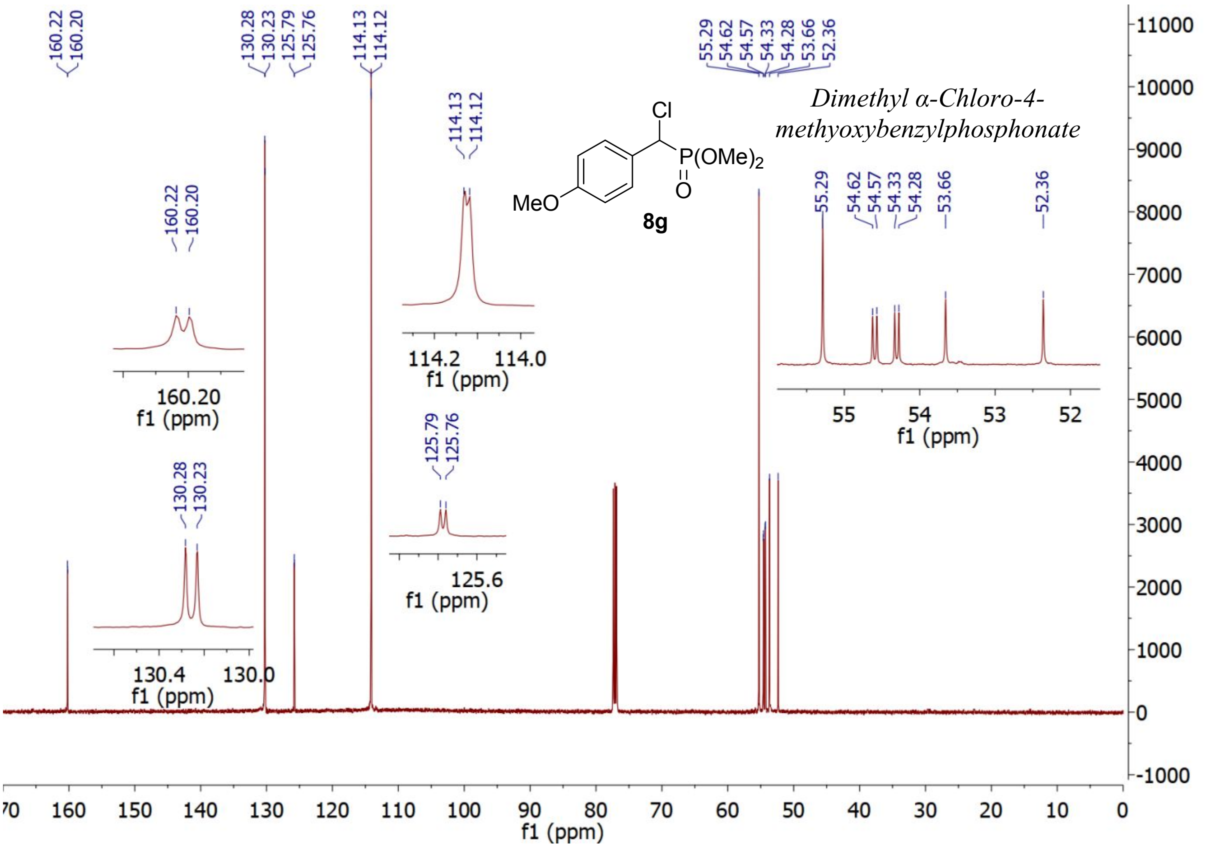

^{1}H NMR (500 MHz, $CDCl_3$) spectra for 8g

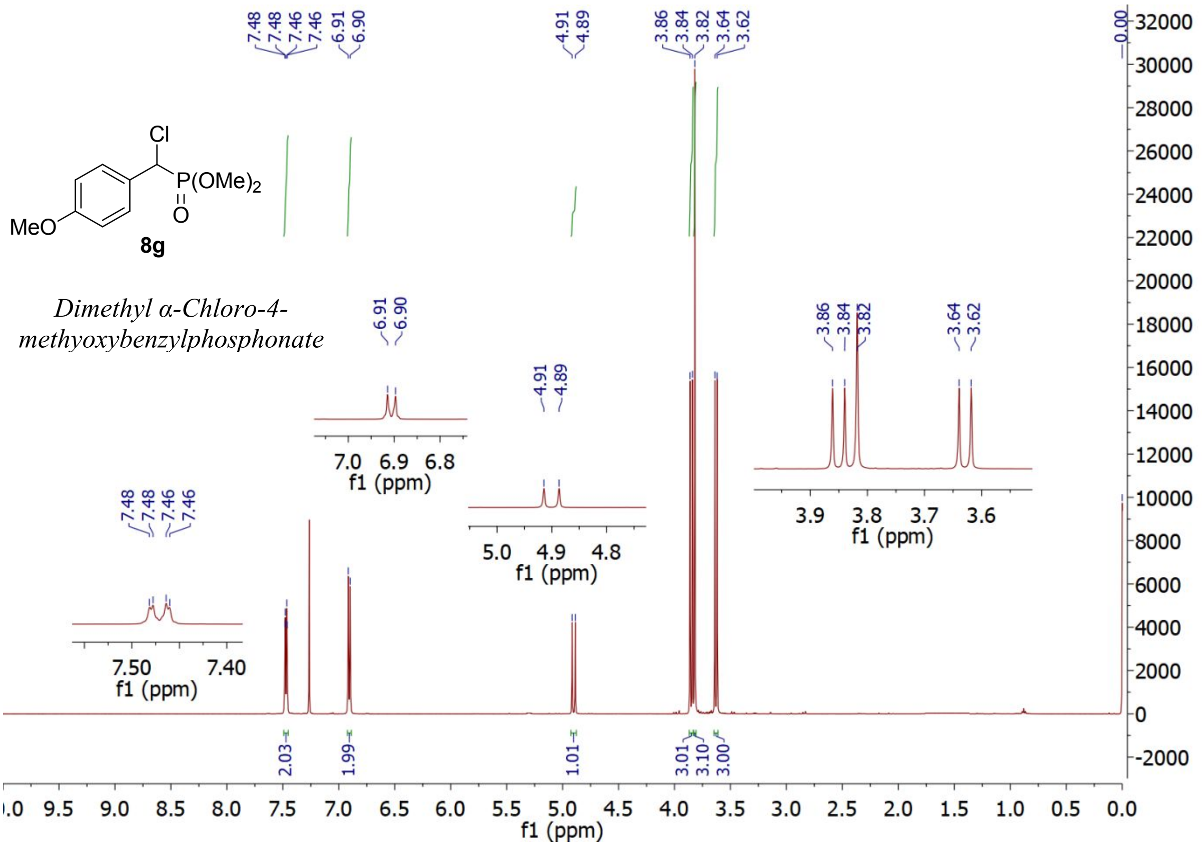

^{31}P {^{1}H} NMR (202 MHz, $CDCl_3$) spectra for 9g

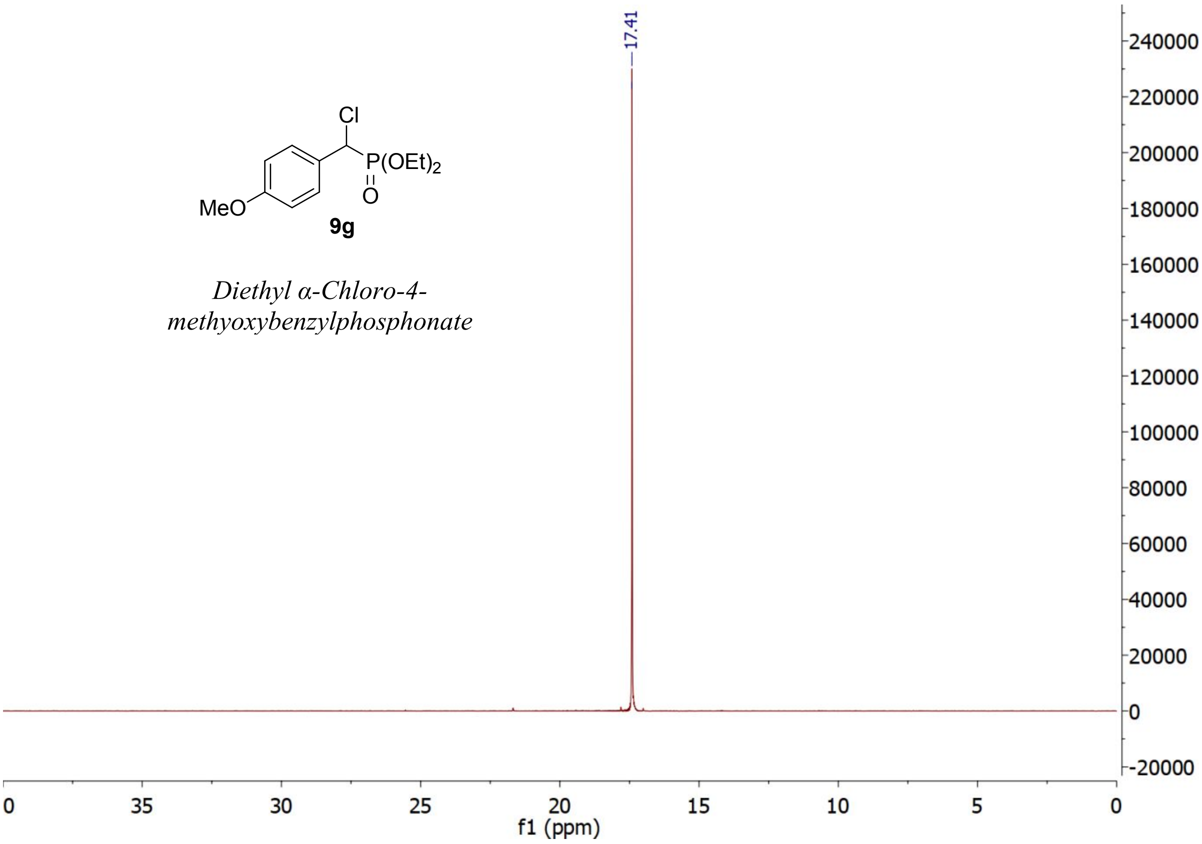

^{31}P {^{1}H} NMR (202 MHz, $CDCl_3$) spectra for 9h

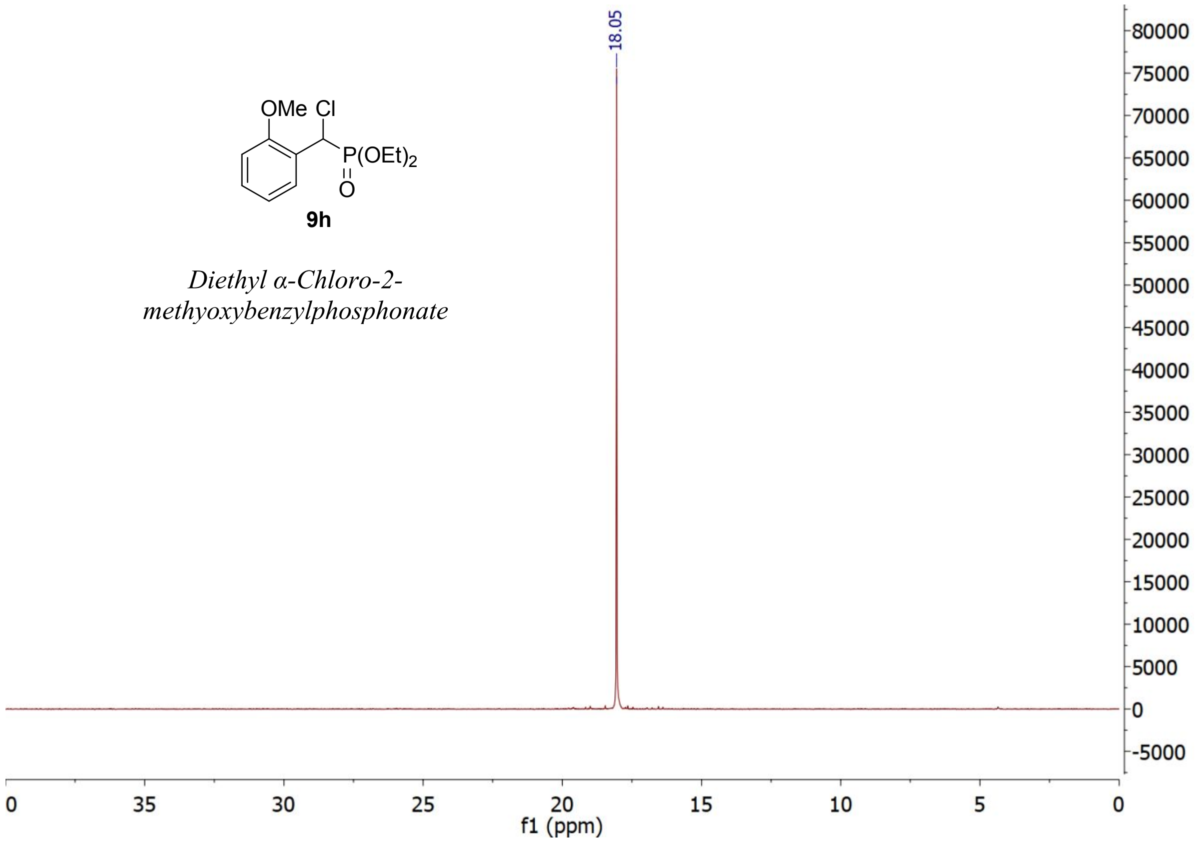

^{13}C {^{1}H} NMR (126 MHz, $CDCl_3$) spectra for 9h

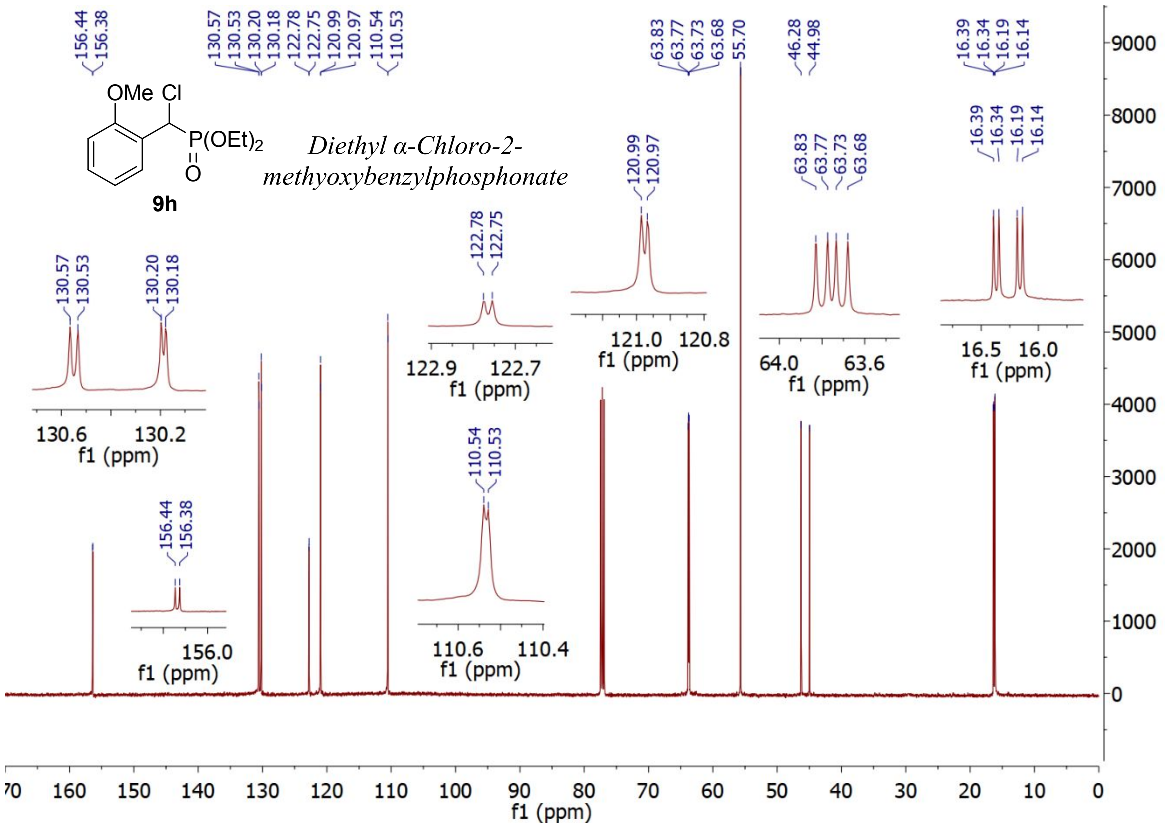

^{1}H NMR (500 MHz, CDCl$_3$) spectra for 9h

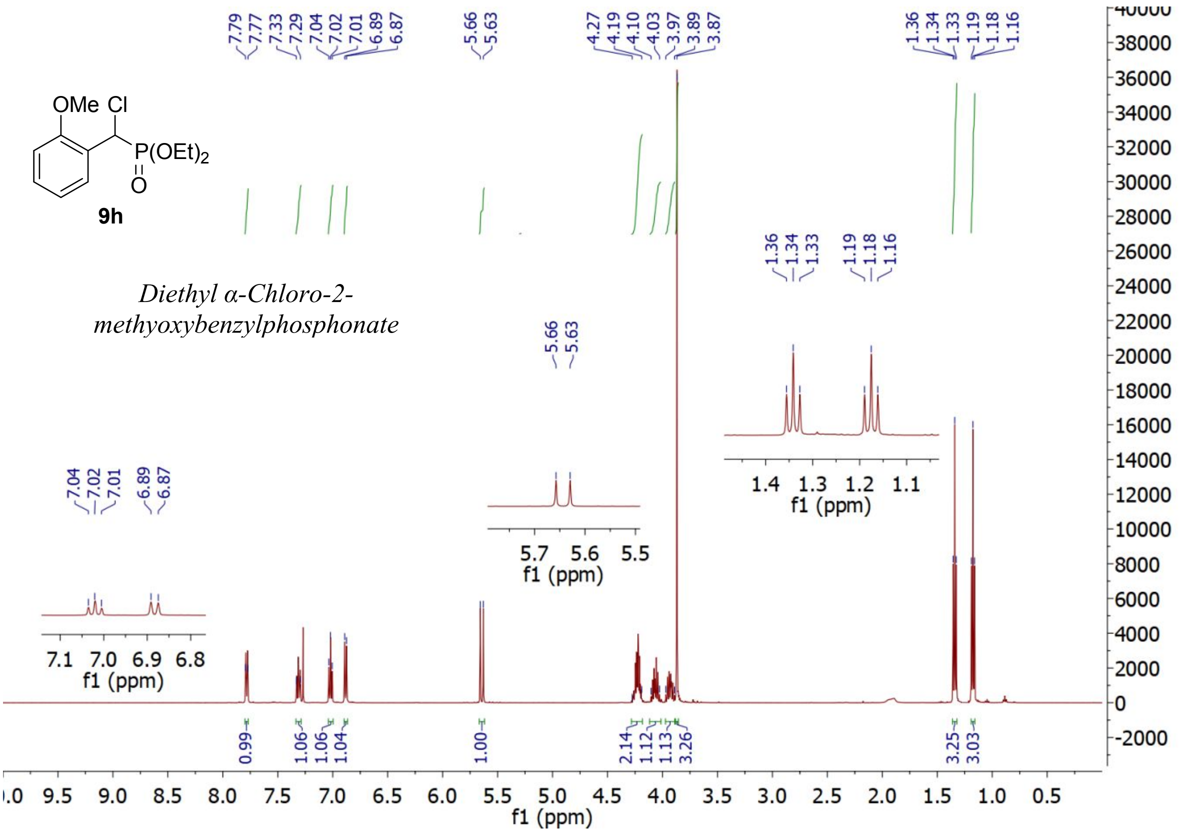

Diethyl α-Chloro-2-methyoxybenzylphosphonate

^{31}P {^{1}H} NMR (202 MHz, CDCl$_3$) spectra for 14a

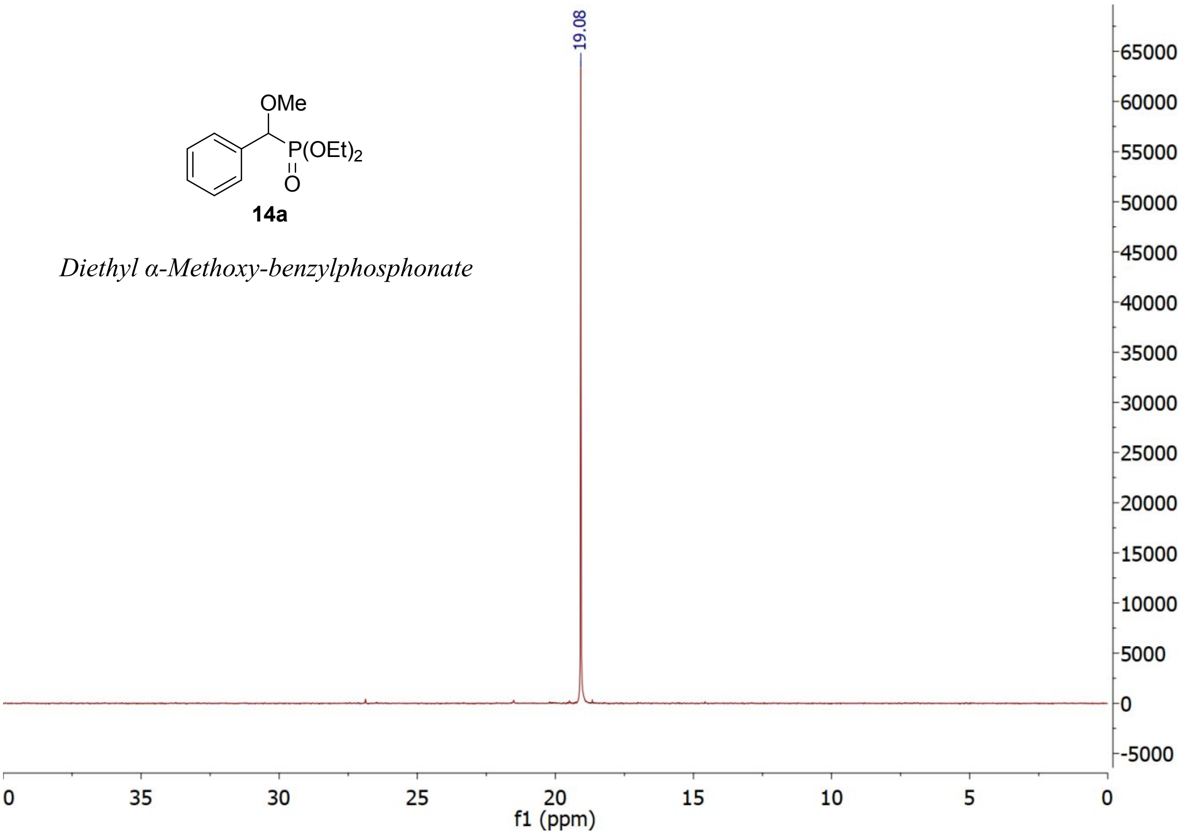

Diethyl α-Methoxy-benzylphosphonate

^{31}P {^{1}H} NMR (122 MHz, $CDCl_3$) spectra for 14d

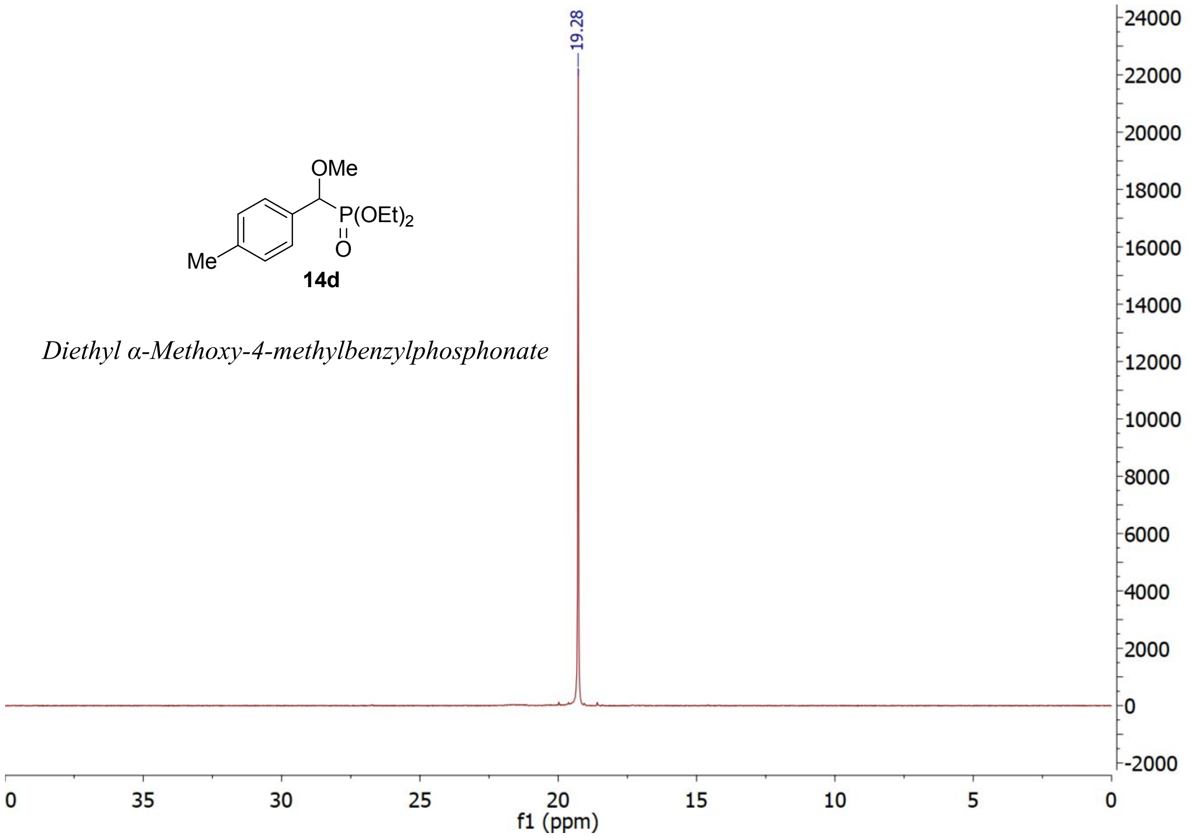

^{31}P {^{1}H} NMR (202 MHz, $CDCl_3$) spectra for 14e

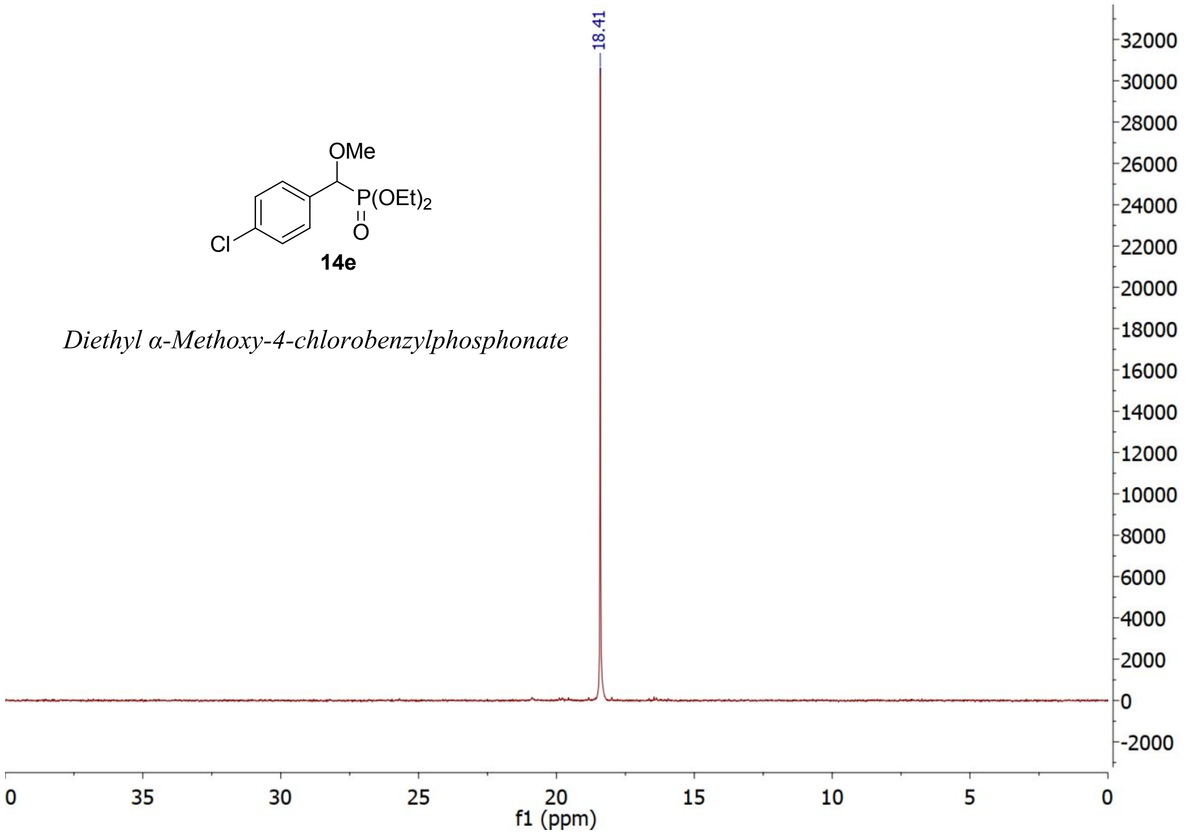

^{31}P {^{1}H} NMR (202 MHz, $CDCl_3$) spectra for 15a

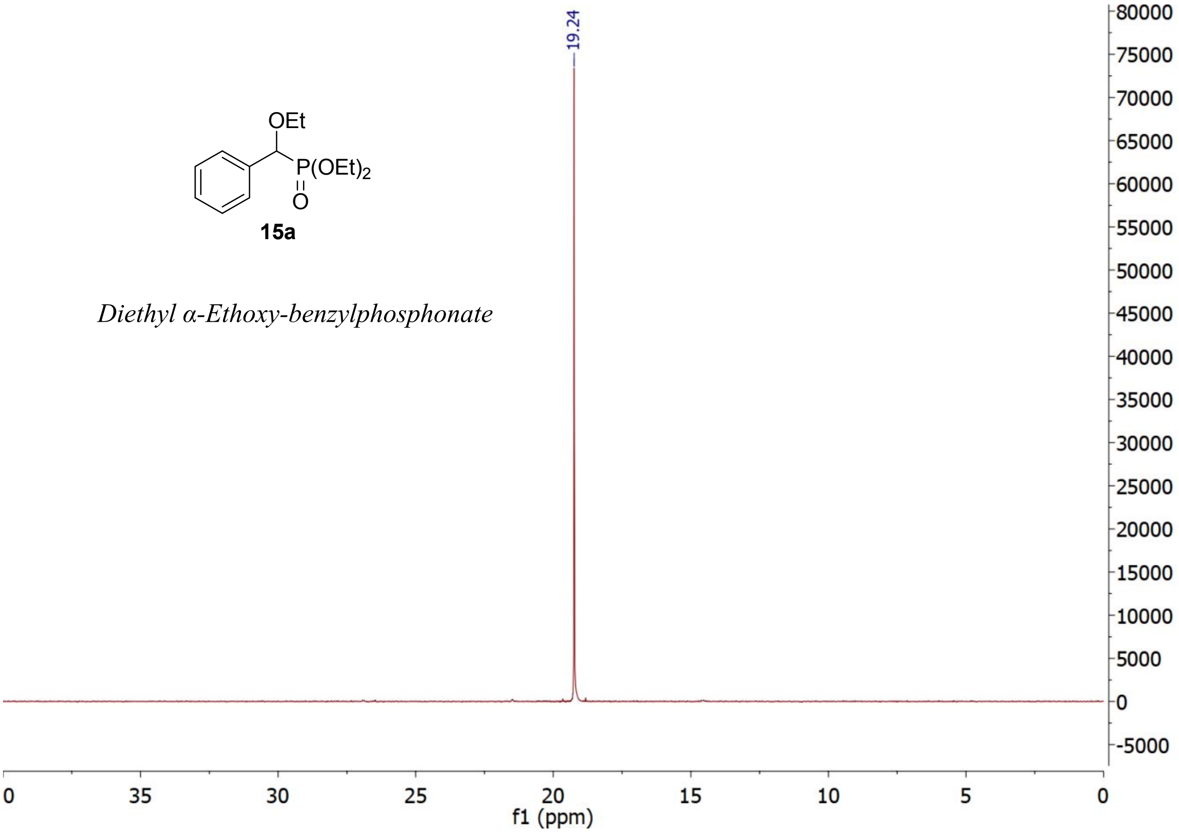

^{13}C {^{1}H} NMR (75 MHz, $CDCl_3$) spectra for 15a

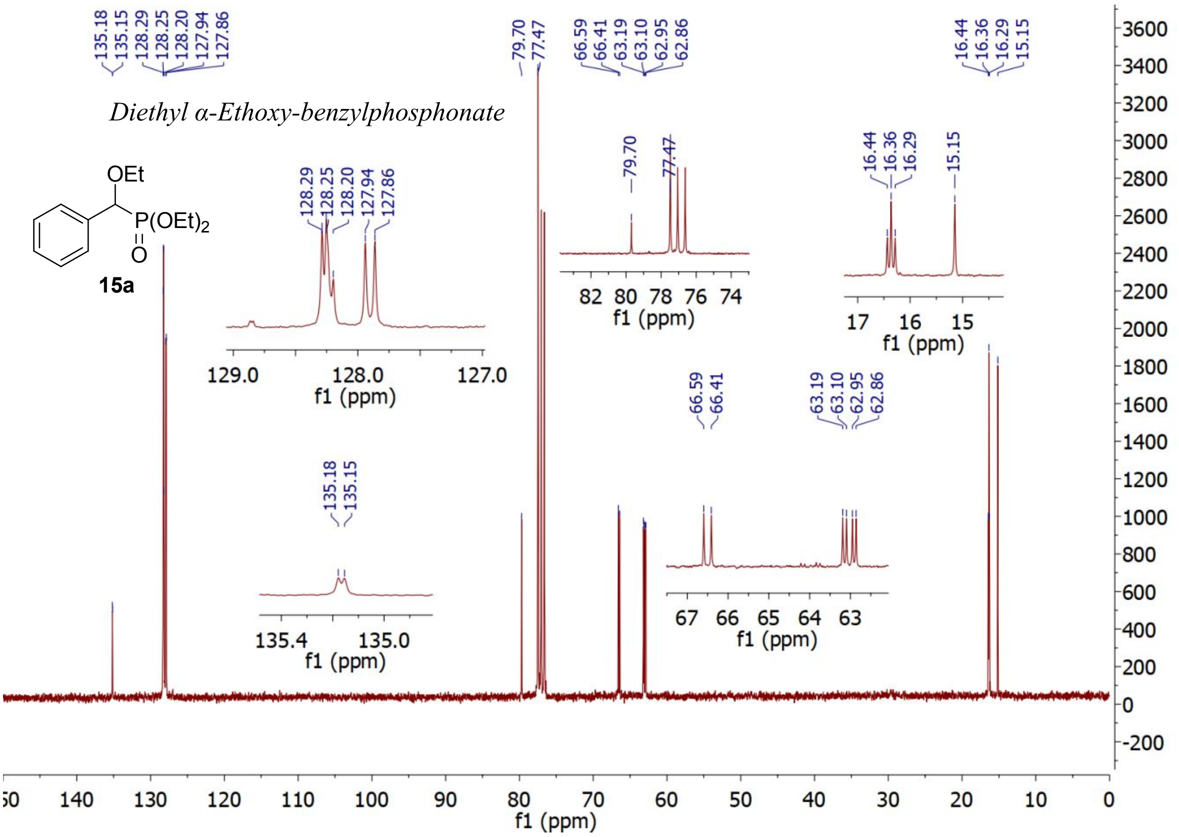

^{31}P {^{1}H} NMR (122 MHz, $CDCl_3$) spectra for 15d

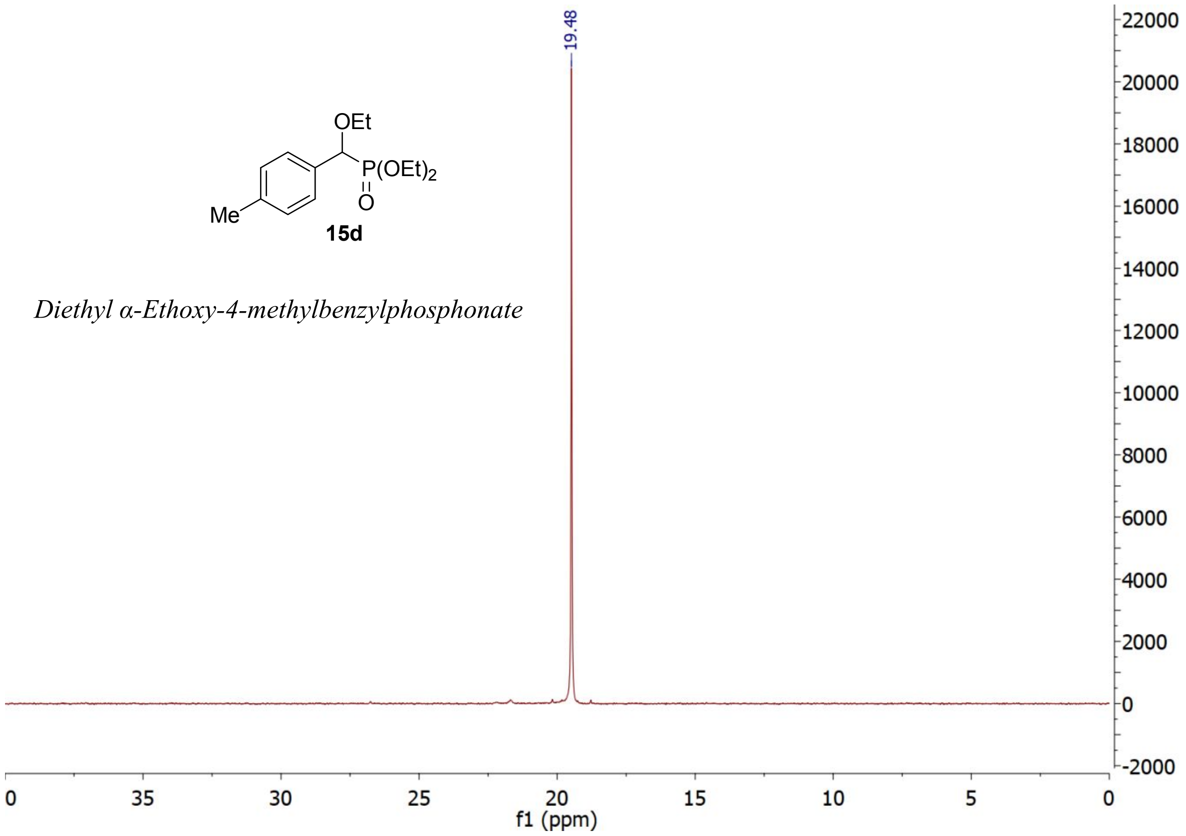

^{31}P {^{1}H} NMR (202 MHz, $CDCl_3$) spectra for 15e

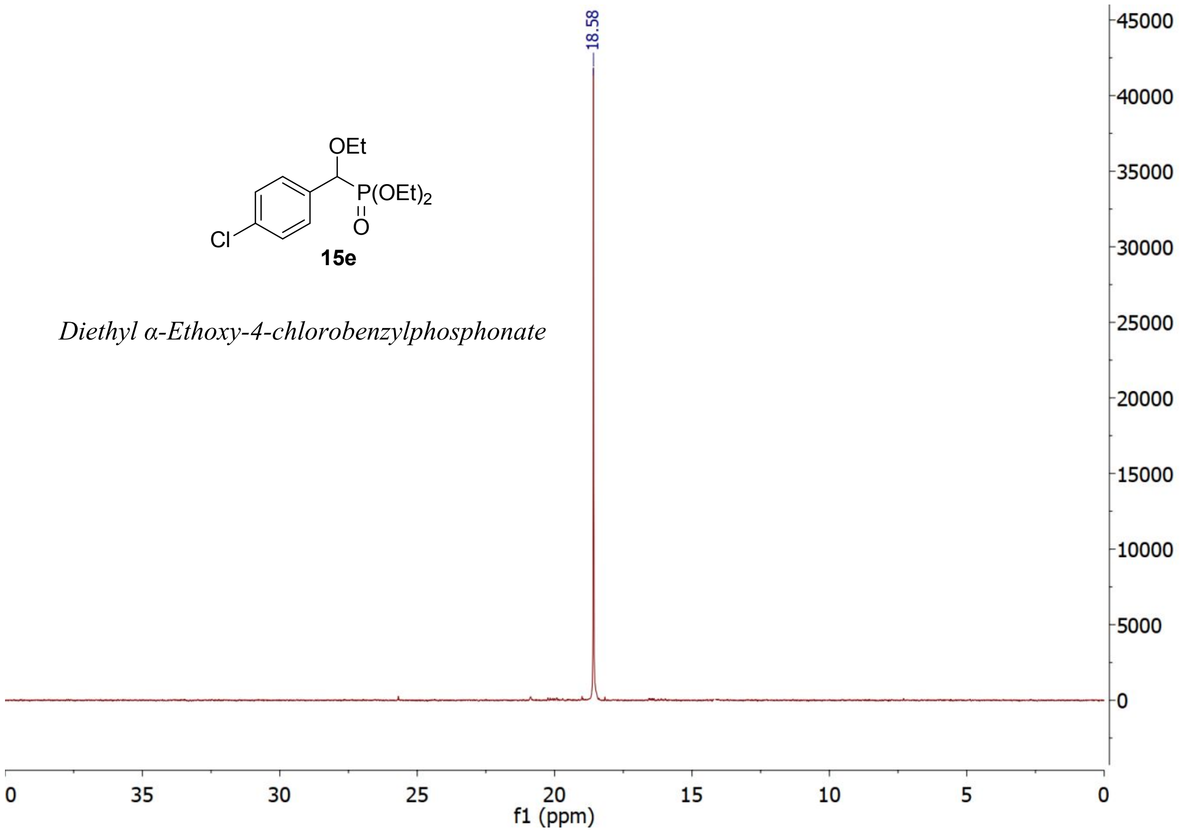

^{13}C {^{1}H} NMR (126 MHz, $CDCl_3$) spectra for 15e

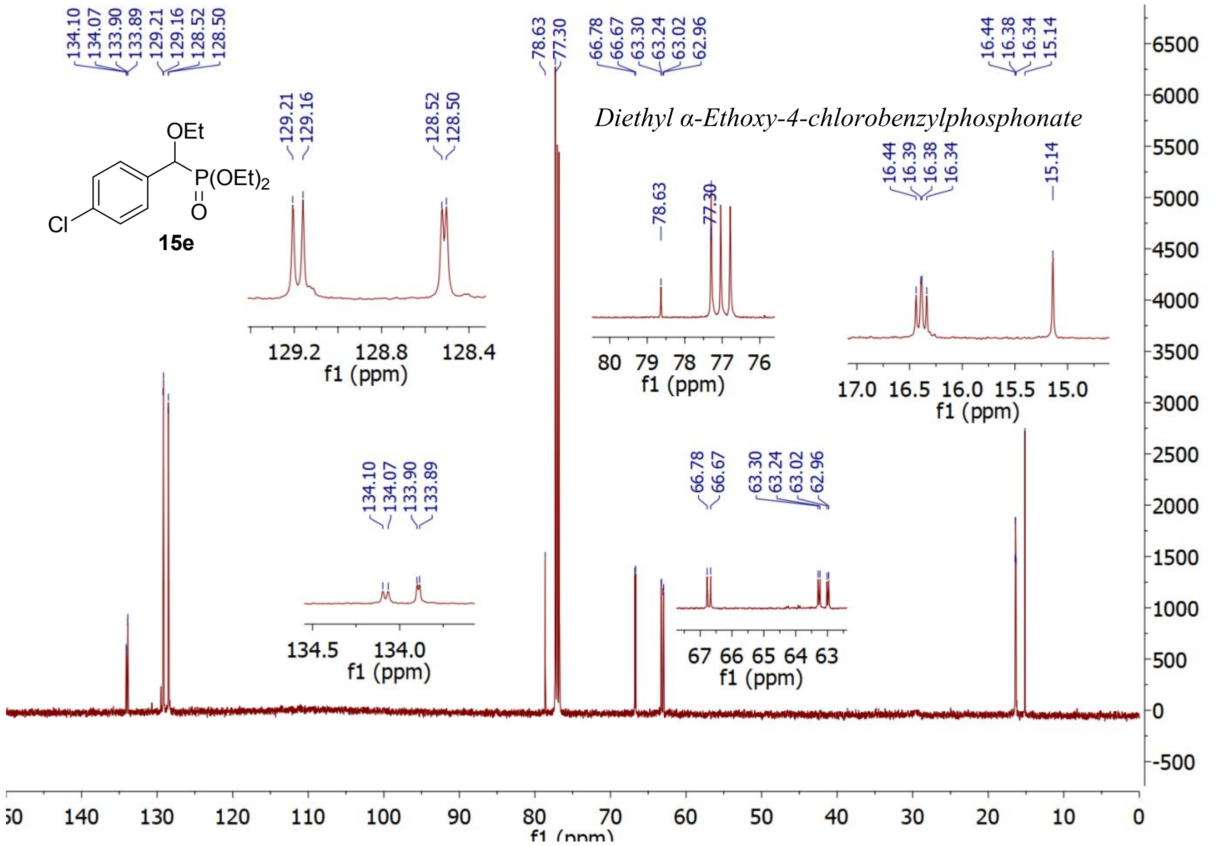

^{31}P {^{1}H} NMR (202 MHz, $CDCl_3$) spectra for 16a

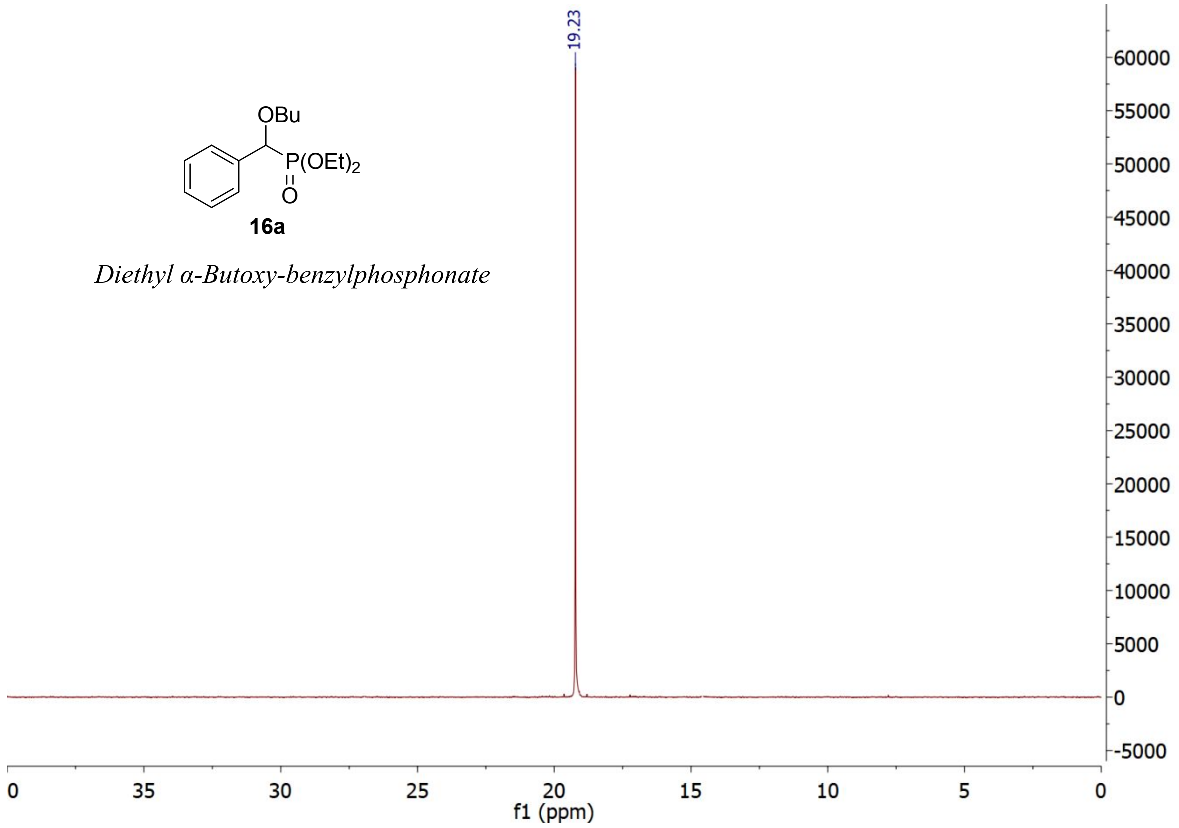

^{13}C {^{1}H} NMR (75 MHz, $CDCl_3$) spectra for 16a

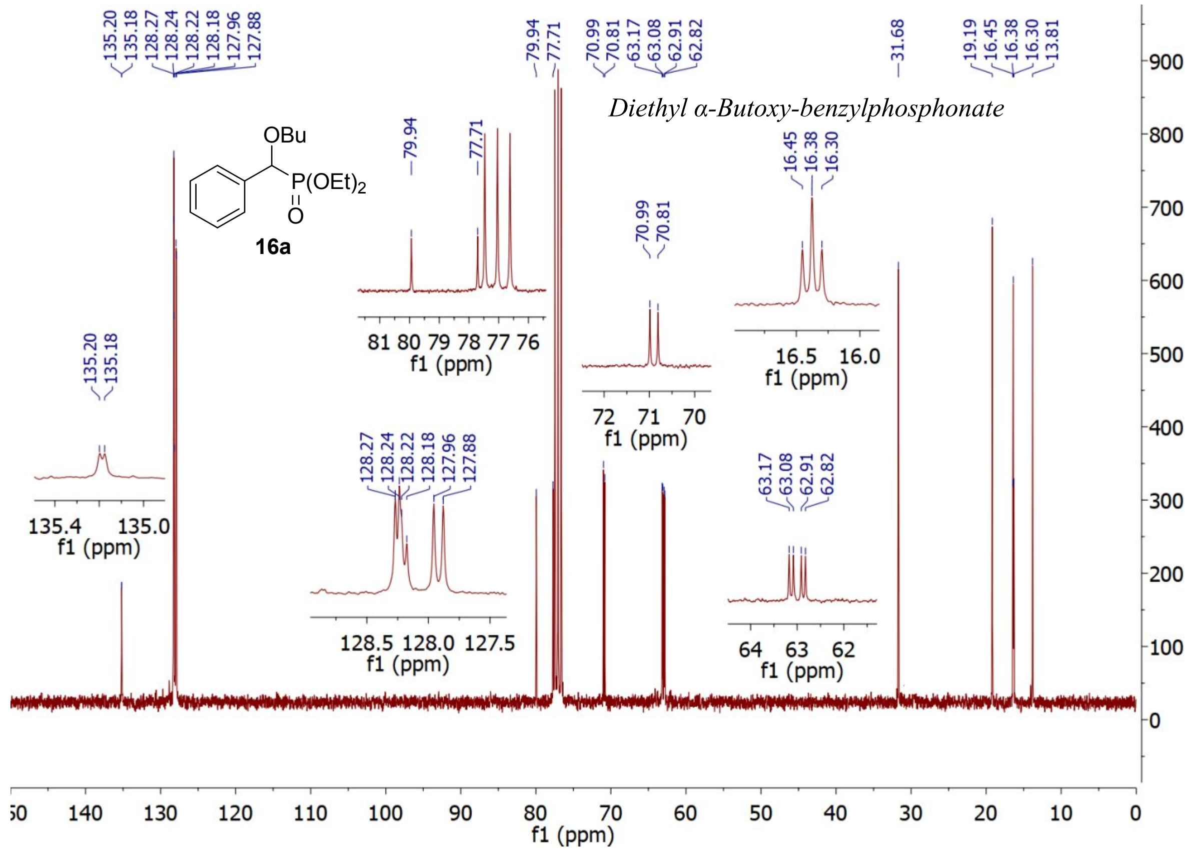

^{1}H NMR (500 MHz, $CDCl_3$) spectra for 16a

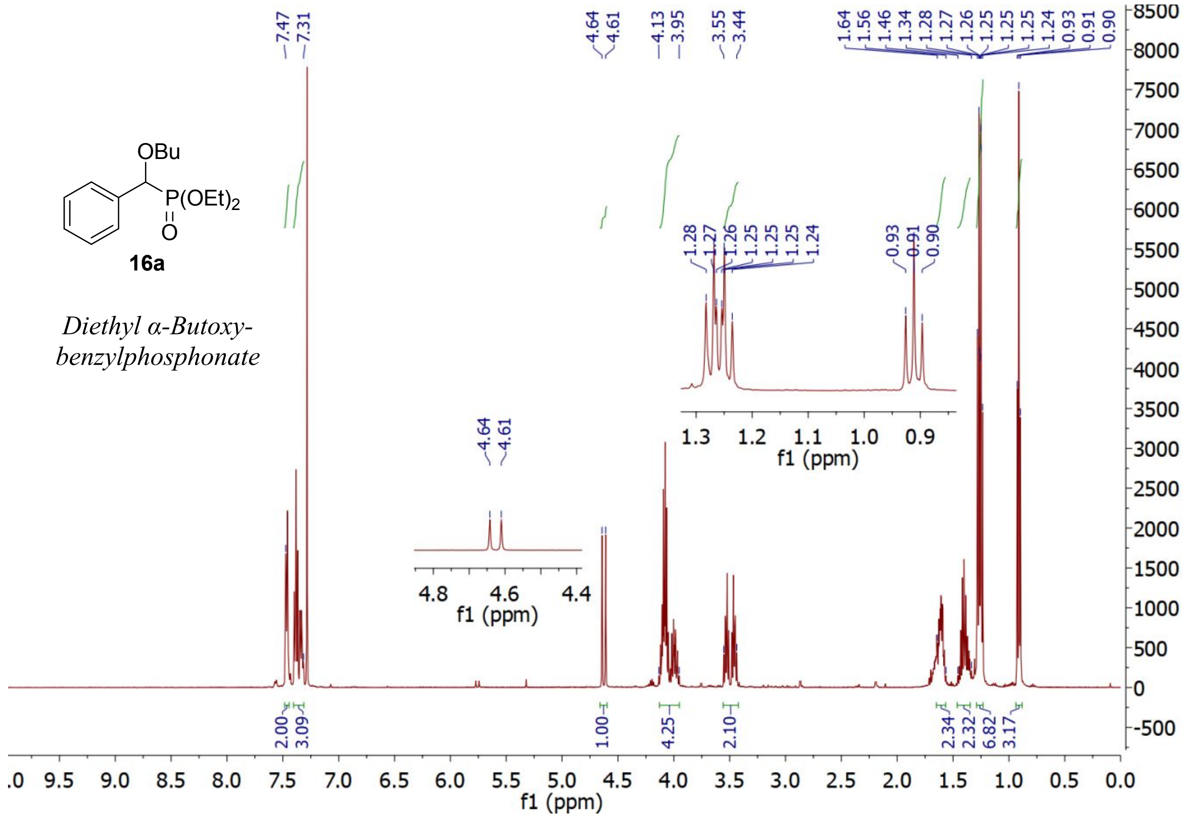

^{31}P {^{1}H} NMR (122 MHz, CDCl$_3$) spectra for 16d

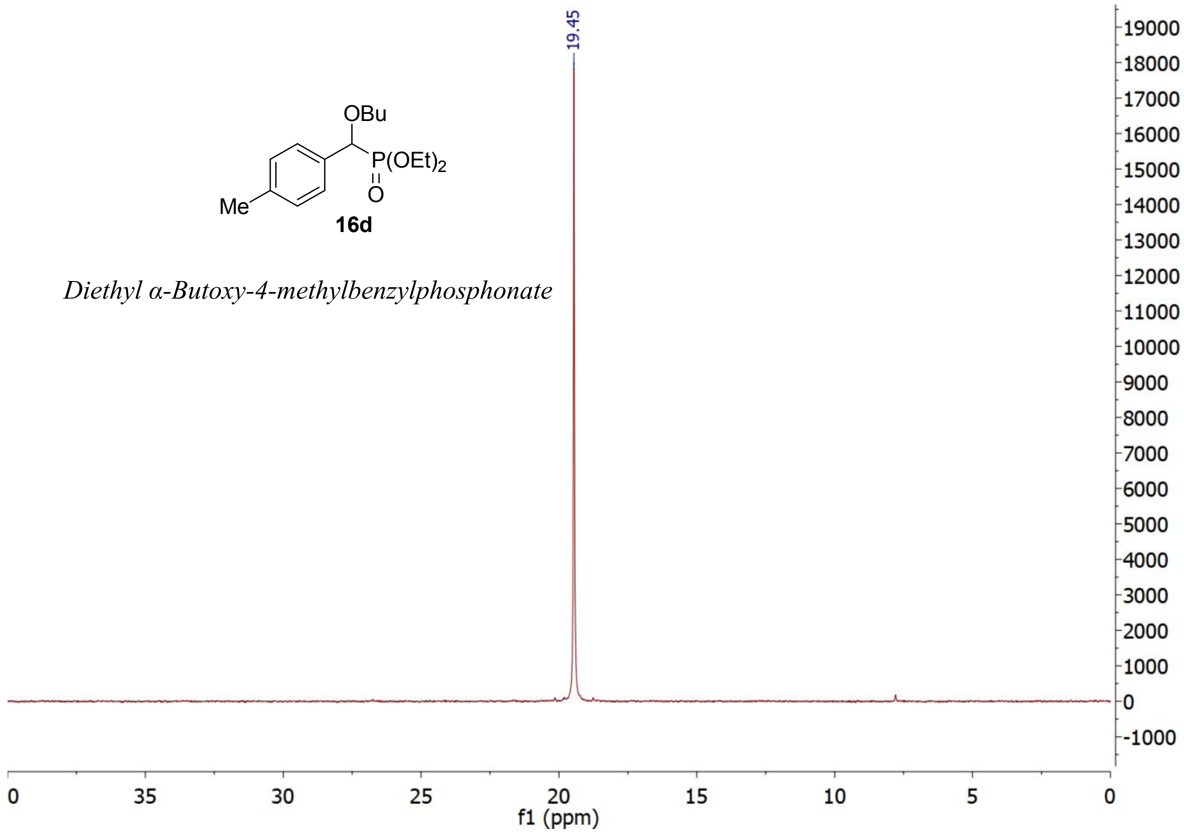

^{13}C {^{1}H} NMR (75 MHz, CDCl$_3$) spectra for 16d

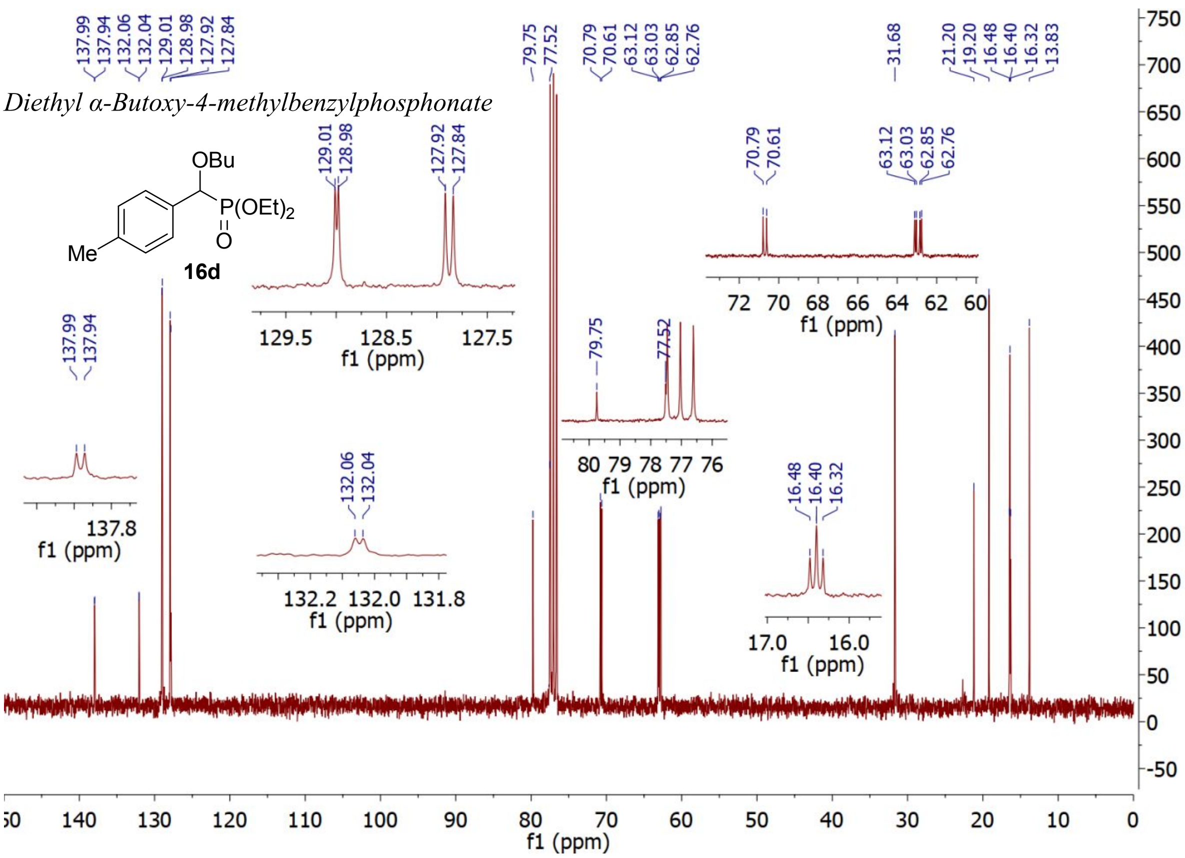

^{1}H NMR (500 MHz, $CDCl_3$) spectra for 16d

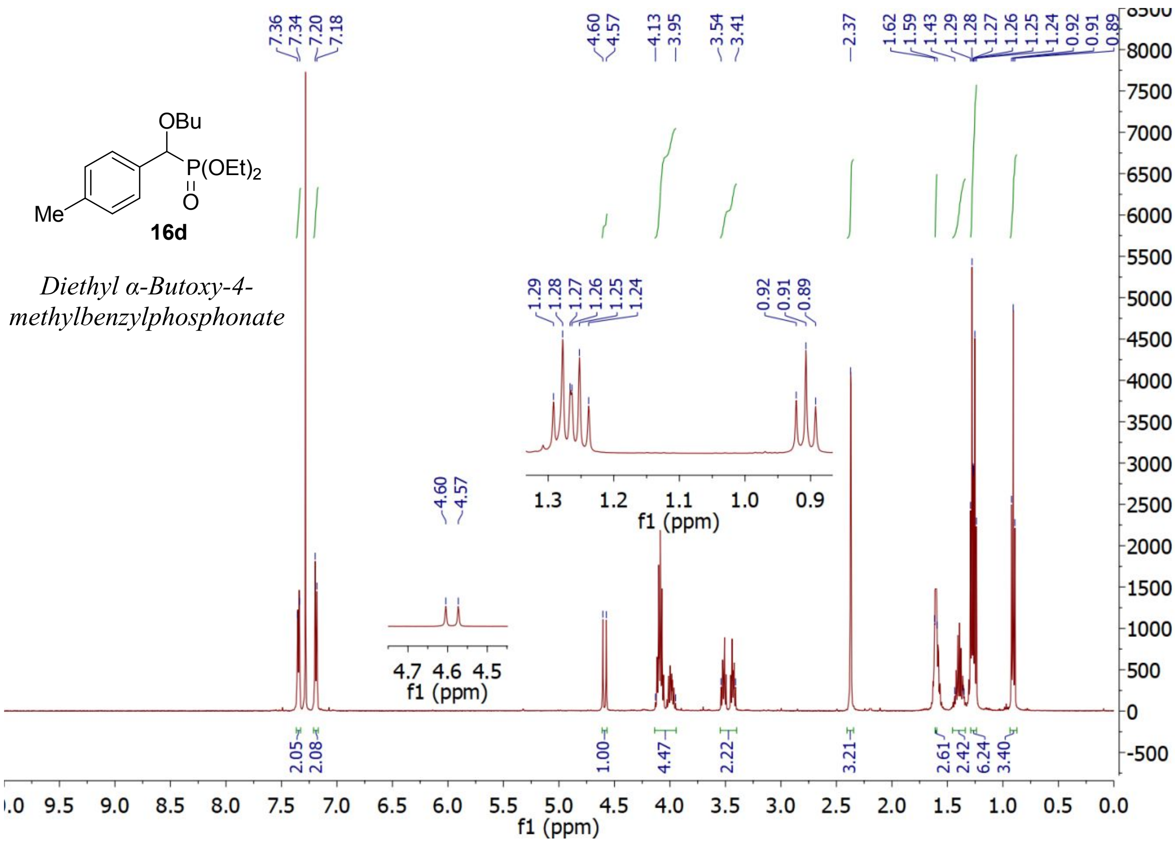

^{31}P {^{1}H} NMR (202 MHz, $CDCl_3$) spectra for 16e

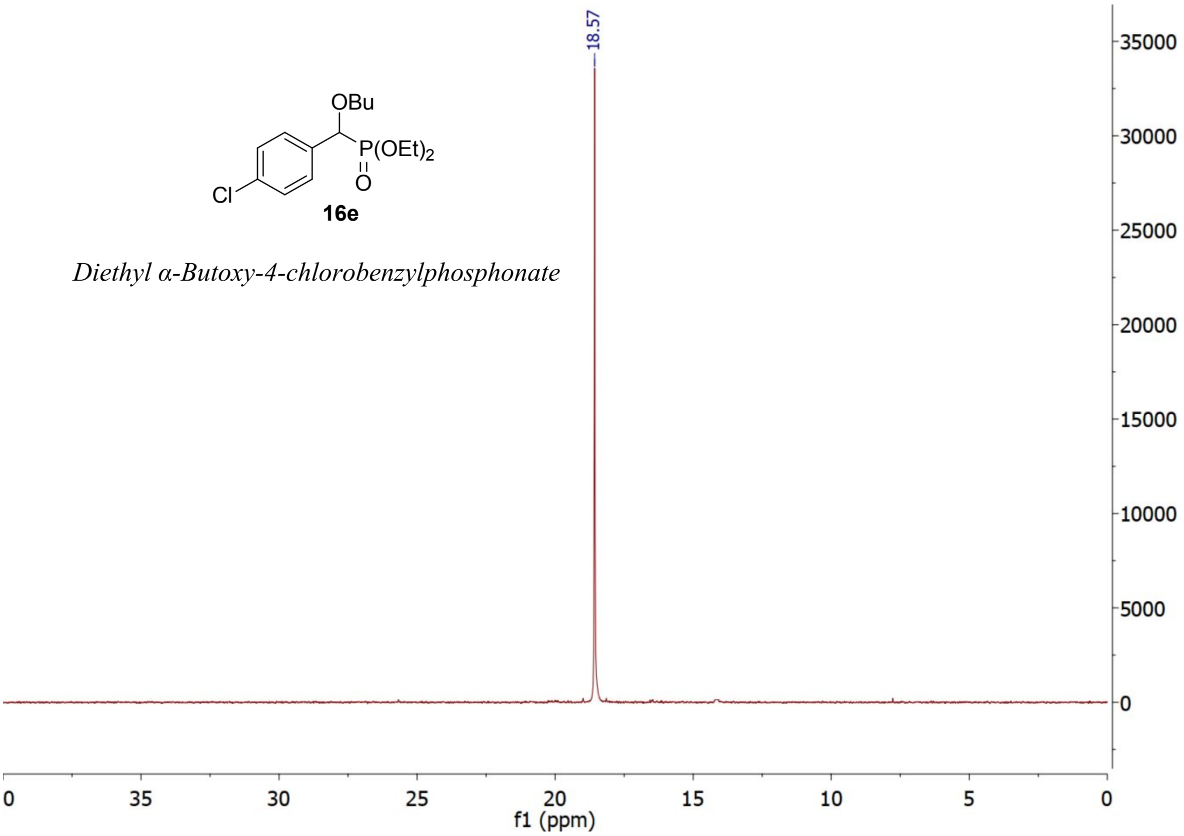

^{13}C {^{1}H} NMR (126 MHz, $CDCl_3$) spectra for 16e

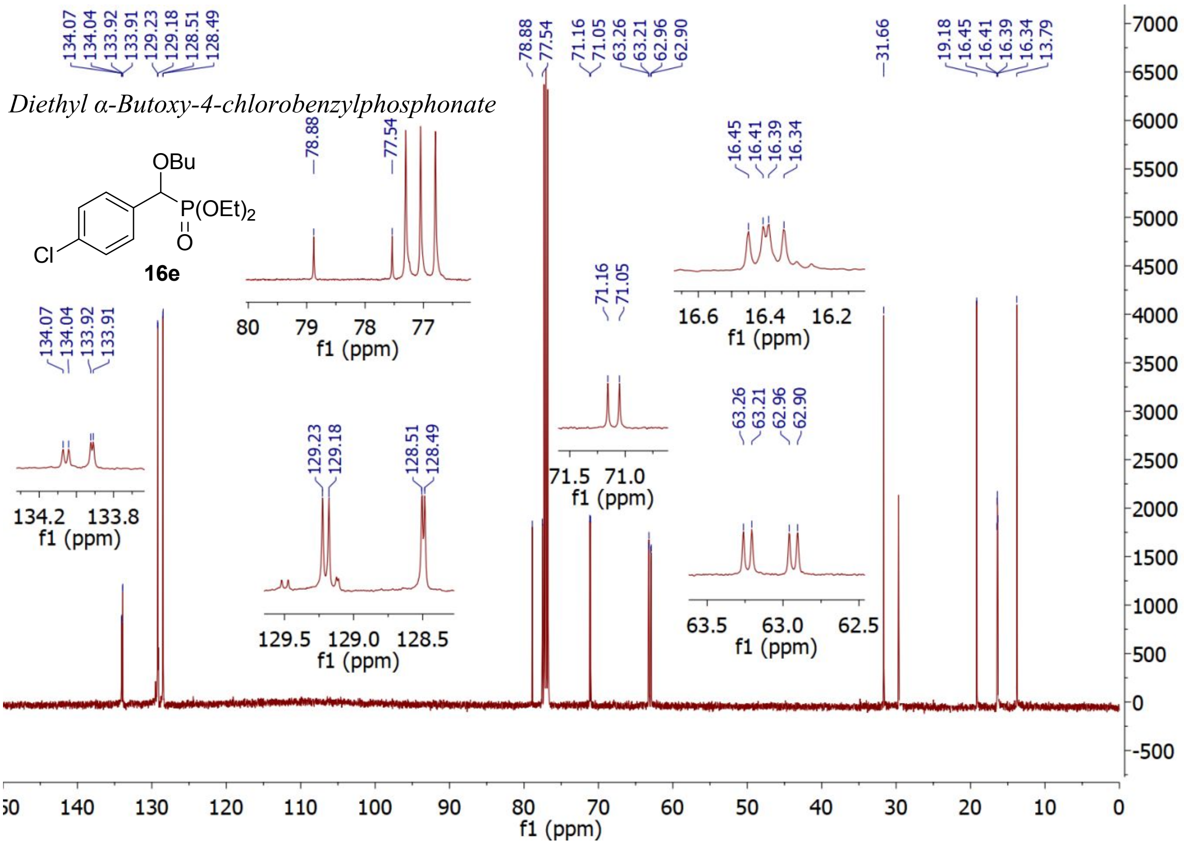

^{1}H NMR (500 MHz, $CDCl_3$) spectra for 16e

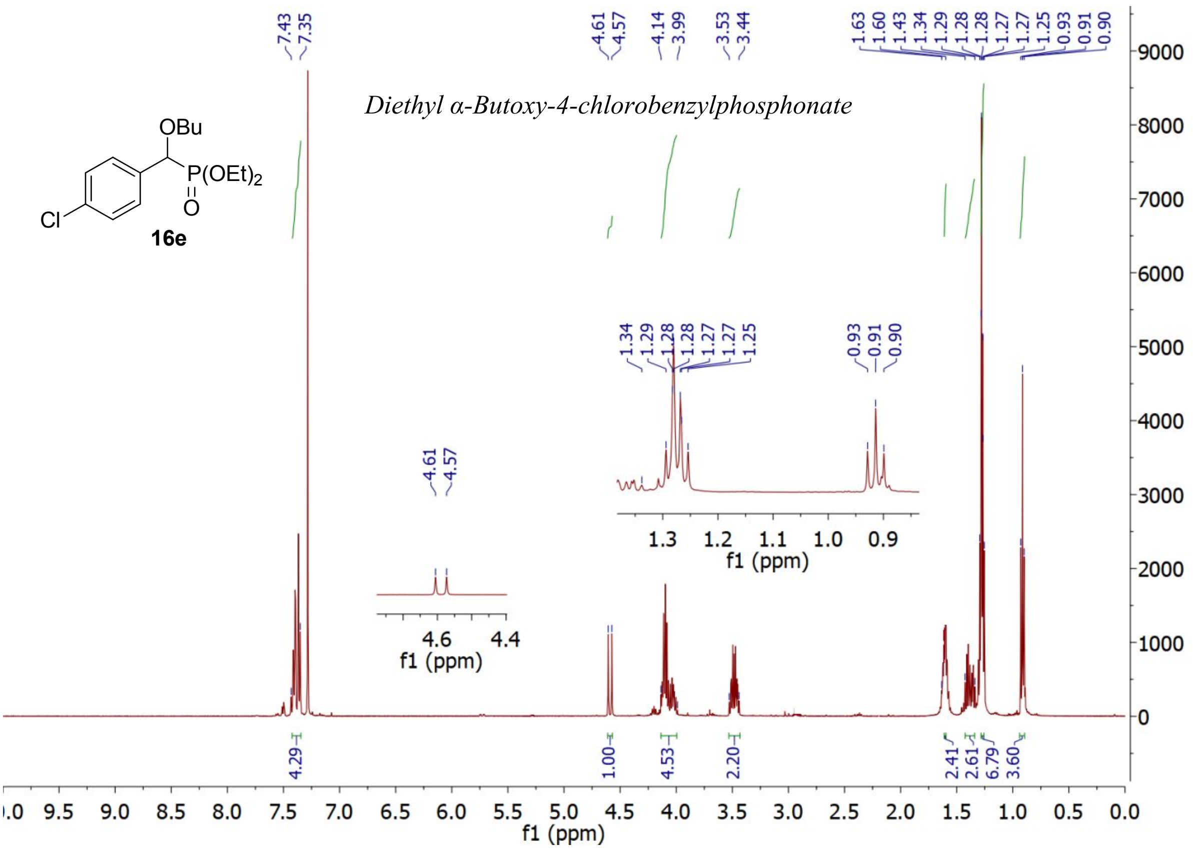

^{31}P {^{1}H} NMR (202 MHz, $CDCl_3$) spectra for 18a

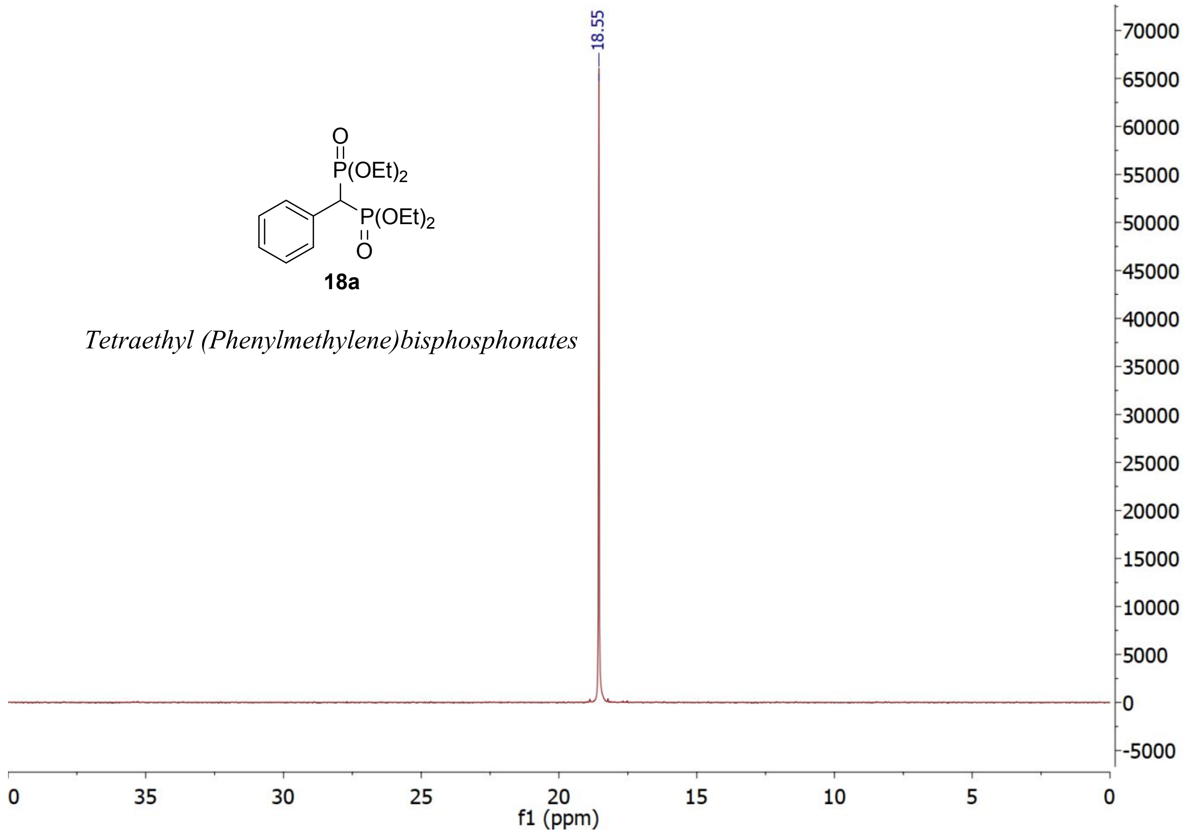

^{31}P {^{1}H} NMR (202 MHz, $CDCl_3$) spectra for 18d

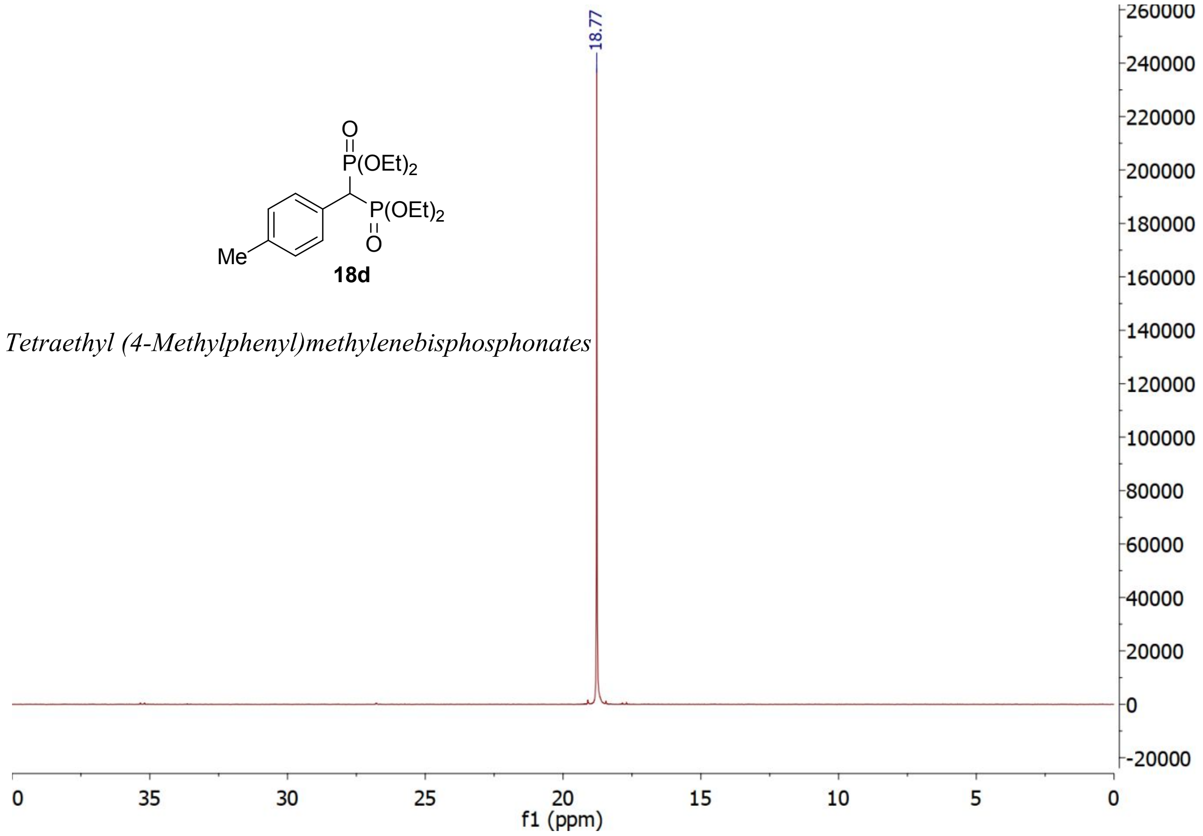

^{13}C {^{1}H} NMR (75 MHz, $CDCl_3$) spectra for 18d

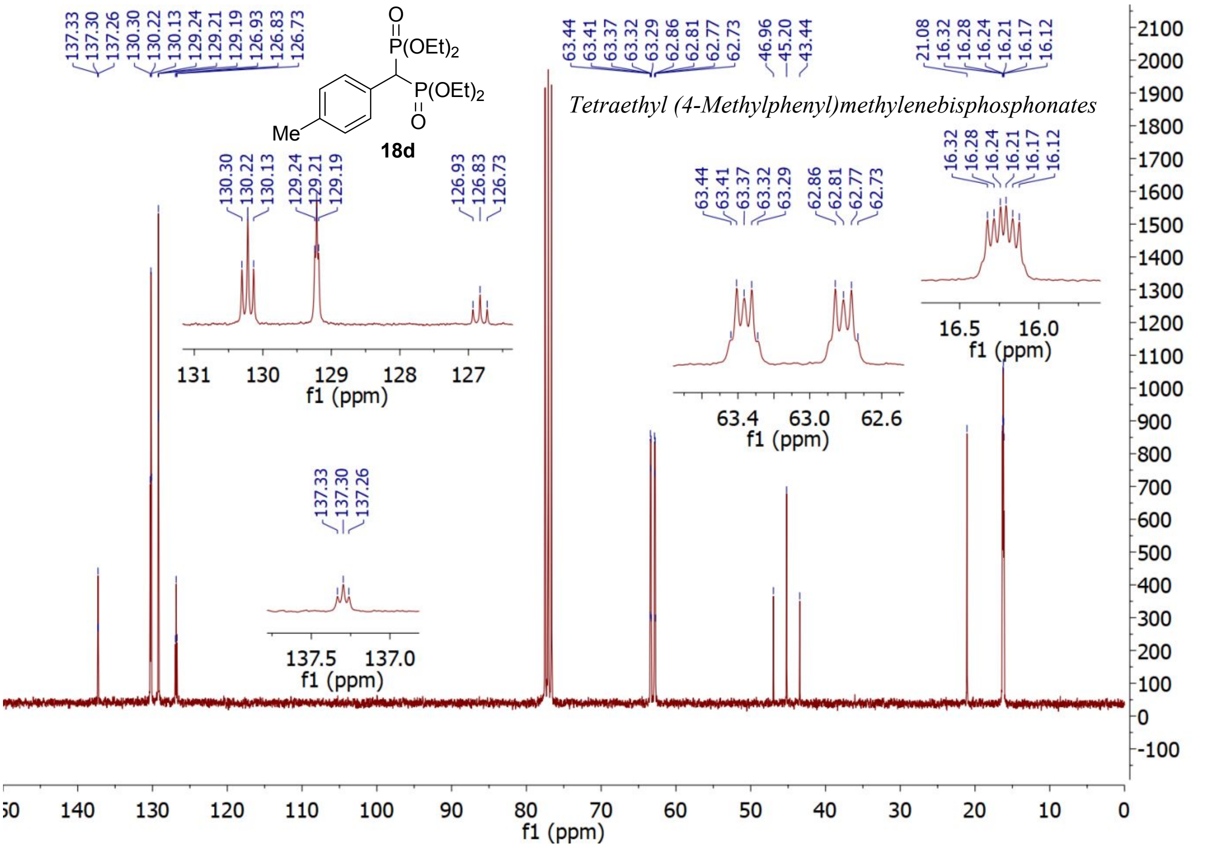

^{1}H NMR (500 MHz, $CDCl_3$) spectra for 18d

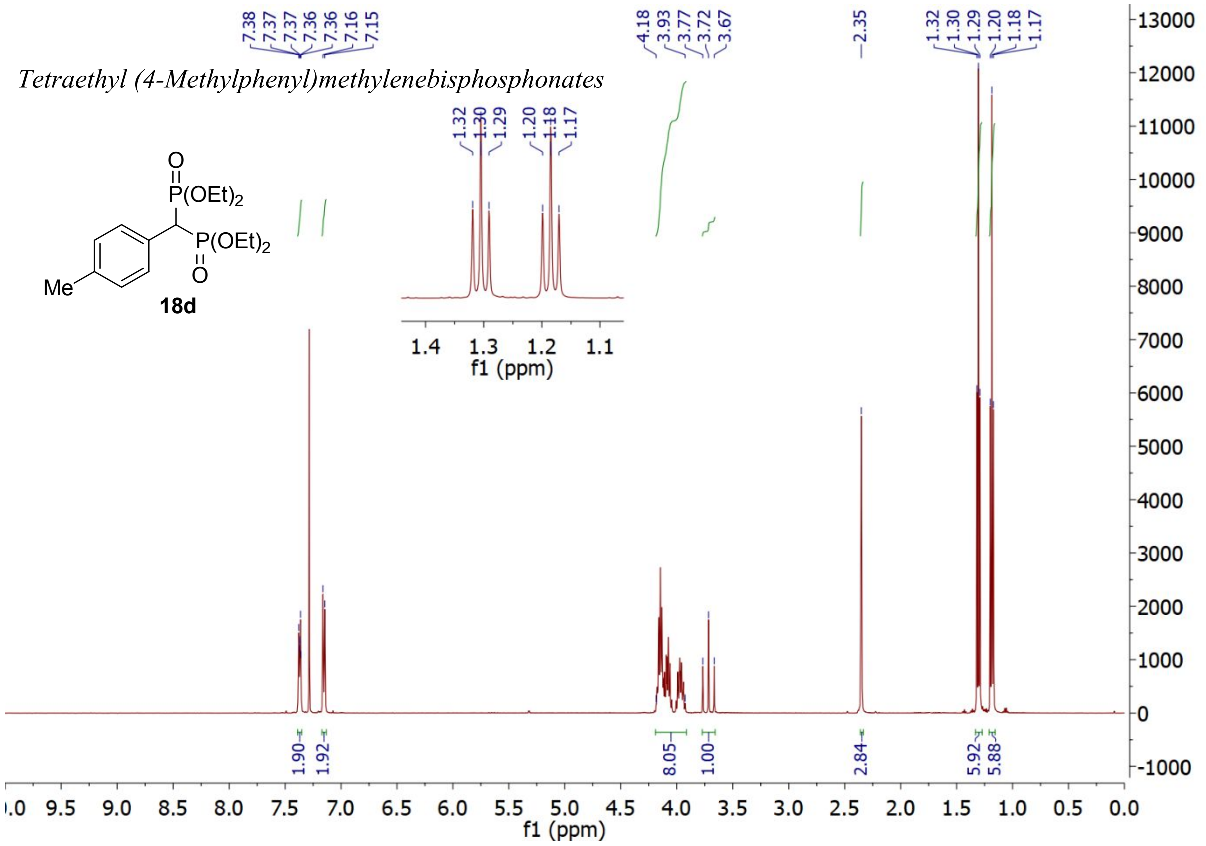

^{31}P {^{1}H} NMR (202 MHz, $CDCl_3$) spectra for 18e

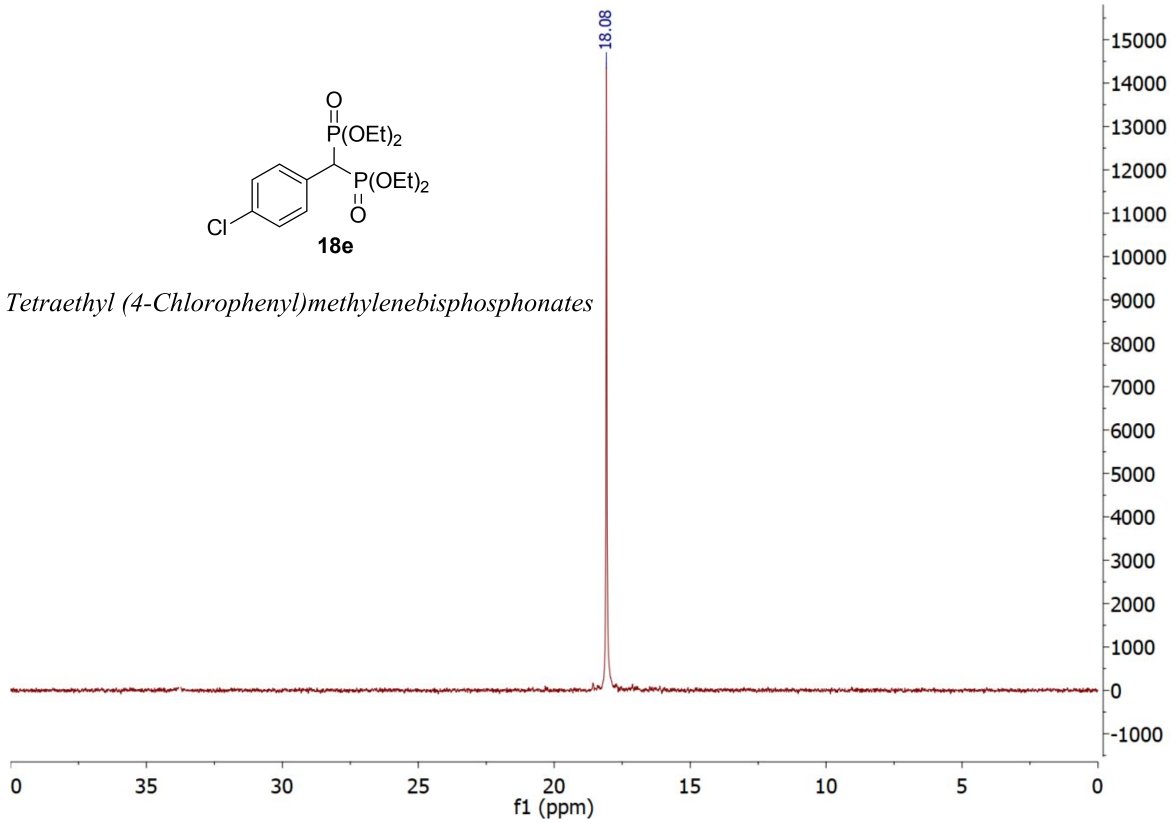

^{13}C {^{1}H} NMR (126 MHz, $CDCl_3$) spectra for 18e

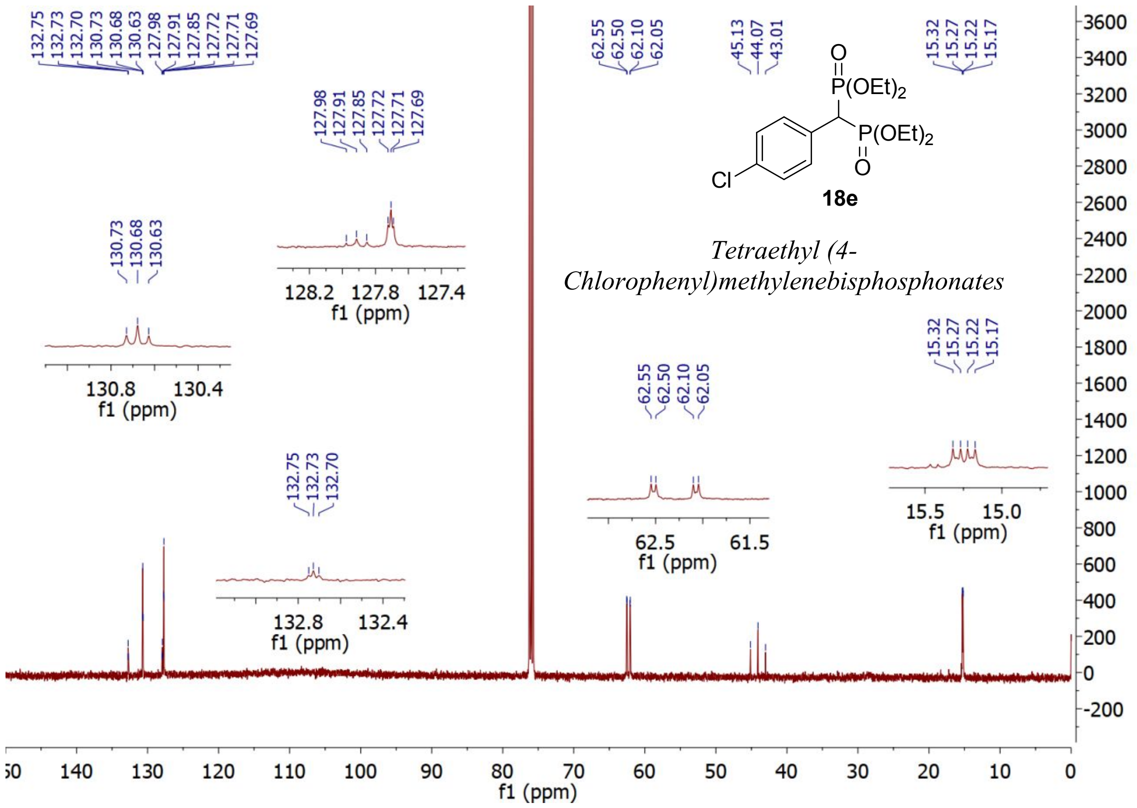

^{1}H NMR (500 MHz, $CDCl_3$) spectra for 18e

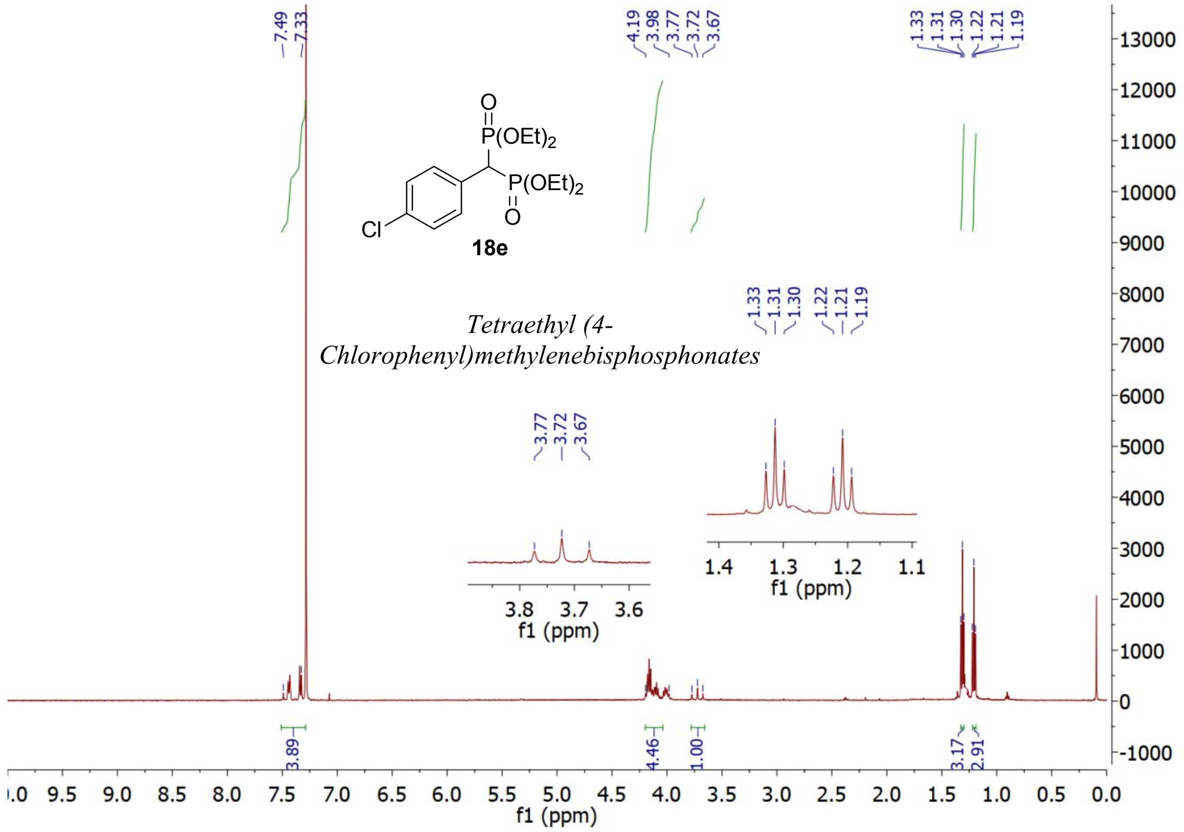

2. Geometrical data for compound 5e obtained from the X-ray measurement

SI Table 1. Selected bond lengths (Å) of compound **5e**.

P1 – O4	1.469(1)	C9 – C10	1.492(2)
P1 – O5	1.565(1)	C8 – C7	1.387(2)
P1 – O6	1.569(1)	C11 – C12	1.501(2)
P1 – C2	1.817(2)	C6 – C7	1.378(2)
S1 – O3	1.424(1)	C6 – C5	1.386(2)
S1 – O2	1.428(1)	C4 – C5	1.384(2)
S1 – O1	1.579(1)	C3 – C8	1.392(2)
S1 – C1	1.750(2)	C3 – C4	1.397(2)
Cl1 – C6	1.744(2)	O5 – C9	1.452(2)
C2 – O1	1.469(2)	O6 – C11	1.463(2)
C2 – C3	1.503(2)		

SI Table 2. Selected bond angles (°) of compound **5e**.

O4 – P1 – O5	116.4(1)	C7 – C8 – C3	120.5(1)
O4 – P1 – O6	115.3(1)	O6 – C11 – C12	110.4(1)
O5 – P1 – O6	104.6(1)	C6 – C7 – C8	119.2(2)
O4 – P1 – C2	113.3(1)	C2 – O1 – S1	118.6(1)
O5 – P1 – C2	101.0(1)	C8 – C3 – C4	119.3(1)
O6 – P1 – C2	104.6(1)	C8 – C3 – C2	119.3(1)
O3 – S1 – O2	119.9(1)	C4 – C3 – C2	121.3(1)
O3 – S1 – O1	109.4(1)	C9 – O5 – P1	120.4(1)
O2 – S1 – O1	103.5(1)	C11 – O6 – P1	121.3(1)
O3 – S1 – C1	109.4(1)	C7 – C6 – C5	121.5(2)
O2 – S1 – C1	109.5(1)	C7 – C6 – Cl1	119.6(1)
O1 – S1 – C1	103.9(1)	C5 – C6 – Cl1	119.0(1)
O1 – C2 – C3	111.9(1)	C5 – C4 – C3	120.4(1)
O1 – C2 – P1	103.2(1)	C4 – C5 – C6	119.2(1)
C3 – C2 – P1	111.4(1)	O5 – C9 – C10	108.4(1)

SI Table 3. Selected torsion angles (°) of compound **5e**.

O4 – P1 – C2 – O1	61.1(1)	C2 – P1 – O5 – C9	163.6(1)
O5 – P1 – C2 – O1	-64.1(1)	O4 – P1 – O6 – C11	23.3(1)
O6 – P1 – C2 – O1	-172.5(1)	O5 – P1 – O6 – C11	152.5(1)
O4 – P1 – C2 – C3	-59.1(1)	C2 – P1 – O6 – C11	-101.8(1)
O5 – P1 – C2 – C3	175.7(1)	C8 – C3 – C4 – C5	-0.8(2)
O6 – P1 – C2 – C3	67.3(1)	C2 – C3 – C4 – C5	-176.6(1)
C3 – C2 – O1 – S1	-94.8(1)	C3 – C4 – C5 – C6	0.9(2)
P1 – C2 – O1 – S1	145.3(1)	C7 – C6 – C5 – C4	-0.3(2)
O3 – S1 – O1 – C2	46.4(1)	Cl1 – C6 – C5 – C4	179.4(1)
O2 – S1 – O1 – C2	175.2(1)	P1 – O5 – C9 – C10	-176.1(1)
C1 – S1 – O1 – C2	-70.4(1)	C4 – C3 – C8 – C7	0.1(2)
O1 – C2 – C3 – C8	151.5(1)	C2 – C3 – C8 – C7	176.0(1)
P1 – C2 – C3 – C8	-93.5(1)	P1 – O6 – C11 – C12	-90.1(2)
O1 – C2 – C3 – C4	-32.7(2)	C5 – C6 – C7 – C8	-0.4(2)
P1 – C2 – C3 – C4	82.2(2)	Cl1 – C6 – C7 – C8	179.9(1)
O4 – P1 – O5 – C9	40.5(1)	C3 – C8 – C7 – C6	0.5(2)
O6 – P1 – O5 – C9	-88.0(1)		

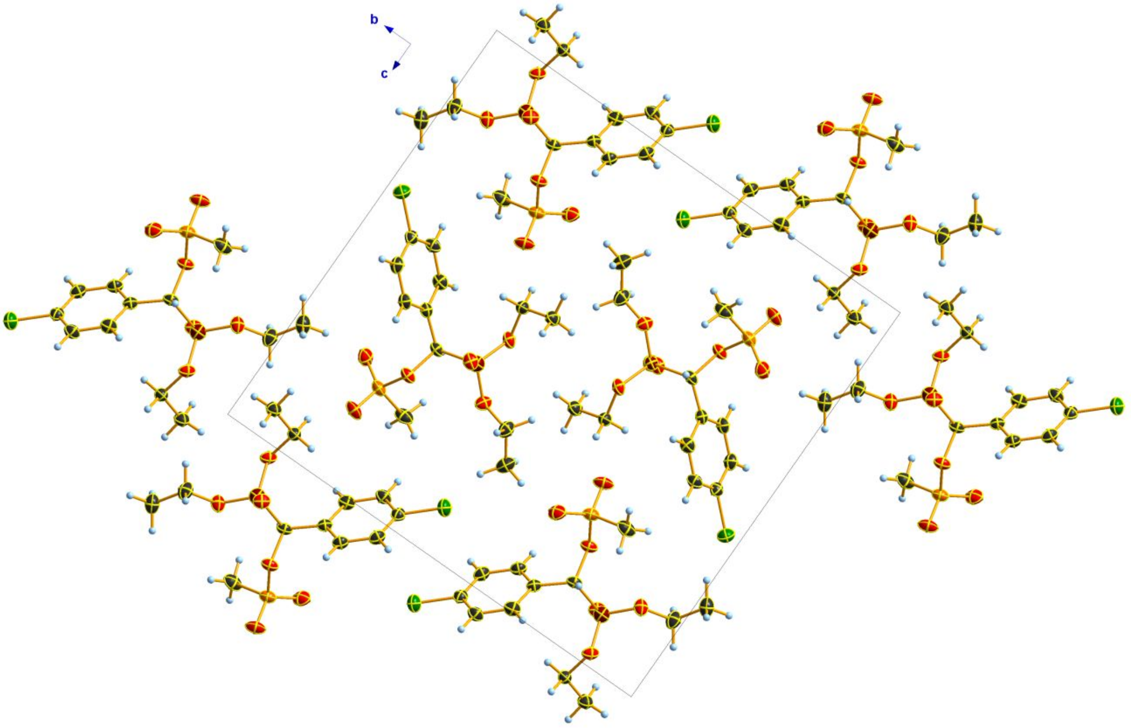

SI Figure 1. Crystal structure of compound **5e**, view of the unit cell along the *a*-axis. DIAMOND representation; thermal ellipsoids are drawn at 50% probability level.

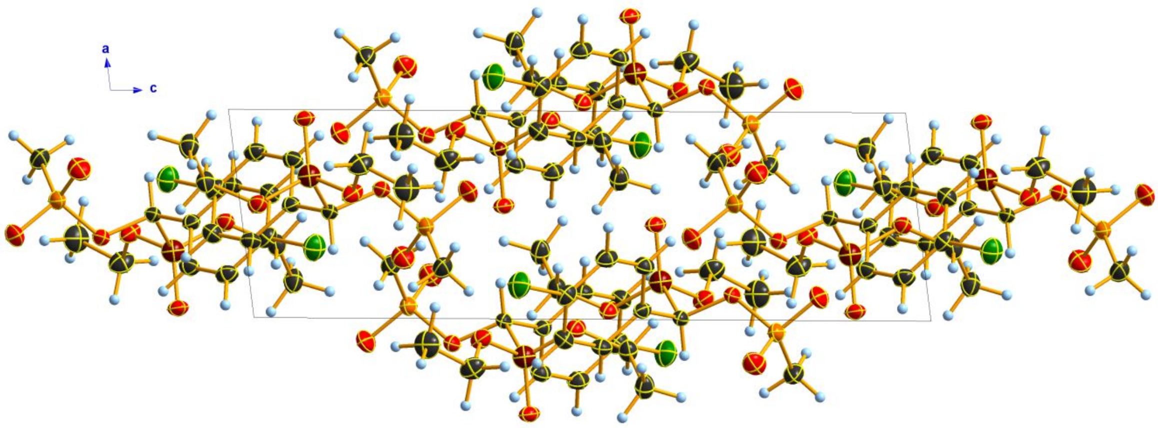

SI Figure 2. Crystal structure of compound **5e**, view of the unit cell along the *b*-axis. DIAMOND representation; thermal ellipsoids are drawn at 50% probability level.

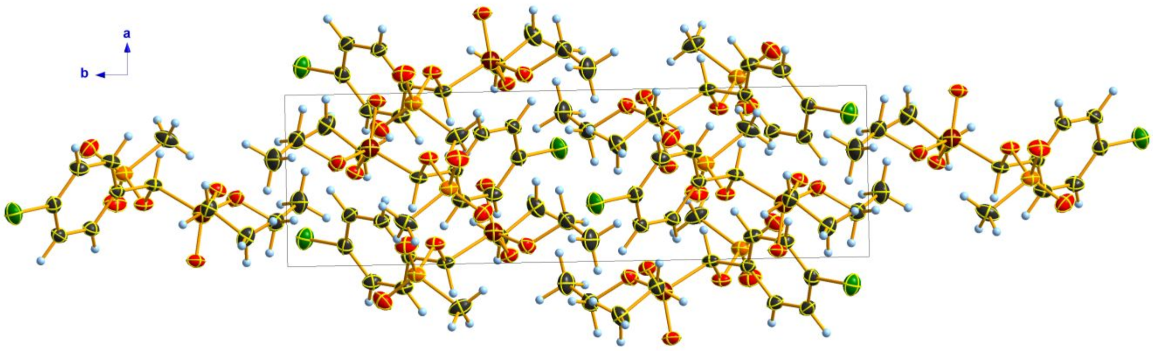

SI Figure 3. Crystal structure of compound **5e**, view of the unit cell along the *c*-axis. DIAMOND representation; thermal ellipsoids are drawn at 50% probability level.

3. Computed energy values and coordinates

SI Table 4. Computed energy and entropy values in kJ mol^{-1} and cal molK^{-1} as copied from the corresponding Gaussian output file

	E	ZPE	U	H	G	S	Svib
H_carbocation	-995.362	-995.103	-995.088	-995.087	-995.147	125.737	50.621
**10**	-1109.86	-1109.57	-1109.55	-1109.55	-1109.61	138.88	63.057
3OMe_carbocation	-1109.84	-1109.55	-1109.53	-1109.53	-1109.6	140.598	64.272
**7**	-1109.87	-1109.58	-1109.56	-1109.56	-1109.62	140.187	63.751
TEAH^{+}	-292.663	-292.439	-292.43	-292.429	-292.473	92.922	25.002
OMs^{-} + TEAH^{+}	-956.501	-956.227	-956.211	-956.21	-956.271	127.792	54.615
**5a**	-1659.22	-1658.91	-1658.89	-1658.89	-1658.96	162.273	84.802
**5h**	-1773.7	-1773.35	-1773.33	-1773.33	-1773.41	169.927	91.867
**5c**	-1773.71	-1773.36	-1773.33	-1773.33	-1773.42	175.866	97.435
**5g**	-1773.71	-1773.36	-1773.34	-1773.34	-1773.42	173.417	94.953

5h

Center Number	Atomic Number	Forces (Hartrees/Bohr) X	Y	Z
1	6	-0.000000669	0.000001807	-0.000002315
2	6	-0.000001348	0.000002508	-0.000003269
3	6	-0.000000898	0.000000949	-0.000002021
4	6	0.000000177	0.000000895	-0.000002575
5	6	0.000000033	0.000001743	-0.000001759
6	6	0.000001208	0.000000231	-0.000003326
7	1	-0.000001412	0.000003371	-0.000003544
8	1	-0.000001056	0.000002773	-0.000002371
9	1	0.000000560	-0.000000678	-0.000003319
10	6	0.000000395	0.000003899	-0.000002729
11	8	-0.000000358	-0.000003688	0.000002050
12	1	-0.000000201	0.000001205	-0.000000340
13	15	0.000001862	0.000001411	0.000000832
14	8	0.000000083	-0.000001572	-0.000000704
15	8	-0.000000242	0.000001596	0.000004349
16	8	-0.000003284	-0.000008436	0.000001685
17	6	0.000001805	0.000004341	0.000003108
18	1	-0.000000271	-0.000001194	0.000002920
19	6	-0.000000503	-0.000001540	-0.000001459
20	1	-0.000001561	-0.000000145	0.000004466
21	1	-0.000000133	0.000000582	0.000001827
22	1	0.000001283	-0.000002721	0.000002850
23	16	0.000001251	0.000003322	-0.000001752
24	8	0.000001797	-0.000002811	-0.000000890
25	8	0.000001694	-0.000001784	0.000001851
26	6	0.000000377	-0.000000693	-0.000002798
27	1	-0.000000989	0.000000173	-0.000001760
28	1	0.000000284	-0.000000156	-0.000003912

29	1	0.000000888	-0.000000378	-0.000002542
30	1	-0.000000566	0.000002079	-0.000003999
31	8	0.000000214	-0.000003306	-0.000001270
32	6	0.000000529	0.000000566	-0.000001401
33	1	0.000001393	-0.000001763	-0.000000568
34	1	0.000001999	-0.000002463	-0.000000700
35	1	0.000002036	-0.000002337	-0.000002212
36	6	-0.000001460	0.000000720	0.000003176
37	6	-0.000000434	0.000000395	0.000000675
38	1	-0.000000485	-0.000001143	0.000004230
39	1	-0.000000437	0.000000041	0.000003821
40	1	0.000000110	-0.000000992	0.000005296
41	1	-0.000001044	0.000000515	0.000004191
42	1	-0.000001790	0.000001692	0.000003742
43	1	-0.000000839	0.000000987	0.000002465

10

Center Number	Atomic Number	Forces (Hartrees/Bohr) X	Y	Z
1	6	0.000030740	0.000019685	-0.000039122
2	6	0.000082043	0.000001832	-0.000023382
3	6	-0.000137794	0.000020493	-0.000018032
4	6	-0.000089401	0.000010440	0.000023726
5	6	-0.000037893	0.000015622	0.000051407
6	6	-0.000058359	-0.000020502	0.000004001
7	1	-0.000014649	0.000018236	-0.000014291
8	1	-0.000022161	0.000012824	-0.000034648
9	1	-0.000000359	0.000017003	0.000014477
10	6	0.000170222	0.000054757	-0.000013028
11	1	0.000007412	-0.000000049	-0.000022279
12	15	-0.000036117	0.000010362	-0.000048266
13	8	-0.000058468	0.000008568	0.000037338
14	8	-0.000041113	-0.000201394	-0.000099659
15	8	-0.000046525	-0.000046236	0.000037796
16	6	0.000093338	-0.000071526	0.000160984
17	1	-0.000022498	0.000014946	-0.000004778
18	6	0.000060433	0.000012125	0.000020371
19	1	0.000008519	0.000070914	-0.000013974
20	1	0.000001160	0.000007979	0.000012657
21	1	-0.000003619	0.000005716	-0.000019598
22	1	0.000013530	0.000012331	0.000013265
23	8	0.000151017	0.000009659	-0.000048937
24	6	-0.000011563	0.000049020	0.000062341
25	1	0.000012666	0.000015645	-0.000003908
26	1	-0.000032891	-0.000028067	0.000011179
27	1	0.000019082	0.000013145	-0.000025990
28	6	-0.000096360	0.000035903	-0.000021789

29	6	-0.000133914	0.000136313	-0.000167440
30	1	0.000075428	-0.000018530	-0.000000486
31	1	-0.000019031	-0.000016580	0.000056552
32	1	0.000013266	-0.000117313	-0.000036377
33	1	0.000045104	-0.000076239	-0.000013375
34	1	0.000021712	0.000048125	0.000088984
35	1	0.000057041	-0.000025209	0.000074284

5c

Center Number	Atomic Number	Forces (Hartrees/Bohr) X	Y	Z
1	6	-0.000000837	0.000001376	-0.000000876
2	6	-0.000001345	0.000000608	-0.000000743
3	6	-0.000000602	0.000000854	-0.000000770
4	6	0.000000185	-0.000000163	-0.000002316
5	6	-0.000001370	0.000000238	-0.000000918
6	6	0.000001128	0.000002555	0.000000328
7	1	-0.000000643	0.000002110	-0.000001205
8	1	-0.000001139	0.000000250	-0.000001083
9	1	-0.000000817	-0.000001438	-0.000000296
10	6	-0.000000124	0.000000085	0.000001414
11	8	0.000000641	-0.000004291	0.000001070
12	1	-0.000001467	-0.000002112	0.000000131
13	15	0.000004037	0.000002672	0.000004460
14	8	-0.000000661	0.000000426	-0.000001423
15	8	0.000004081	-0.000006593	0.000001736
16	8	-0.000004331	0.000000533	0.000005379
17	6	0.000006148	-0.000005703	-0.000004440
18	1	-0.000000894	-0.000002467	0.000000959
19	6	-0.000004122	0.000003705	-0.000002503
20	1	0.000000627	-0.000000338	0.000001807
21	1	0.000002251	-0.000000584	0.000001247
22	1	0.000002373	0.000000432	0.000002259
23	16	-0.000002795	0.000004525	-0.000002470
24	8	-0.000000791	-0.000001038	-0.000002937
25	8	-0.000002211	-0.000003696	-0.000000956
26	6	-0.000001879	0.000000606	-0.000002238
27	1	-0.000002111	0.000000134	-0.000002212
28	1	-0.000001573	-0.000000213	-0.000001779
29	1	-0.000002895	0.000000282	-0.000000964
30	1	0.000000157	0.000001404	0.000000016
31	8	-0.000000939	0.000001736	-0.000001829
32	6	0.000000217	0.000004225	-0.000001223
33	1	0.000000084	0.000003461	-0.000000817
34	1	0.000000008	0.000004473	-0.000001161
35	1	-0.000000937	0.000003578	-0.000001939
36	6	0.000000145	-0.000001405	0.000000405

37	6	0.000000876	-0.000000453	0.000005316
38	1	0.000003486	-0.000000905	0.000001153
39	1	0.000002505	0.000000194	0.000001356
40	1	0.000002170	-0.000001185	0.000001778
41	1	0.000000778	-0.000003202	0.000001196
42	1	0.000001787	-0.000001018	0.000002026
43	1	0.000000799	-0.000003658	0.000003062

3OMe_carbocation

Center Number	Atomic Number	Forces (Hartrees/Bohr) X	Y	Z
1	6	0.000003891	0.000004908	-0.000000126
2	6	-0.000007624	0.000008770	0.000000853
3	6	0.000001127	0.000005198	-0.000000989
4	6	-0.000004335	-0.000011937	-0.000001596
5	6	-0.000001949	0.000008886	0.000000873
6	6	0.000000106	0.000012176	0.000004445
7	1	-0.000005917	0.000008198	-0.000001935
8	1	-0.000007005	0.000003927	-0.000001397
9	6	-0.000012342	0.000012396	-0.000003890
10	1	0.000001318	-0.000003583	0.000000763
11	15	0.000008182	-0.000015951	0.000008192
12	8	0.000007607	-0.000009091	0.000001586
13	8	-0.000006111	0.000017217	-0.000001779
14	8	0.000008503	0.000003793	-0.000016507
15	6	-0.000020550	-0.000013250	0.000005498
16	6	-0.000006446	-0.000024738	0.000013632
17	1	0.000005595	-0.000006414	0.000000323
18	1	0.000006124	-0.000010385	-0.000001860
19	1	0.000002865	0.000001797	-0.000002918
20	1	0.000006850	-0.000001297	0.000001616
21	1	-0.000000622	0.000006358	0.000001077
22	1	0.000004916	-0.000000328	0.000003384
23	8	0.000007526	0.000005985	-0.000007911
24	6	-0.000002838	0.000014269	0.000009268
25	1	0.000003486	0.000010109	0.000003237
26	1	0.000006563	0.000009256	0.000005206
27	1	0.000000962	0.000011172	0.000003712
28	6	0.000007207	-0.000013494	-0.000004976
29	6	0.000014335	0.000000415	-0.000007979
30	1	-0.000004803	-0.000008580	-0.000004767
31	1	-0.000000323	-0.000011493	-0.000003183
32	1	-0.000006536	-0.000005813	-0.000001202
33	1	-0.000004595	0.000001191	-0.000000742
34	1	-0.000004747	-0.000005207	-0.000001754
35	1	-0.000000421	-0.000004458	0.000001847

5g

Center Number	Atomic Number	Forces (Hartrees/Bohr) X	Y	Z
1	6	0.000003220	0.000001612	0.000004092
2	6	0.000001316	-0.000002324	-0.000000582
3	6	-0.000000548	-0.000003232	-0.000004090
4	6	-0.000002351	-0.000004346	0.000000548
5	6	0.000003795	-0.000006706	0.000006050
6	6	-0.000001885	-0.000002831	0.000006728
7	1	0.000000275	0.000000543	-0.000005306
8	1	-0.000001149	-0.000003066	-0.000008931
9	6	0.000001671	-0.000004860	-0.000005392
10	1	0.000001085	-0.000006821	-0.000008899
11	15	-0.000005095	0.000000869	0.000000330
12	8	0.000003346	-0.000000349	0.000004147
13	8	0.000001653	-0.000005239	-0.000006695
14	8	-0.000000548	0.000003833	-0.000002081
15	6	-0.000002639	0.000010433	-0.000000155
16	1	0.000000570	0.000007687	0.000005123
17	6	0.000002163	-0.000000110	-0.000005206
18	1	-0.000003098	-0.000001231	-0.000008518
19	1	-0.000000636	0.000006593	0.000000226
20	1	-0.000002753	0.000013097	-0.000002037
21	1	0.000000399	-0.000000215	-0.000002476
22	1	-0.000002353	0.000005012	-0.000006015
23	17	0.000000856	-0.000014476	-0.000000115
24	1	0.000001557	-0.000005251	0.000008025
25	1	0.000003197	-0.000002142	0.000012407
26	8	0.000000535	0.000001563	0.000007320
27	6	-0.000001998	0.000001961	0.000003796
28	1	0.000000038	-0.000000115	-0.000000074
29	1	0.000000225	0.000003820	0.000007228
30	1	-0.000000848	0.000006291	0.000000552

7

Center Number	Atomic Number	Forces (Hartrees/Bohr) X	Y	Z
1	6	0.000008302	-0.000014508	-0.000003333
2	6	-0.000002917	-0.000005348	-0.000004573
3	6	0.000005009	-0.000007203	-0.000002421
4	6	-0.000002898	-0.000004217	0.000002406
5	6	0.000003246	-0.000003963	-0.000000935
6	6	-0.000003495	-0.000003609	0.000000185
7	1	0.000004818	-0.000010357	-0.000003246
8	1	0.000002963	-0.000004806	-0.000002931

9	1	-0.000001446	-0.000004754	-0.000000477
10	6	0.000000720	0.000001351	-0.000004299
11	1	0.000001937	0.000000017	0.000001161
12	15	0.000012021	0.000000403	0.000001536
13	8	-0.000000513	0.000006249	-0.000001830
14	8	-0.000012410	-0.000012062	0.000012461
15	8	-0.000005026	0.000008258	0.000006290
16	6	0.000010378	0.000011539	-0.000005538
17	1	0.000001698	0.000007805	0.000002030
18	6	0.000000264	0.000024278	-0.000003595
19	1	0.000002326	0.000010948	0.000002938
20	1	-0.000006860	-0.000000645	0.000004906
21	1	-0.000009176	0.000003107	0.000003002
22	1	-0.000000814	-0.000001321	-0.000000881
23	8	-0.000010093	-0.000014231	-0.000003841
24	6	0.000006548	-0.000005696	-0.000007885
25	1	-0.000002927	-0.000016336	-0.000004216
26	1	-0.000000250	-0.000015628	-0.000003764
27	1	0.000003149	-0.000013990	-0.000004987
28	6	0.000008410	0.000013878	0.000011786
29	6	-0.000004597	-0.000000354	0.000001479
30	1	0.000006189	0.000014236	-0.000001009
31	1	0.000005948	0.000010150	-0.000002829
32	1	0.000008316	0.000007759	-0.000001283
33	1	-0.000008376	0.000008963	0.000006164
34	1	-0.000011460	0.000006753	0.000003164
35	1	-0.000008985	0.000003331	0.000004360

5a

Center Number	Atomic Number	Forces (Hartrees/Bohr) X	Y	Z
1	6	0.000000492	-0.000000688	0.000001488
2	6	-0.000001328	0.000000816	-0.000000205
3	6	0.000003024	-0.000000444	0.000001363
4	6	-0.000003593	-0.000002606	0.000002010
5	6	0.000001327	-0.000000961	-0.000000505
6	6	0.000001164	-0.000001003	-0.000001219
7	1	-0.000000499	-0.000001188	0.000001959
8	1	-0.000000220	0.000000895	0.000000383
9	1	-0.000000986	0.000002030	-0.000000442
10	1	-0.000000044	-0.000002426	0.000001119
11	6	-0.000003666	-0.000000108	0.000005635
12	8	0.000000300	0.000019426	0.000008503
13	1	-0.000004754	0.000003275	-0.000000431
14	15	-0.000018193	-0.000017471	-0.000001983
15	8	0.000001631	0.000000995	0.000001695
16	8	0.000006777	0.000004844	-0.000020159

17	8	0.000008350	0.000008319	0.000017246
18	6	0.000005025	0.000003869	-0.000011122
19	1	0.000001422	-0.000002052	0.000000027
20	6	0.000002667	0.000000236	0.000008589
21	1	-0.000002007	0.000000475	-0.000001438
22	1	0.000002545	0.000000232	-0.000000007
23	1	0.000000067	0.000001580	0.000003838
24	16	0.000006948	-0.000013341	-0.000023485
25	8	-0.000001103	-0.000007021	-0.000002546
26	8	-0.000000633	0.000003896	0.000002857
27	6	-0.000000272	-0.000004799	-0.000000648
28	1	-0.000000219	0.000002088	-0.000002855
29	1	-0.000002969	0.000001665	-0.000003705
30	1	-0.000000608	0.000000325	-0.000003911
31	1	-0.000000484	-0.000002257	0.000001457
32	6	-0.000002446	0.000002286	0.000003822
33	6	-0.000000507	0.000001873	0.000001883
34	1	0.000000503	-0.000000625	0.000006141
35	1	0.000000258	-0.000000078	0.000004431
36	1	0.000000129	0.000000350	0.000004934
37	1	0.000000628	-0.000000060	-0.000000397
38	1	0.000001004	0.000001088	-0.000003084
39	1	0.000000268	-0.000003435	-0.000001239

H_carbocation

Center Number	Atomic Number	Forces (Hartrees/Bohr) X	Y	Z
1	6	0.000010560	-0.000000955	-0.000037925
2	6	-0.000006190	-0.000029367	-0.000021642
3	6	0.000021866	0.000000564	-0.000012418
4	6	-0.000029119	-0.000047060	-0.000013991
5	6	0.000033057	0.000037523	0.000011645
6	6	-0.000017843	0.000027156	0.000025473
7	1	0.000007021	0.000001301	-0.000017113
8	1	-0.000007608	-0.000016687	-0.000034593
9	1	0.000008172	0.000006291	-0.000004285
10	6	-0.000020574	0.000025779	0.000026698
11	1	0.000015540	-0.000005946	-0.000013625
12	15	0.000029448	-0.000008777	0.000025478
13	8	0.000006149	0.000044896	-0.000013694
14	8	0.000007505	-0.000039696	-0.000021909
15	8	-0.000061024	0.000049293	-0.000035931
16	6	0.000039408	0.000043870	-0.000021404
17	1	-0.000003212	0.000009738	0.000015287
18	6	-0.000059260	0.000012841	-0.000006051
19	1	-0.000024806	0.000001048	0.000025508
20	1	0.000002728	-0.000010008	0.000013137

21	1	0.000009047	-0.000016494	0.000041821
22	1	0.000004481	-0.000016801	0.000002748
23	1	0.000000591	0.000011981	-0.000009869
24	6	0.000003111	0.000018020	0.000097335
25	6	0.000045955	-0.000090335	0.000020790
26	1	-0.000017797	-0.000016261	0.000003076
27	1	-0.000000844	-0.000034053	0.000030199
28	1	0.000000600	-0.000021044	-0.000008470
29	1	0.000001071	0.000011580	-0.000026167
30	1	0.000003362	0.000018283	-0.000019737
31	1	-0.000001392	0.000033320	-0.000020371

$TEAH^+$

Center Number	Atomic Number	Forces (Hartrees/Bohr) X	Y	Z
1	7	-0.000017937	-0.000015865	0.000014730
2	1	0.000004935	0.000000254	-0.000000733
3	6	-0.000001784	-0.000005858	-0.000030890
4	1	0.000001040	0.000002822	0.000013640
5	1	0.000003395	0.000004122	0.000006749
6	6	-0.000012432	0.000001558	-0.000013645
7	1	0.000004981	-0.000000651	0.000009638
8	1	0.000014094	0.000004957	0.000005480
9	6	0.000021570	0.000012235	-0.000015030
10	1	-0.000002437	0.000002695	0.000000770
11	1	-0.000003518	-0.000005079	-0.000000813
12	6	-0.000005124	0.000001307	-0.000000730
13	6	0.000003878	0.000018777	0.000001236
14	6	-0.000011844	-0.000009420	0.000013347
15	1	0.000003335	-0.000002576	0.000000372
16	1	0.000009603	-0.000000094	-0.000000890
17	1	-0.000004415	0.000002551	0.000004083
18	1	-0.000002783	0.000003433	-0.000002259
19	1	0.000004787	-0.000006459	0.000007556
20	1	-0.000008753	-0.000003282	0.000001894
21	1	0.000000804	-0.000001775	-0.000001687
22	1	-0.000002631	-0.000001387	-0.000005962
23	1	0.000001238	-0.000002266	-0.000006854

$TEAH^+$_OMs^-

Center Number	Atomic Number	Forces (Hartrees/Bohr) X	Y	Z
1	7	-0.000019796	0.000000537	-0.000025519
2	1	0.000021553	0.000004418	0.000084906

3	6	-0.000001329	0.000006532	0.000013601
4	1	-0.000001710	-0.000000757	-0.000006309
5	1	0.000008366	-0.000000013	-0.000004335
6	6	0.000020315	-0.000005108	0.000001459
7	1	-0.000004471	-0.000005901	-0.000004735
8	1	-0.000000897	0.000000877	-0.000001861
9	6	-0.000026232	-0.000001704	-0.000006292
10	1	0.000008108	-0.000001561	-0.000005191
11	1	0.000002973	-0.000001114	0.000008239
12	6	-0.000019550	0.000009855	-0.000000903
13	6	-0.000000800	-0.000000165	-0.000010945
14	6	0.000006616	-0.000008006	0.000023307
15	1	0.000002319	-0.000005655	0.000000643
16	1	0.000005292	0.000000497	-0.000003806
17	1	0.000002638	0.000005591	0.000001553
18	1	-0.000003140	-0.000004194	0.000006609
19	1	-0.000001083	0.000000125	0.000002050
20	1	-0.000001072	-0.000005865	0.000006523
21	1	0.000002697	-0.000003637	-0.000007286
22	1	-0.000005894	0.000001024	-0.000011914
23	1	-0.000005941	-0.000002654	-0.000003986
24	16	0.000019933	-0.000055677	-0.000035877
25	8	-0.000014712	0.000007580	0.000012615
26	8	0.000014479	0.000001995	0.000004993
27	6	-0.000008340	0.000018402	0.000014802
28	1	0.000003245	-0.000003617	0.000001460
29	1	-0.000001446	0.000002721	0.000002738
30	1	0.000003494	0.000007378	-0.000009313
31	8	-0.000005615	0.000038098	-0.000047224
